# The histone methyltransferase SUV420H2 regulates brown and beige adipocyte thermogenesis

**DOI:** 10.1172/jci.insight.164771

**Published:** 2024-05-07

**Authors:** Xin Cui, Qiang Cao, Fenfen Li, Jia Jing, Zhixue Liu, Xiaosong Yang, Gary J. Schwartz, Liqing Yu, Huidong Shi, Hang Shi, Bingzhong Xue

**Affiliations:** 1 Department of Biology, Georgia State University, Atlanta, Georgia, USA.; 2 Department of Medicine, Albert Einstein College of Medicine, Bronx, New York, USA.; 3 Department of Medicine, University of Maryland School of Medicine, Baltimore, Maryland, USA.; 4 Georgia Cancer Center and; 5 Department of Biochemistry and Molecular Biology, Medical College of Georgia, Augusta University, Augusta, Georgia, USA.

**Keywords:** Metabolism, Adipose tissue, Obesity, Uncoupling proteins

## Abstract

Activation of brown adipose tissue (BAT) thermogenesis increases energy expenditure and alleviates obesity. Here we discover that histone methyltransferase suppressor of variegation 4–20 homolog 2 (*Suv420h2)* expression parallels that of *Ucp1* in brown and beige adipocytes and that *Suv420h2* knockdown significantly reduces — whereas *Suv420h2* overexpression significantly increases — *Ucp1* levels in brown adipocytes. *Suv420h2* knockout (H2KO) mice exhibit impaired cold-induced thermogenesis and are prone to diet-induced obesity. In contrast, mice with specific overexpression of *Suv420h2* in adipocytes display enhanced cold-induced thermogenesis and are resistant to diet-induced obesity. Further study shows that *Suv420h2* catalyzes H4K20 trimethylation at eukaryotic translation initiation factor 4E-binding protein 1 (*4e-bp1*) promoter, leading to downregulated expression of *4e-bp1*, a negative regulator of the translation initiation complex. This in turn upregulates PGC1α protein levels, and this upregulation is associated with increased expression of thermogenic program. We conclude that *Suv420h2* is a key regulator of brown/beige adipocyte development and thermogenesis.

## Abstract 2

SUV420H2 regulates mitochondrial energetics and brown/beige adipocyte thermogenesis via downregulation of 4E-BP1 and subsequent upregulation of PGC1α.

## Introduction

Obesity is a risk factor for a panel of metabolic disorders, including insulin resistance/type 2 diabetes, hypertension, fatty liver diseases, dyslipidemia, cardiovascular diseases, and certain types of cancer. Persistent energy imbalance due to excess energy intake over energy expenditure results in obesity. The total energy expenditure can be divided into basic metabolic rate, physical activity, and adaptive thermogenesis (1). Brown fat is a major player in adaptive thermogenesis (2, 3) due to the unique presence of UCP1 in mitochondrial inner membrane, which uncouples oxidative phosphorylation from ATP synthesis, thereby dissipating energy as heat (2, 3). Recent studies also point to several UCP1-independent mechanisms in thermogenesis (4, 5). Rodents have 2 types of brown adipocytes: classic brown adipose tissue (BAT) is mainly confined to interscapular area, and newly discovered beige adipocytes (BeAT), or beige fat, is sporadically dispersed in white adipose tissue (WAT) and can be induced by β-adrenergic activation (6–8).

Activation of brown/beige adipocyte thermogenesis increases energy expenditure and ameliorates obesity (9, 10). Given the recent discovery of thermogenic brown fat in humans (11–13), it is conceivable that brown/beige adipocyte thermogenesis is a promising target for therapeutic treatment of obesity.

Epigenetic mechanisms, including histone modifications, have emerged as key links between environmental factors (e.g., diets) and complex diseases (e.g., obesity). However, how epigenetic mechanisms regulate brown/beige adipocyte function have been less explored. To identify functional epigenetic markers that regulate brown/beige adipocyte development, we surveyed the expression of most epigenetic enzymes, including histone methyltransferases, demethylases, and histone deacetylases, that catalyze histone methylation and acetylation during the early postnatal development of mouse beige adipocytes, and we found that the expression pattern of suppressor of variegation 4–20 homolog 2 (*Suv420h2*) (*Drosophila*) mirrors that of *Ucp1*. Using genetic mouse models with loss or gain of functions of *Suv420h2*, we determined the role of *Suv420h2* in cold-induced thermogenesis, energy metabolism, and diet-induced obesity.

## Results

### Suv420h2 is important in regulating Ucp1 expression.

**Figure 1:**
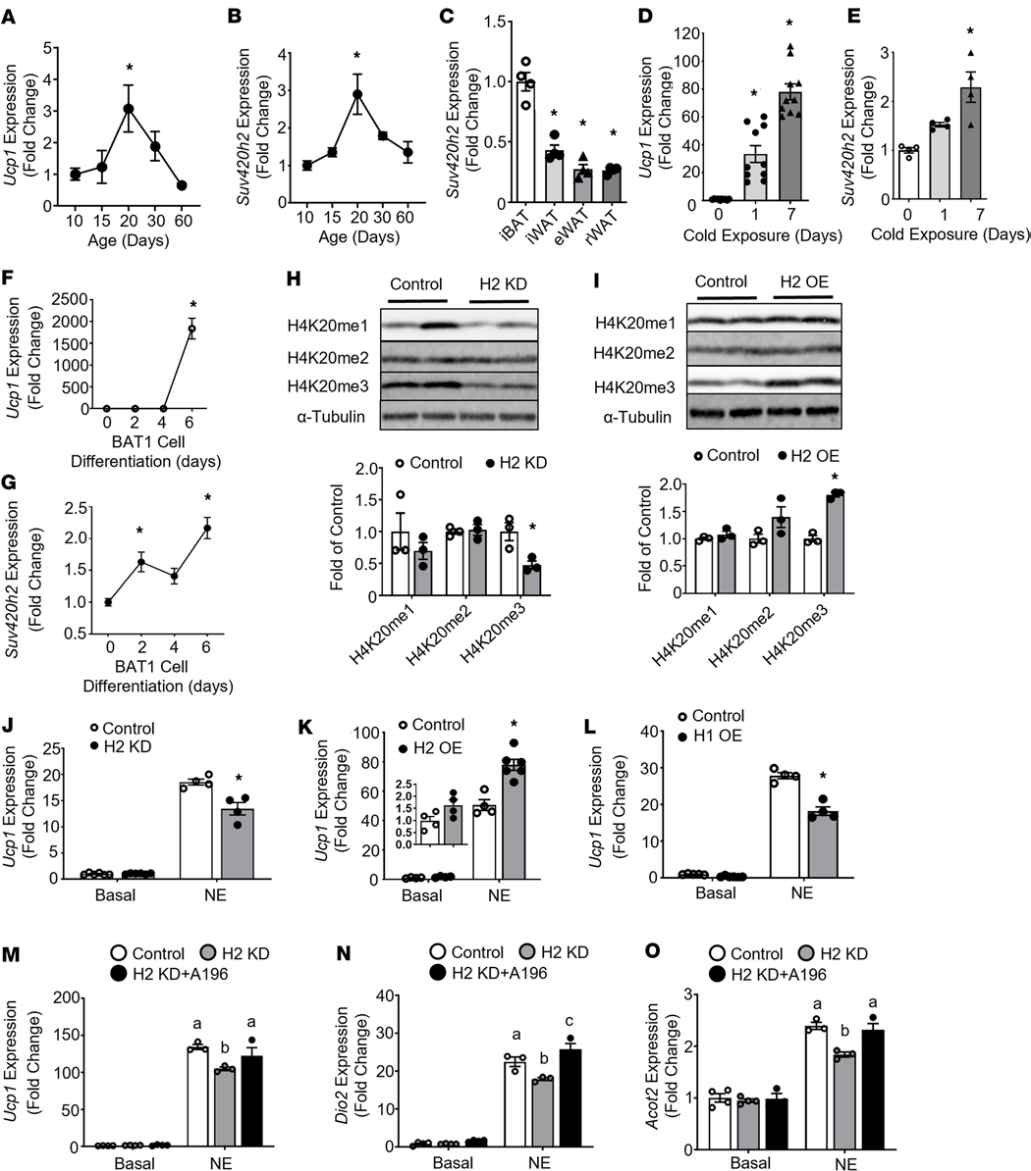
*Suv420h2* regulates Ucp1 expression. (**A** and **B**) *Ucp1* (**A**) and *Suv420h2* (**B**) expression in iWAT of male C57BL/6J mice during postnatal development, *n* = 4–5/group. (**C**) *Suv420h2* expression in brown and white adipose tissues in 2- to 3-month-old male C57BL/6J mice, *n* = 4/group. (**D** and **E**) *Ucp1* (**D**) and *Suv420h2* (**E**) expression in iWAT of 2- to 3-month-old male C57BL/6J mice during a 5°C cold challenge, *n* = 10/group in **D** and *n* = 4/group in **E**. (**F** and **G**) *Ucp1* (**F**) and *Suv420h2* (**G**) expression during BAT1 brown adipocyte differentiation, *n* = 4–6/group. (**H** and **I**) H4K20 mono-, di-, and trimethylation levels in BAT1 brown adipocytes with scramble siRNA (Control) and *Suv420h2* (H2KD) siRNA knockdown (**H**) or with empty vector (Control) and *Suv420h2* (H2OE) overexpression (**I**), *n* = 3/group. Blots were run in parallel at the same time. (**J** and **K**) *Ucp1* expression in BAT1 brown adipocytes with *Suv420h2* knockdown (**J**) or *Suv420h2* overexpression (**K**), *n* = 4–6/group. (**L**) *Ucp1* expression in BAT1 brown adipocytes with overexpression of empty vector (Control) or *Suv420h1* (H1OE), *n* = 4-5/group. (**M**–**O**) *Ucp1* (**M**), *Dio2* (**N**), and *Acot2* (**O**) expression in BAT1 brown adipocytes with *Suv420h2* knockdown and further treated with vehicle (dimethyl sulfoxide [DMSO]) or the SUV420H1/H2 inhibitor A196. Four-day differentiated BAT1 cells were treated with scramble or *Suv420h2* siRNA via electroporation. On day 6 of differentiation, cells were further treated with DMSO or A196 (5 μM) for 24 hours. Before harvesting, cells were further treated with PBS or NE (1 μm) for 4 hours, *n* = 3–4/group. Control: Scramble siRNA+DMSO; H2KD: Suv420h2 siRNA+DMSO; H2KD+A196: Suv420h2 siRNA+A196. All data are expressed as mean ± SEM. *P < 0.05 by 1-way ANOVA followed by Tukey’s multiple-comparison test in **A**–**G**; *P < 0.05 by unpaired 2-tailed Student’s *t* test in (**H**–**I**); *P < 0.05 by 2-way ANOVA followed by Tukey’s multiple-comparison test in **J**–**L**; in **M**–**O**, bars with a different letter indicate statistical significance at *P* < 0.05 as analyzed by 2-way ANOVA followed by Tukey’s multiple-comparison test.

Xue et al. previously reported that beige adipocytes in WAT can be transiently induced in mice during early postnatal development, which peaked at 20 days of age and gradually disappeared thereafter (14). Although the mechanism underlying the transient induction of the developmental beige adipocytes remains unclear, the expression pattern of *Ucp1* in WAT during this period offers a unique framework for identifying factors that regulate brown/beige cell development. Thus, we surveyed the expression patterns of most epigenetic enzymes, including histone methyltransferases, demethylases, and deacetylases, in mouse inguinal WAT (iWAT) during postnatal development from P5 to P120, and compared them with those of *Ucp1*. For the preliminary screening, we pooled 4 RNA samples from each time point (14). We found that *Ucp1* expression in iWAT during postnatal development followed similar patterns as those observed in retroperitoneal WAT (rWAT) (14), peaked at P20, and gradually disappeared afterward (Supplemental Figure 1A; supplemental material available online with this article; https://doi.org/10.1172/jci.insight.164771DS1). Among the 4 genes encoding histone methyltransferases (*Suv420h1*, *Suv420h2*, and SET domain containing protein 8 [*Setd8*], and demethylase PHD finger protein 8 [*Phf8*]) that are responsible for histone H4 lysine 20 (H4K20) methylation, we discovered a unique expression pattern of *Suv420h2* (Supplemental Figure 1, B–E), which mimicked that of *Ucp1*. We then further confirmed our results on *Ucp1* and *Suv420h2* expression using 4 individual RNA samples (Figure 1, A and B). In adult rodents, *Suv420h2* expression was much higher in interscapular BAT (iBAT) than in other fat depots, including iWAT, epididymal WAT (eWAT), and rWAT (Figure 1C). We also found that *Suv420h2* expression was much higher than that of *Suv420h1* in adipose tissues (Supplemental Figure 1F). As expected, a 7-day cold exposure at 5°C in 2- to 3-month-old male mice stimulated *Ucp1* expression in iWAT (Figure 1D). Interestingly, *Suv420h2* expression parallels that of *Ucp1* in iWAT during cold exposure (Figure 1E).

In addition, differentiation of mouse immortalized brown adipocyte cell BAT1 (15, 16) is characterized by significant upregulation of *Ucp1* expression (Figure 1F), which is paralleled by a gradual increase of *Suv420h2* expression (Figure 1G).

H4K20 can be mono-, di-, and trimethylated (H4K20me1, H4K20me2, and H4K20me3, respectively) (17, 18). SETD8 is the only known monomethyltransferase, whereas SUV420H1 and SUV420H2 are responsible for di- and trimethylation of H4K20 (17, 18). We therefore further studied the roles of *Suv420h1*, *Suv420h2*, and in general H4K20 methylation in regulating brown/beige adipocyte function. We found that knocking down *Suv420h2* with siRNA in BAT1 cells (Supplemental Figure 2) significantly decreased H4K20me3 levels around 50% without affecting H4K20me1 and H4K20me2 levels (Figure 1H), whereas overexpressing *Suv420h2* significantly increased H4K20me3 levels in BAT1 cells without changing H4K20me1 or H4K20me2 (Figure 1I). Interestingly, knocking down *Suv420h2* in BAT1 cells significantly suppressed, whereas overexpressing *Suv420h2* significantly enhanced, NE-stimulated *Ucp1* expression (Figure 1, J and K).

Since knocking down *Suv420h2* resulted in around 50% reduction of H4K20me3 and since both SUV420H1 and SUV420H2 catalyze H4K20 trimethylation, we also explored possible physiological function of *Suv420h1* in regulating brown adipocyte thermogenesis. Interestingly, overexpressing *Suv420h1* (Supplemental Figure 3) resulted in significantly decreased NE-stimulated *Ucp1* expression (Figure 1L), indicating that *Suv420h1* and *Suv420h2* may have opposite effects on brown adipocyte thermogenic function. To further explore this possibility, we knocked down *Sub420h2* in BAT1 brown adipocytes and further treated cells with SUV420H1/H2 inhibitor A196, which has been shown to achieve an 80% reduction of H4K20me3 levels in treated cells (19). *Suv420h2* knockdown significantly reduced *Suv420h2* expression without changing *Suv420h1* levels in BAT1 cells, whereas combined *Suv420h2* knockdown and A196 treatment did not change *Suv420h1* expression nor did it further change *Suv420h2* expression (Supplemental Figure 4, A and B). As expected, knocking down *Suv420h2* significantly suppressed NE-stimulated expression of genes important for brown adipocyte thermogenesis, including *Ucp1* (Figure 1M), type 2 deiodinase (*Dio2*) (Figure 1N), and acyl-CoA thioesterase 2 (*Acot2*) (Figure 1O), a gene shown to facilitate mitochondrial fatty acid oxidation (20). Interestingly, combined treatment of BAT1 cells with *Suv420h2* knockdown and A196 reversed the inhibitory effects of *Suv420h2* knockdown on these gene expression levels and restored them to those of the control group (Figure 1, M–O). These data collectively demonstrate that *Suv420h1* and *Suv420h2* regulate brown adipocyte thermogenesis, with *Suv420h2* serving as a potential positive regulator, whereas *suv420h1* may negatively regulate brown adipocyte thermogenesis.

### Suv420h2 regulates the development of brown and beige fat.

**Figure 2:**
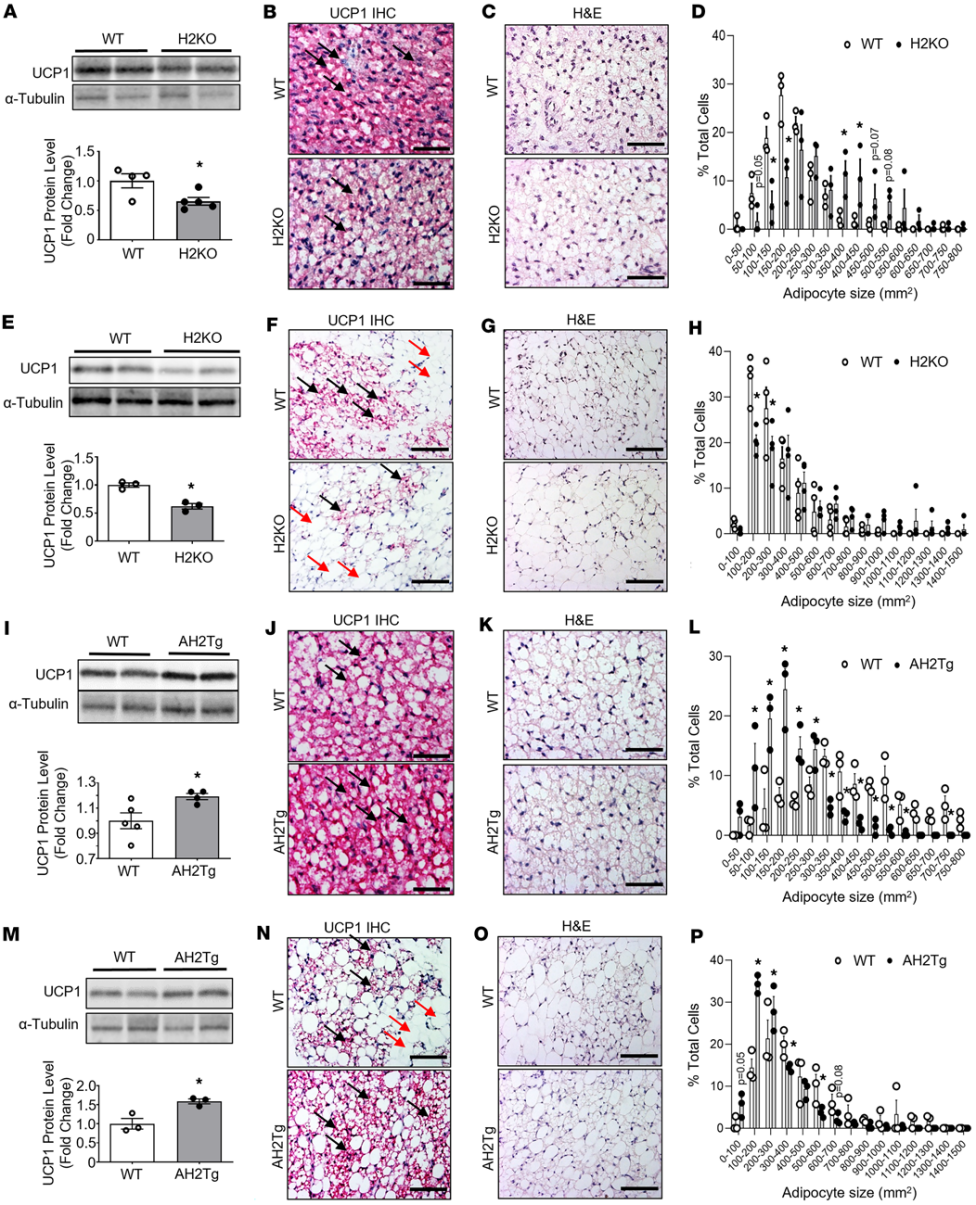
*Suv420h2* regulates brown and beige fat development. (**A**–**D**) UCP1 protein levels (**A**), UCP1 immunostaining (**B**), H&E staining (**C**), and adipocyte size (**D**) in iBAT of 20-day-old H2KO and WT mice. In **A**, *n* = 4–5/group; in **D**, *n* = 3/group. In **B**, images are representatives from 3 replicate animals/group. Images from additional animals are located in Supplemental Figure 6A. Scale bar: 70µm in **B** and **C**. (**E**–**H**) UCP1 protein levels (**E**), UCP1 immunostaining (**F**), H&E staining (**G**) ,and adipocyte size (**H**) in iWAT of 20-day-old H2KO and WT mice. In **E**, *n* = 3/group; in **H**, *n* = 4/group. In **F**, images are representatives from 3 replicate animals/group. Images from additional animals are located in Supplemental Figure 6, B and C. Scale bar: 140 µm in **F** and **G**. (**I**–**L**) UCP1 protein levels (**I**), UCP1-immunostaining (**J**), H&E staining (**K**), and adipocyte size (**L**) in iBAT of 20-day-old AH2Tg and WT mice. In **I**, *n* = 4–5/group; in **L**, *n* = 3/group. In **J**, images are representatives from 3 replicate animals/group). Images from additional animals are located in Supplemental Figure 8A. Scale bar: 70 µm in **J** and **K**. (**M**–**P**) UCP1 protein levels (**M**), UCP1-immunostaining (**N**), H&E staining (**O**), and adipocyte size (**P**) in iWAT of 20-day-old AH2Tg and WT mice. In **M** and **P**, *n* = 3/group. In **N**, images are representatives from 3 replicate animals/group. Images from additional animals are located in Supplemental Figure 8, B and C. Scale bar: 140 µm in **N** and **O**. All data are expressed as mean ± SEM. UCP1^+^ multilocular brown/beige adipocytes are shown in dark purplish red color and are indicated with black arrows; UCP1^–^ unilocular white adipocytes are shown in light color and are indicated with red arrows. *P < 0.05 by unpaired 2-tailed Student’s *t* test in **A**, **E**, **I**, and **M**; *P < 0.05 as analyzed by 2-way ANOVA followed by Tukey’s multiple-comparison test in **D**, **H**, **L**, and **P**.

Recent data suggest that mice lacking both *Suv420h1* and *Suv420h2* exhibited increased mitochondria respiration in brown adipocytes, improved glucose tolerance, and were resistant to diet-induced obesity (21). However, since our in vitro data suggest that *Suv420h1* and *Suv420h2* may exert opposite effects on brown adipocyte function, it is important to delineate the functions of *Suv420h1* and *Suv420h2* separately in mouse models. Our gene expression data suggest that *Suv420h2* mirrors *Ucp1* expression during the postnatal development of beige adipocytes; we thus interrogated the role of *Suv420h2* in the development of brown and beige adipocytes in vivo. We first examined brown and beige adipose tissue development in mice with whole body *Suv420h2* KO (H2KO) (22) at P20, when the developmental beige adipocytes appear at peak while brown fat development also ascends to maturity (14). As expected, Suv420h2 mRNA was not detectable in fat depots of H2KO mice, including iBAT, iWAT, eWAT and rWAT; in addition, there was also no difference in adipose tissue *Suv420h1* expression between WT and H2KO mice (Supplemental Figure 5, A and B). Interestingly, iBAT from H2KO mice had significantly decreased UCP1 protein expression and less UCP1 staining compared with that of WT controls (Figure 2, A and B and Supplemental Figure 6A). This was associated with enlarged adipocyte size (Figure 2C), as shown by a shift of significantly decreased smaller adipocyte and reciprocally increased larger adipocyte numbers in iBAT of 20-day-old H2KO mice compared with WT mice (Figure 2D). Likewise, iWAT from 20-day-old H2KO mice also had significantly lower UCP1 protein expression (Figure 2E) and less multilocular beige adipocytes with UCP1 staining (Figure 2F and Supplemental Figure 6, B and C), suggesting less appearance of the developmental beige adipocytes in iWAT of H2KO mice. iWAT from 20-day-old H2KO mice had enlarged adipocyte size (Figure 2G), with a shift of significantly decreased smaller adipocyte and a tendency of reciprocally increased larger adipocyte numbers (Figure 2H).

We also generated transgenic mice (AH2Tg mice) overexpressing *Suv420h2* specifically in adipocytes under the control of adiponectin promoter (Supplemental Figure 7A). AH2Tg mice exhibited a significant increase of *Suv420h2* mRNA in all fat depots, including iBAT, iWAT, eWAT and rWAT, without affecting *Suv420h1* levels (Supplemental Figure 7, B and C). iBAT from 20-day-old AH2Tg mice exhibited enhanced UCP1 protein levels and more UCP1 staining (Figure 2, I and J, and Supplemental Figure 8A). In addition, overexpression of *Suv420h2* in adipocytes resulted in reduced adipocyte size in iBAT during postnatal development at P20 (Figure 2K), as shown by a shift of significantly increased smaller adipocyte and a reciprocally decreased larger adipocyte number (Figure 2L). AH2Tg mice exhibited higher UCP1 protein levels and more UCP1^+^ multilocular beige adipocytes in iWAT (Figure 2, M and N, and Supplemental Figure 8, B and C). iWAT from AH2Tg mice also exhibited reduced adipocyte size (Figure 2O), as shown by a shift of significantly increased smaller adipocyte and reciprocally decreased larger adipocyte number (Figure 2P). These data suggest that *Suv420h2* promotes brown and beige adipocytes formation during the postnatal development.

### Suv420h2 regulates cold-induced thermogenesis.

**Figure 3:**
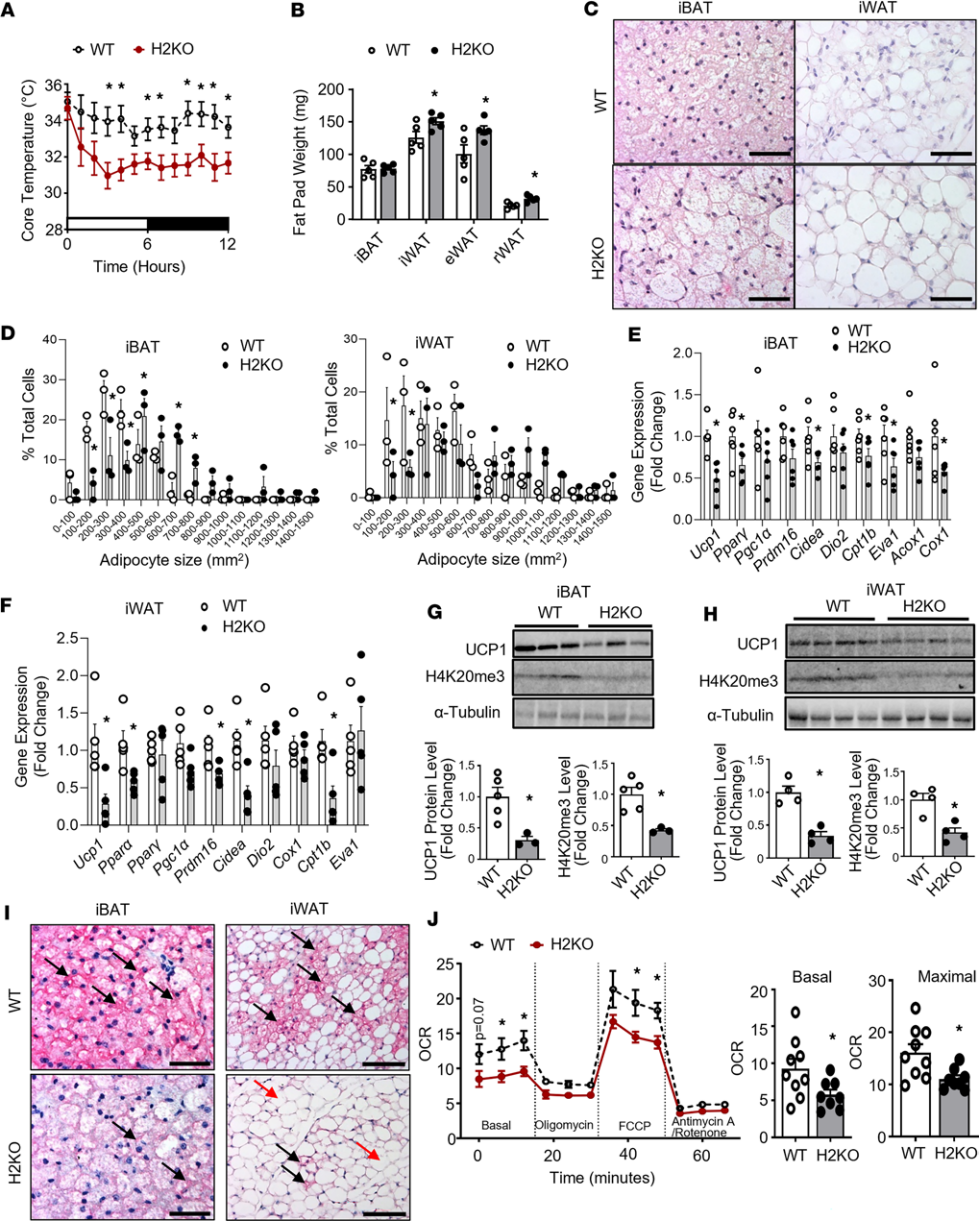
H2KO mice have impaired cold-induced thermogenesis. (**A** and **B**) Core body temperature (**A**) and fat pad weight (**B**) in 3-month-old male WT and H2KO mice during a 7-day cold challenge at 5°C. In **A**, *n* = 5–7/group; in **B**, *n* = 5/group. (**C** and **D**) H&E staining (**C**) and quantification of adipocyte size (**D**) in iBAT and iWAT of WT and H2KO mice after the 7-day cold challenge. In **C**, scale bar: 70 µm for iBAT and 140 μm for iWAT; In **D**, *n* = 3/group. (**E** and **F**) Gene expression analysis in iBAT (**E**) and iWAT (**F**) of WT and H2KO mice after the 7-day cold challenge, *n* = 6/group in **E** and *n* = 5/group in **F**. (**G** and **H**) UCP1 protein and H4K20me3 levels in iBAT (**G**) and iWAT (**H**) of WT and H2KO mice after the 7-day cold challenge, *n* = 5 (WT) and *n* = 3 (H2KO) in **G** and *n* = 4/group in **H**. (**I**) UCP1 immunostaining in iBAT and iWAT of WT and H2KO mice after the 7-day cold challenge (representative images from 3 replicate animals/group). Images from additional animals can be found in Supplemental Figure 9, A–C. UCP1^+^ multilocular brown/beige adipocytes are shown in dark purplish red color and are indicated with black arrows; UCP1^–^ unilocular white adipocytes are shown in light color and are indicated with red arrows. Scale bar: 70 µm for iBAT and 140 μm for iWAT. (**J**) Oxygen consumption rate (OCR) in primary brown adipocytes isolated from iBAT of male WT and H2KO mice measured by a Seahorse XF 96 Extracellular Flux Analyzer, *n* = 9 (WT) and 8 (H2KO). All data are expressed as mean ± SEM. *P < 0.05 by 2-way ANOVA with repeated measures followed by Tukey’s multiple-comparison test in **A** and left panel of **J**; *P < 0.05 by unpaired 2-tailed Student’s *t* test in **B**, **E**–**H**, and right 2 panels of **J**; *P < 0.05 by 2-way ANOVA followed by Tukey’s multiple-comparison test in **D**.

In adult mice, beige adipocytes can be induced by chronic cold exposure. To determine the role of *Suv420h2* in cold-induced brown and beige adipocyte thermogenesis, we subjected 3-month-old male H2KO, AH2Tg, and their respective WT littermates to a chronic 7-day cold challenge. During the cold exposure, H2KO mice displayed significantly lower body temperature compared with their littermate controls (Figure 3A), suggesting that *Suv420h2* deficiency causes cold intolerance. Moreover, H2KO mice had higher fat mass in iWAT, eWAT, and rWAT after the cold challenge (Figure 3B), suggesting less efficiency in utilizing stored energy in fat depots. This was consistent with larger adipocytes observed in both iBAT and iWAT of H2KO mice (Figure 3C), with a shift of reduced smaller adipocyte and reciprocally increased larger adipocyte numbers in both iBAT and iWAT of cold-challenged H2KO mice, although the increase of larger adipocyte numbers in iWAT did not reach statistical significance (Figure 3D). In addition, cold-challenged H2KO mice exhibited decreased expression of *Ucp1* in both iBAT and iWAT (Figure 3, E and F), along with reduced expression of other cold-induced thermogenic genes, including peroxisome proliferator activated receptor γ (*Pparγ*), cell death–inducing DNA fragmentation factor, α subunit-like effector A (*Cidea)*, muscle type carnitine palmitoyltransferase 1b (*Cpt1b*), epithelial V-like antigen 1 (*Eva1*), palmitoyl acyl-Coenzyme A oxidase 1 (*Acox1*), and cytochrome c oxidase subunit I (*Cox1*) in iBAT and *Ppar*α, PR domain containing 16 (*Prdm16*), *Cidea*, and *Cpt1b* in iWAT (Figure 3, E and F). As expected, *Suv420h2* deficiency resulted in decreased H4K20me3 levels in both iBAT and iWAT, along with decreased UCP1 protein levels (Figure 3, G and H). Consistent with these findings, IHC analysis revealed less UCP1 staining in iBAT and less UCP1^+^ beige adipocytes in iWAT of cold-challenged H2KO mice (Figure 3I and Supplemental Figure 9, A–C). Seahorse analysis of primarily isolated brown adipocytes revealed reduced basal and maximal oxygen consumption rate (OCR) in H2KO mice relative to WT controls (Figure 3J), suggesting that *Suv420h2* deletion compromised mitochondrial function in a cell-autonomous manner.

**Figure 4:**
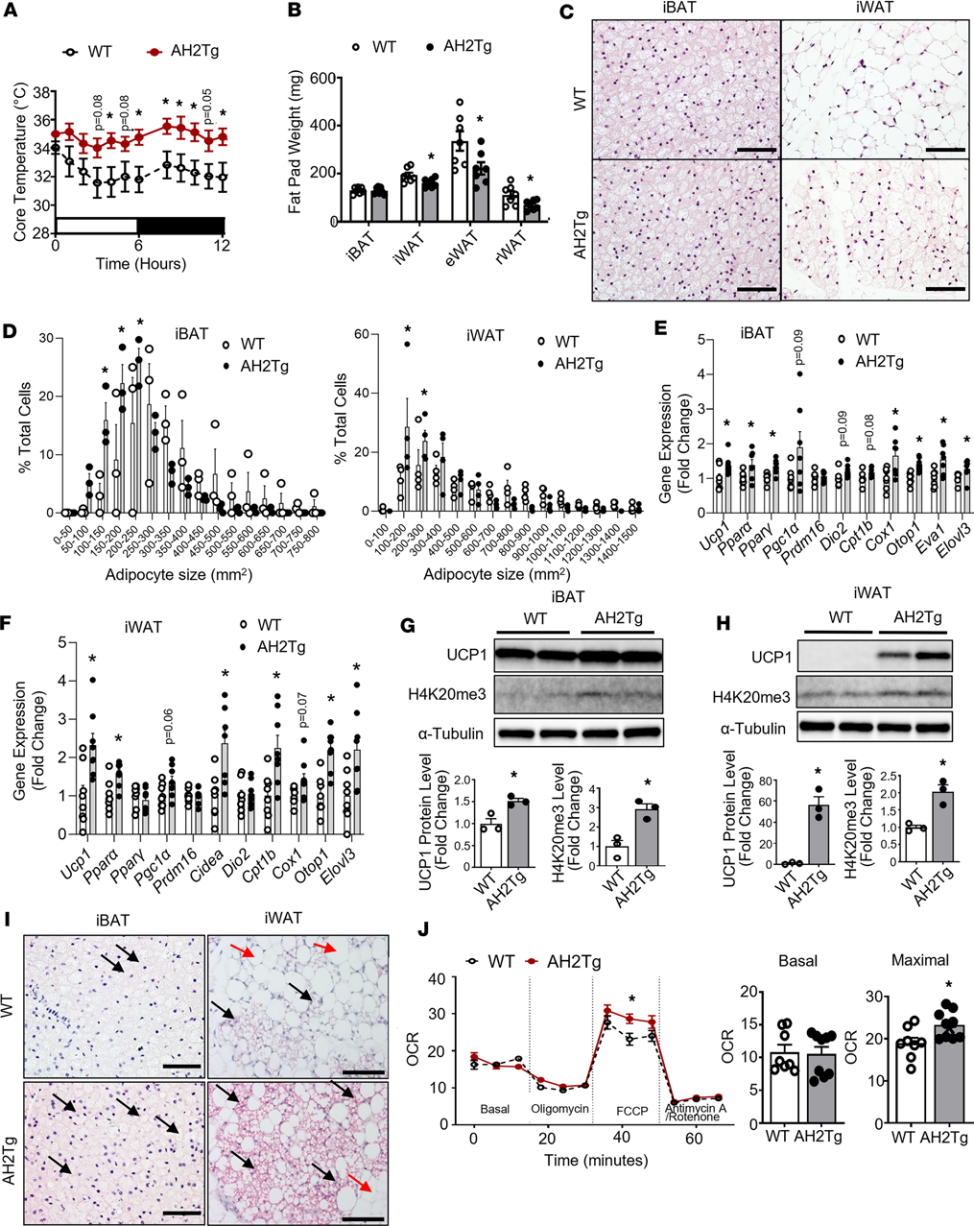
AH2Tg mice have enhanced cold-induced thermogenesis. (**A** and **B**) Core body temperature (**A**) and fat pad weight (**B**) in 3-month-old male WT and AH2Tg mice during a 7-day cold challenge at 5°C. In **A**, *n* = 6–7/group. In **B**, *n* = 7/group. (**C** and **D**) H&E staining (**C**) and adipocyte size (**D**) in iBAT and iWAT of WT and AH2Tg mice after the 7-day cold challenge. In **C**, scale bar: 70µm for iBAT and 140μm for iWAT. In **D**, *n* = 3/group in iBAT and *n* = 4/group in iWAT. (**E** and **F**) Gene expression analysis in iBAT (**E**) and iWAT (**F**) of WT and AH2Tg mice after the 7-day cold challenge, *n* = 7/group in **E** and *n* = 8/group in **F**.(**G** and **H**) UCP1 protein and H4K20me3 levels in iBAT (**G**) and iWAT (**H**) of WT and AH2Tg mice after the 7-day cold challenge, *n* = 3/group. (**I**) UCP1 immunostaining in iBAT and iWAT of WT and AH2Tg mice after the 7-day cold challenge (representative images from 3 replicate animals/group). Images from additional animals can be found in Supplemental Figure 10, A–C. UCP1^+^ multilocular brown/beige adipocytes are shown in dark purplish red color and are indicated with black arrows; UCP1^–^ unilocular white adipocytes are shown in light color and are indicated with red arrows. Scale bar: 70 µm for iBAT and 140 μm for iWAT. (**J**) Oxygen consumption rate (OCR) in primary brown adipocytes isolated from iBAT of male WT and AH2Tg mice measured by a Seahorse XF 96 Extracellular Flux Analyzer, *n* = 8 (WT) and 9 (H2KO). All data are expressed as mean ± SEM. *P < 0.05 by 2-way ANOVA with repeated measures followed by Tukey’s multiple-comparison test in **A** and in left panel of **J**; *P < 0.05 by unpaired 2-tailed Student’s *t* test in **E**–**H** and in right 2 panels of **J**; *P < 0.05 by 2-way ANOVA followed by Tukey’s multiple-comparison test in **E**.

In contrast, AH2Tg mice with adipocyte *Suv420h2* overexpression exhibited an opposite phenotype. Specifically, AH2Tg mice displayed higher body temperature compared with their littermate controls during the cold challenge (Figure 4A), suggesting an increased cold tolerance. Cold-challenged AH2Tg mice also had decreased fat mass in iWAT, eWAT, and rWAT (Figure 4B). iBAT and iWAT from cold-challenged AH2Tg mice had smaller adipocytes (Figure 4C), as shown by a shift of significantly increased smaller adipocyte numbers and a tendency of reciprocally decreased larger adipocyte numbers (Figure 4D). In addition, iBAT and iWAT from cold-challenged AH2Tg mice exhibited enhanced expression of *Ucp1* and other thermogenic genes, such as *Pparα*, Ppar*γ*, *Cox1*, Otopetrin 1 (*Otop1*), *Eva1*, and elongation of very long–chain fatty acids (FEN1/Elo2, SUR4/Elo3, yeast) like 3 (*Elovl3*) in iBAT, and *Pparα*, *Cidea*, *Cpt1b*, *Otop1*, and *Elovl3* in iWAT (Figure 4, E and F). Moreover, *Suv420h2* overexpression in adipocytes led to a significant increase in H4K20me3 as well as UCP1 levels in both iBAT and iWAT (Figure 4, G and H). IHC analysis revealed a stronger UCP1 staining in iBAT and higher UCP1^+^ beige adipocyte induction in iWAT (Figure 4I and Supplemental Figure 10, A–C). In addition, Seahorse analysis revealed enhanced maximal OCR in primary adipocytes isolated from AH2Tg mice (Figure 4J), suggesting that *Suv420h2* overexpression increases mitochondrial function in a cell-autonomous manner.

### Suv420h2 regulates mitochondrial bioenergetic program.

**Figure 5:**
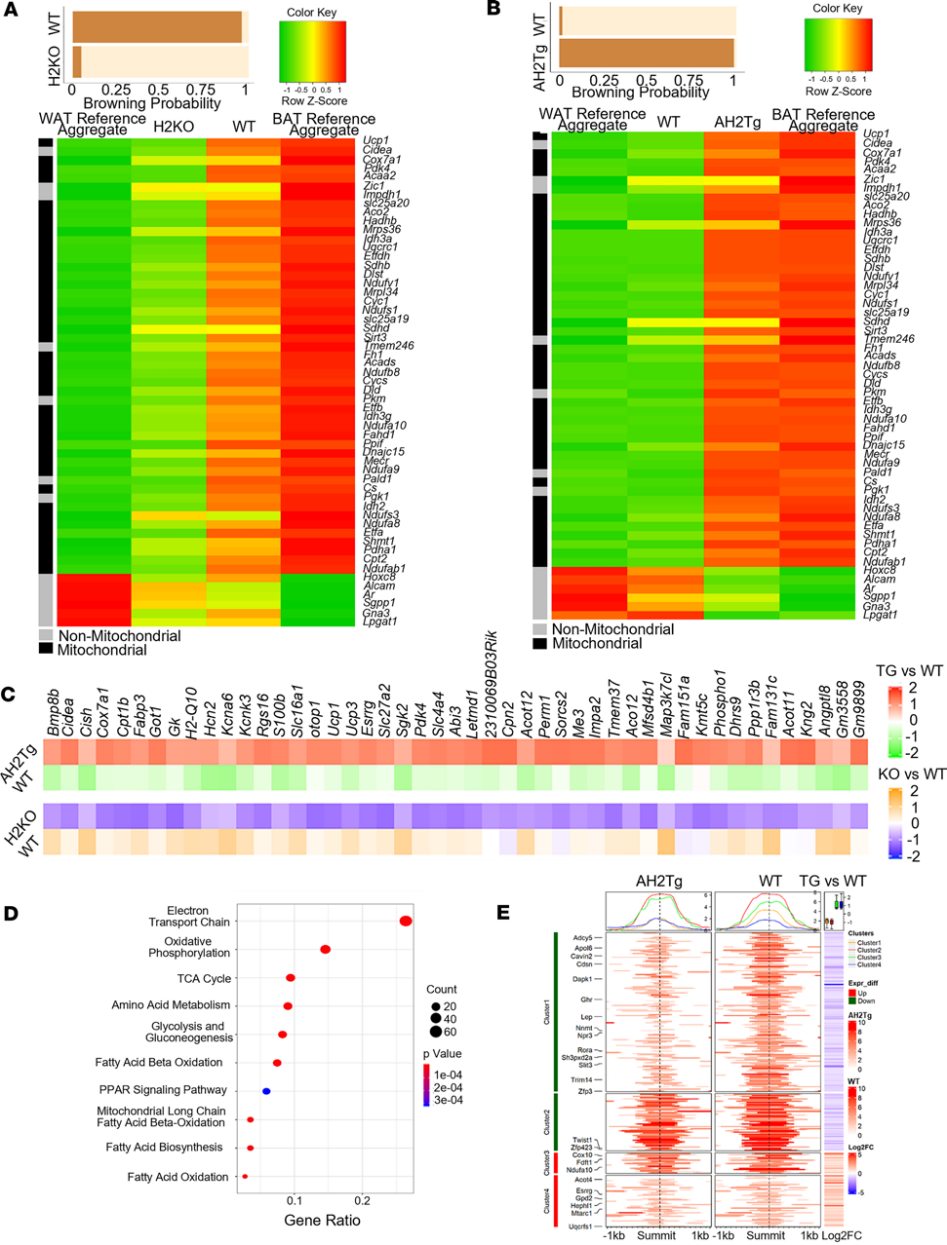
SUV420H2 regulates mitochondrial bioenergetic program. (**A** and **B**) RNA-Seq analysis of BAT-specific gene expression in iWAT of male H2KO mice (**A**) and male AH2Tg mice (**B**) after the 7-day cold exposure using an online software (https://github.com/PerocchiLab/ProFAT). The WAT reference aggregate and BAT reference aggregate were derived from the online software. (**C**) Heatmaps of genes that are reciprocally regulated in iWAT of H2KO and AH2Tg mice after cold exposure. (**D**) Analysis of pathways that are reciprocally regulated in iWAT of H2KO and AH2Tg mice after cold exposure. (**E**) Comparison of genome-wide alterations in chromatin accessibility landscape assessed by ATAC-Seq with the corresponding gene expression assessed by RNA-Seq of AH2Tg and WT mice after the 7-day cold exposure.

To gain further insight into how *Suv420h2* regulates brown/beige fat thermogenesis, we performed RNA-Seq analysis in iWAT of 7-day-cold-challenged H2KO and AH2Tg mice. Analysis of differentially expressed genes with online software (https://github.com/PerocchiLab/ProFAT; commit ID: 84d79da) (23) predicted an overall reduced browning probability in *Suv420h2*-deficient iWAT, with a reciprocal increase in gene expression profile resembling that of WAT (Figure 5A). This was consistent with a downregulation of BAT-specific gene expression and an upregulation of WAT-specific gene expression in *Suv420h2*-deficient iWAT (Figure 5A). In contrast, analysis of differentially expressed genes in iWAT between WT and AH2Tg mice revealed an overall enhanced browning probability, evidenced by enhanced BAT-specific and reduced WAT-specific gene expression (Figure 5B). Interestingly, we found that groups of BAT-specific genes were reciprocally regulated in iWAT between H2KO and AH2Tg mice, including *Ucp1*, *Ucp3*, *Cpt1b*, *Otop1*, *Kcnk3*, and *S100b* (Figure 5, A–C), highlighting the importance of *Suv420h2* in beige fat thermogenesis. More strikingly, genes involved in mitochondrial bioenergetic pathways, including electron transport chain, fatty acid β-oxidation, and TCA cycle stood out as converged pathways that were down- or upregulated in H2KO and AH2Tg mice, respectively (Figure 5D and Supplemental Figure 11, A and B).

To further investigate how SUV420H2 regulates pathways in mitochondria function and thermogenesis, we performed assay for transposase-accessible chromatin sequencing (ATAC-Seq) analysis in iBAT of 7-day-cold-challenged WT and AH2Tg mice. We compared genome-wide alterations in chromatin accessibility landscape assessed by ATAC-Seq with gene expression patterns assessed by RNA-Seq and discovered a strong correlation between chromatin accessibility status and gene expression changes. As illustrated in Figure 5E, the decreases in read densities of genes of 2 selective clusters (Clusters 1 and 2; Figure 5E) based on variable degree of peaks in AH2Tg iBAT, which indicates less chromatin accessibility, were highly associated with the downregulations of the corresponding gene expression; this includes several genes known to negatively regulate brown/beige adipocyte thermogenesis and energy metabolism, such as nicotinamide N-methyltransferase (*Nnmt*) (24), natriuretic peptide receptor 3 (*Npr3*) (25), twist basic helix-loop-helix transcription factor 1 (*Twist1*) (26), and zinc finger protein 423 (*Zfp423*) (27). In addition, we also identified 2 clusters of genes that showed more chromatin accessibility and were associated with increased gene expression (Clusters 3 and 4; Figure 5E), including several genes encoding mitochondrial electron transporting chain proteins, such as complex I component *Ndufa10*, complex III component ubiquinol-cytochrome c reductase, Rieske iron-sulfur polypeptide 1 (*Uqcrfs1*), and complex IV component heme A:farnesyltransferase cytochrome c oxidase assembly factor 10 (*Cox10*).

**Figure 6:**
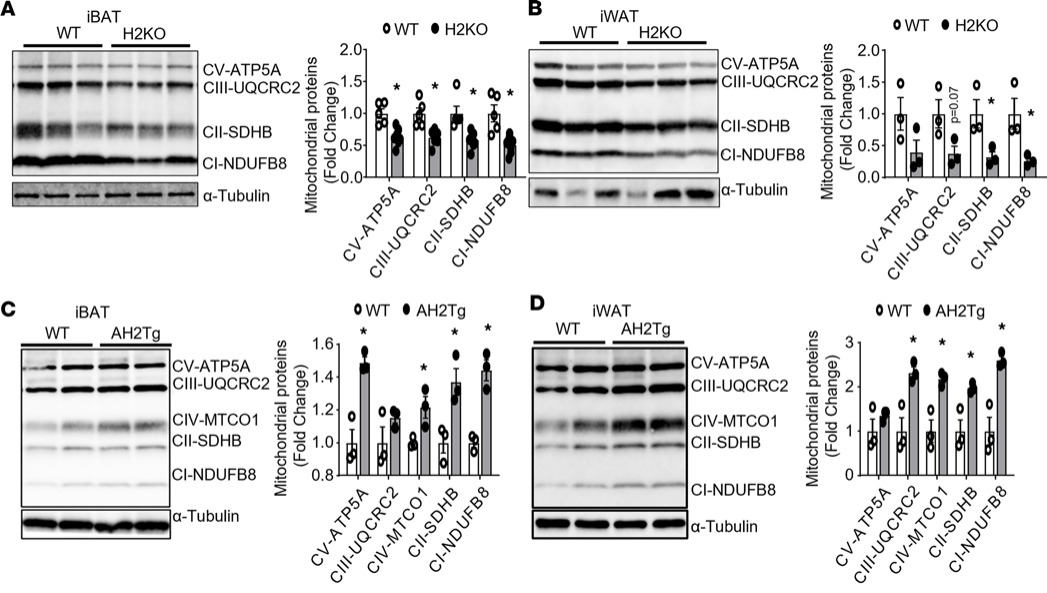
SUV420H2 regulates mitochondrial respiratory chain complex protein levels. (**A** and **B**) Immunoblotting of mitochondrial respiratory chain complex proteins in iBAT (**A**) and iWAT (**B**) of H2KO and WT mice after the 7-day cold exposure. *n* = 5-7/group in **A** and *n* = 3/group in **B**. *P < 0.05 by unpaired 2-tailed Student’s *t* test. (**C** and **D**) Immunoblotting of mitochondrial respiratory chain complex proteins in iBAT (**C**) and iWAT (**D**) of AH2Tg and WT mice after the 7-day cold exposure, *n* = 3/group. *P < 0.05 by unpaired 2-tailed Student’s *t* test. All data are expressed as mean ± SEM.

Our data suggest that Suv420h2 regulates pathways involved in mitochondrial bioenergetics. Indeed, immunoblotting analysis of mitochondrial respiratory chain proteins revealed downregulation of complex I NADH dehydrogenase 1β subcomplex 8 (CI-NDUFB8); complex II succinate dehydrogenase complex, subunit B (CII-SDHB); and complex III cytochrome b-c1 complex subunit 2 (CIII-UQCRC2) in both iBAT and iWAT of H2KO mice (Figure 6, A and B), while revealing upregulation of CI-NDUFB8, CII-SDHB, and complex IV mitochondrially encoded cytochrome c oxidase I (CIV-MTCO1) in iBAT and iWAT of AH2Tg mice during cold exposure (Figure 6, C and D).

### H4K20me3 is elevated at the promoter of 4E-BP1.

**Figure 7:**
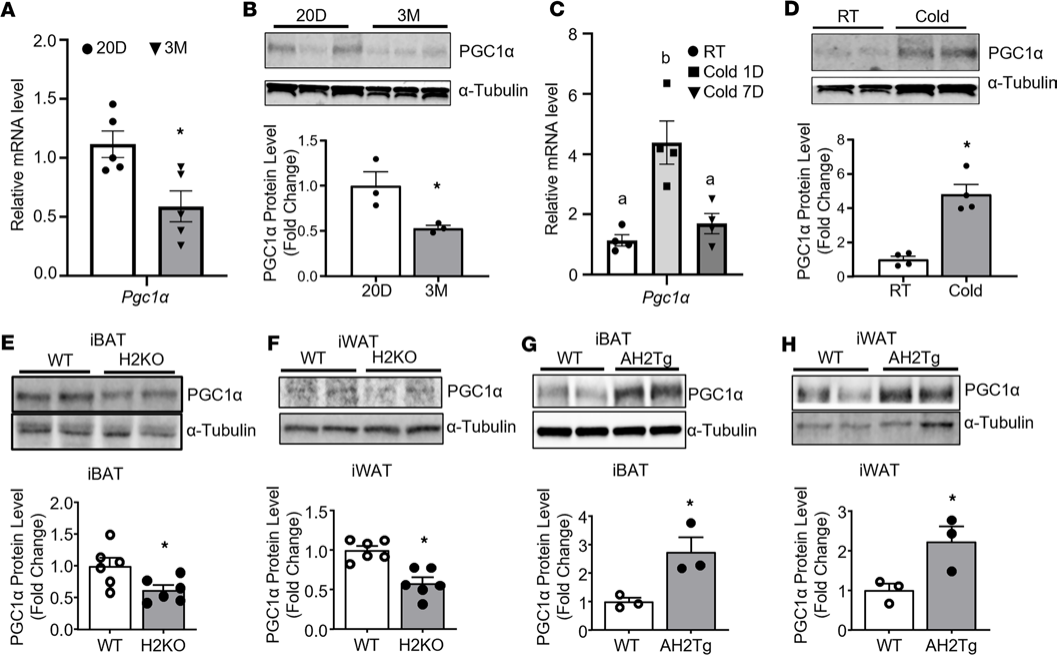
SUV420H2 regulates brown/beige adipocyte thermogenesis through posttranscriptional regulation of PGC1α protein levels. (**A** and **B**) PGC1α mRNA (**A**) and protein (**B**) levels in iWAT of C57B6/6J mice during postnatal development. *n* = 5/group in **A** and *n* = 3/group in **B**. *P < 0.05 by unpaired 2-tailed Student’s *t* test. (**C** and **D**) PGC1α mRNA (**C**) and Protein (**D**) levels in iWAT of C57B6/6J mice during cold exposure. *n* = 4/group. In **C**, bars with a different letter indicate statistical significance at *P* < 0.05 as analyzed by 1-way ANOVA followed by Tukey’s multiple-comparison test; in **D**, *P < 0.05 by unpaired 2-tailed Student’s *t* test. (**E** and **F**) PGC1α protein levels in iBAT (**E**) and iWAT (**F**) of H2KO and WT mice after the 7-day cold exposure. *n* = 6/group. *P < 0.05 by unpaired 2-tailed Student’s *t* test. (**G** and **H**) PGC1α protein levels in iBAT (**G**) and iWAT (**H**) of AH2Tg and WT mice after the 7-day cold exposure. *n* = 3/group. *P < 0.05 by unpaired 2-tailed Student’s *t* test. All data are expressed as mean ± SEM.

Since genes responsible for mitochondrial function appear to be the most significant feature of *Suv420h2* regulated pathways, we next explored whether *Pgc1α*, the master regulator of mitochondrial biogenesis (28), is involved in this process. We first studied whether *Pgc1α* mRNA and protein levels were regulated during postnatal development and cold exposure. While both *Pgc1α* mRNA and protein levels were significantly higher in iWAT of 20-day-old mice compared with those of 3-month-old mice (Figure 7, A and B), H4K20me3 level at *Pgc1α* promoter was not significantly different in iWAT across the developmental course (Supplemental Figure 12). Furthermore, while *Pgc1α* mRNA expression was only transiently upregulated in iWAT 1 day after cold exposure, cold-induced increase in PGC1α protein levels was observed at 7 days after cold exposure (Figure 7, C and D). These data suggest that *Pgc1α* expression may not depend on promoter H4K20 trimethylation and PGC1α protein level may be regulated independently of mRNA expression, at least during chronic cold exposure.

Similarly, although our ATAC-Seq and RNA-Seq data suggest that overexpressing *Suv420h2* in adipocytes resulted in a more open chromatin structure at *Pgc1α* locus, along with increased *Pgc1α* expression peaks (Supplemental Figure 13A), quantitative PCR (qPCR) analysis showed that *Pgc1α* expression was not significantly changed in iBAT and iWAT of H2KO (Figure 3, E and F) or AH2Tg mice (Figure 4, E and F) after cold exposure. We also did not observe any changes in *Pgc1α* expression in BAT1 brown adipocytes with *Suv420h2* knockdown and with combined *Suv420h2* knockdown and A196 treatment (Supplemental Figure 13B). Interestingly, *Suv420h2* deletion in H2KO mice decreased, while Suv420h2 overexpression in AH2Tg mice increased, PGC1α protein content in both iBAT and iWAT (Figure 7, E–H). Thus, our data suggest that PGC1α protein level may be regulated independently of its mRNA expression and that Suv420h2 may be involved in the regulation of PGC1α protein levels.

**Figure 8:**
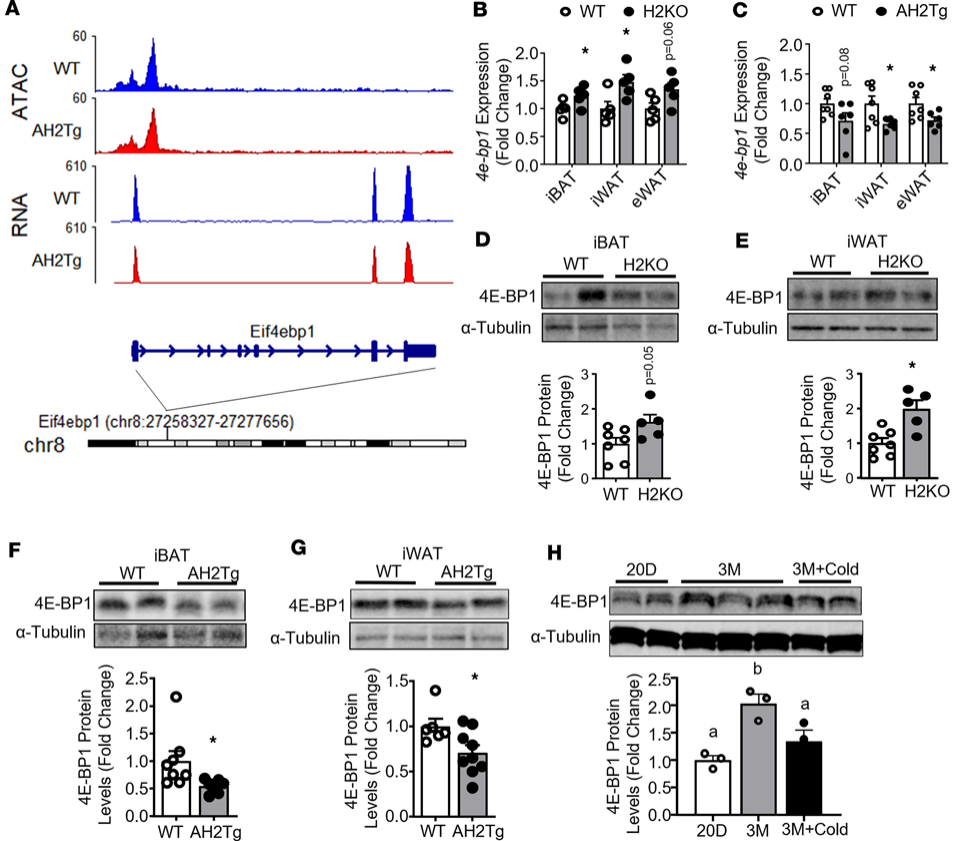
4E-BP1 mRNA and protein levels are reciprocally regulated in H2KO and AH2Tg animals after cold exposure. (**A**) ATAC-Seq analysis of chromatin accessibility and RNA-Seq peak data at *4e-bp1* gene locus in AH2Tg and WT mice after a 7-day cold exposure. (**B** and **C**) Expression of *4e-bp1* in various adipose tissues of H2KO (**B**) and AH2Tg (**C**) mice after cold exposure. *n* = 5/group in **B**, and *n* = 7 (WT) and 6 (AH2Tg) in **C**. *P < 0.05 by unpaired 2-tailed Student’s *t* test. (**D** and **E**) 4E-BP1 protein levels in iBAT (**D**) and iWAT (**E**) of H2KO and WT mice after the 7-day cold exposure. *n* = 7 (WT) and 5 (H2KO). *P < 0.05 by unpaired 2-tailed Student’s *t* test. Blots were run in parallel at the same time. (**F** and **G**) 4E-BP1 protein levels in iBAT (**F**) and iWAT (**G**) of AH2Tg and WT mice after the 7-day cold exposure. *n* = 8 (WT) and 7 (AH2Tg) in **F**, and *n* = 6 (WT) and 9 (AH2Tg) in **G**. *P < 0.05 by unpaired 2-tailed Student’s *t* test. (**H**) 4E-BP1 protein levels in iWAT of C57BL/6J mice during postnatal development and after a cold challenge. *n* = 3/group. Bars with a different letter indicate statistical significance at *P* < 0.05 as analyzed by 1-way ANOVA followed by Tukey’s multiple-comparison test. Blots were run in parallel at the same time. All data are expressed as mean ± SEM.

PGC1α is a short-lived protein; therefore, its protein level is tightly regulated by either protein synthesis or degradation. PGC1α protein levels can be regulated by protein degradation (29, 30) or synthesis (31). The E3 ligases F-box and WD-40 domain protein 7 (FBXW7) and ring finger protein 34 (RNF34) have been previously shown to promote PGC1α protein ubiquitination and degradation (29, 30), whereas PGC1α protein translation can be regulated by the eukaryotic translation initiation eukaryotic translation initiation factor 4F (eIF4F) complex, since the negative regulator of the elF4F complex, the eukaryotic translation initiation factor 4E binding protein 1 (4E-BP1), has been shown to negatively regulate PGC1α protein synthesis (31). There was no change in the expression of *Fbxw7* and *Rnf34* between WT and H2KO and between WT and H2Tg mice (Supplemental Figure 14) (31). Interestingly, our ATAC-Seq and RNA-Seq data suggest that overexpressing *Suv420h2* in adipocytes resulted in a more closed chromatin structure at *4e-bp1* locus, which was associated with reduced *4e-bp1* expression (Figure 8A). Indeed, *4e-bp1* expression was significantly upregulated in iBAT and iWAT of cold-challenged H2KO mice but tended to decrease in iBAT and was significantly decreased in iWAT and eWAT of cold-challenged AH2Tg mice (Figure 8, B and C). 4E-BP1 protein levels in iBAT and iWAT were increased in cold-challenged H2KO mice but decreased in cold-challenged AH2Tg mice (Figure 8, D–G).

We also measured 4E-BP1 protein levels in iWAT of C57BL/6J mice during postnatal development and cold challenge. Interestingly, 4E-BP1 protein levels were significantly increased in iWAT of 3-month-old mice as compared with 20-day-old mice (Figure 8H). Since 4E-BP1 negatively regulates PGC1α protein levels (31), this may explain the decreased PGC1α protein levels in iWAT of 3-month-old mice (Figure 7B). On the other hand, cold exposure significantly reduced 4E-BP1 protein levels (Figure 8H), which may contribute to the increased PGC1α protein levels in iWAT of cold-challenged mice (Figure 7D).

**Figure 9:**
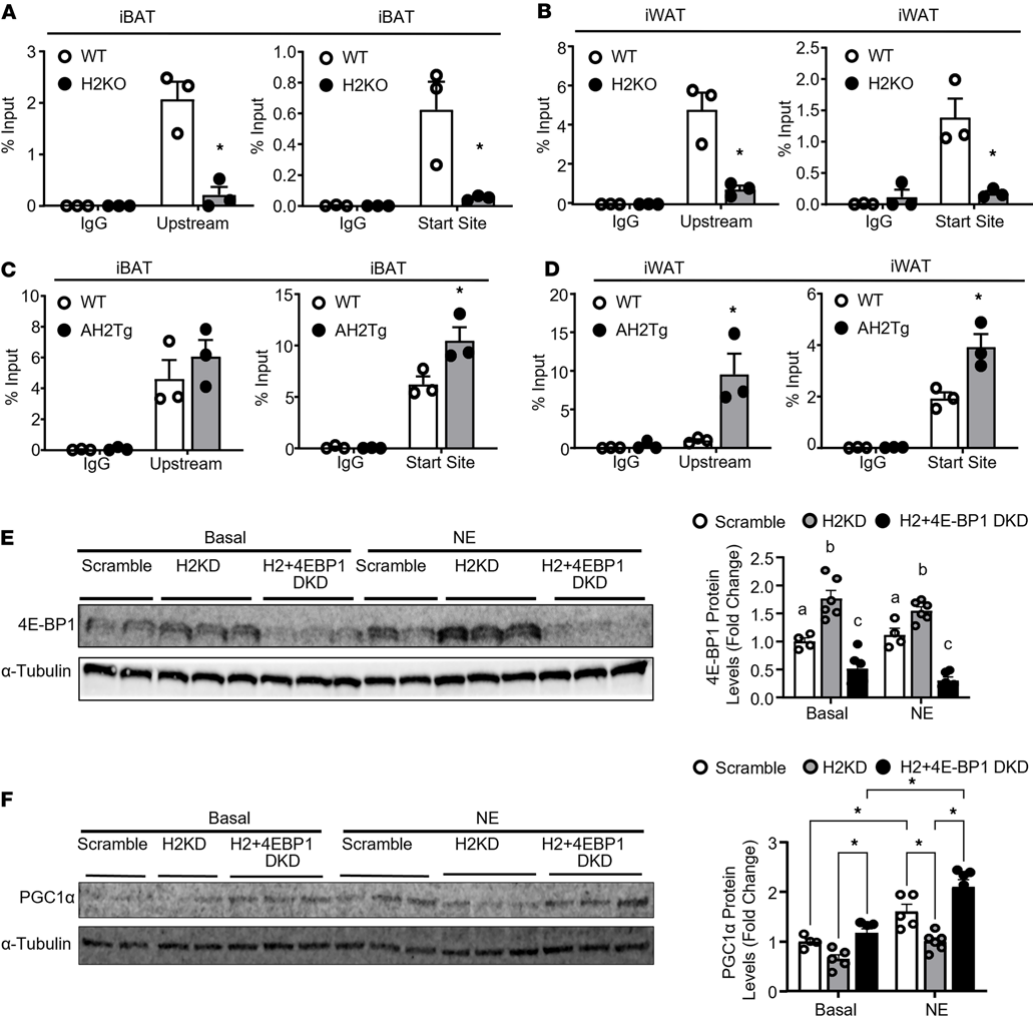
SUV420H2 regulates PGC1α protein levels through increasing H4K20me3 at *4e-bp1* promoter. (**A** and **B**) H4K20me3 levels at the promoter regions of *4e-bp1* as assessed by ChIP assay in iBAT (**A**) and iWAT (**B**) of H2KO and WT mice after a 7-day cold exposure. *n* = 3/group. *P < 0.05 by 2-way ANOVA followed by Tukey’s multiple-comparison test. (**C** and **D**) H4K20me3 levels at the promoter regions of *4e-bp1* as assessed by ChIP assay in iBAT (**C**) and iWAT (**D**) of AH2Tg and WT mice after a 7-day cold exposure. *n* = 3/group. *P < 0.05 by 2-way ANOVA followed by Tukey’s multiple-comparison test. (**E** and **F**) Basal and NE-induced 4E-BP1 (**E**) and PGC1α (**F**) protein levels in BAT1 brown adipocytes treated with either *Suv420h2* knockdown or combined *Suv420h2/4e-bp1* knockdown. *n* = 4–6/group. In **E**, bars with a different letter indicate statistical significance at *P* < 0.05 as analyzed by 2-way ANOVA followed by Tukey’s multiple-comparison test. In **F**, *P < 0.05 by 2-way ANOVA followed by Tukey’s multiple-comparison test. All data are expressed as mean ± SEM.

Mechanistically, ChIP assay reveals that H4K20me3 levels at *4e-bp1* promoter (Supplemental Figure 15) (32–34) were significantly decreased in both iBAT and iWAT of H2KO mice (Figure 9, A and B). Thus, *Suv420h2* deletion may decrease histone repressive mark H4K20me3 at the *4e-bp1* locus, resulting in increased *4e-bp1* expression, which could lead to decreased PGC1α protein levels seen in H2KO mice. In contrast, *Suv420h2* overexpression in AH2Tg mice increased *4e-bp1* promoter H4K20me3 levels in iBAT and iWAT (Figure 9, C and D), and this increase may lead to decreased expression of *4e-bp1*, potentially contributing to increased PGC1α protein levels observed in AH2Tg mice.

To further confirm that SUV420H2 regulates PGC1α protein levels via regulation of *4e-bp1* expression, we knocked down both *Suv420h2* and *4e-bp1* in BAT1 brown adipocytes. As shown in Figure 9E, knocking down *Suv420h2* significantly increased 4E-BP1 levels in BAT1 brown adipocytes, similarly to those observed in H2KO mice (Figure 8, D and E), whereas combined knockdown of both *Suv420h2* and *4e-bp1* significantly reduced 4E-BP1 levels (Figure 9E). Interestingly, knocking down of *Suv420h2* tended to reduce basal PGC1α protein levels and significantly reduced NE-stimulated PGC1α protein levels in BAT1 brown adipocytes. Further knocking down of *4e-bp1* blocked this effect and restored PGC1α protein level to that of control group (Figure 9F). These data suggest that 4E-BP1 mediates SUV420H2’s effect on regulating PGC1α protein levels.

We also explored other possible SUV420H2 downstream targets that could mediate SUV420H2’s function in regulating brown/beige adipocyte function. Pedrotti et al. (21) reported that deletion of both *Suv420h1* and *Suv420h2* resulted in enhanced mitochondria respiration in brown adipocytes, possibly via upregulation of the expression of *Pparγ*, a master regulator of brown and white adipocyte lipid and glucose metabolism, and thermogenic function (35, 36). However, we observed no difference in chromatin accessibility and RNA expression peaks at *Pparγ* locus in our ATAC-Seq and RNA-Seq data from cold-challenged WT and AH2Tg mice (Supplemental Figure 16A). In addition, there were no consistent changes in cold-induced *Pparγ* mRNA (Figures 3, E and F, and Figure 4, E and F) and protein (Supplemental Figure 16, B and C) levels in iBAT and iWAT between WT and H2KO mice and between WT and AH2Tg mice.

*Prdm16* is emerged as an important regulator of brown adipocyte development (16, 37). However, we did not observe any differences in chromatin accessibility and RNA expression at the *Prdm16* locus in our ATAC-Seq and RNA-Seq data (Supplemental Figure 17A). In addition, there were no consistent changes in cold-induced *Prdm16* mRNA (Figure 3, E and F, and Figure 4, E and F) or protein (Supplemental Figure 17B-C) levels in iBAT and iWAT between WT and H2KO mice and between WT and AH2Tg mice.

*Twist1* and *Zfp423* negatively regulate brown/beige adipocyte thermogenesis and energy homeostasis (26, 27). *Twist1* interacts with PGC1α on PGC1α-target genes to suppress mitochondrial metabolism and uncoupling (26), whereas *Zfp423* suppresses adipocyte thermogenic capacity by interfering with several important factors for brown adipocyte function, such as early B cell factor 2 (*Ebf2*) and *Prdm16* (27, 38). Our ATAC-Seq and RNA-Seq data indicate that chromatin accessibility and RNA expression peaks at *Twist1* and *Zfp423* loci (Supplemental Figure 18A and Supplemental Figure 19A) were decreased in WT and AH2Tg mice after cold exposure. In addition, the expression of *Twist 1* (Supplemental Figure 18, B and C) and *Zfp423* (Supplemental Figure 19, B and C) was increased in iWAT of H2KO mice but reciprocally decreased in iWAT of AH2Tg mice after cold exposure. However, ChIP assay demonstrated that H4K20me3 levels at the *Twist1* (Supplemental Figure 18, D and E) or *Zfp423* (Supplemental Figure 19, D and E) promoter were not different in iBAT and iWAT between cold-challenged WT and H2KO mice or between cold-challenged WT and AH2Tg mice. Thus, while changes in *Twist1* and *Zfp423* expression might contribute to altered brown/beige adipocyte function observed in our H2KO and AH2Tg mice, they are not likely mediated via *Suv420h2*-regulated H4K20 methylation.

Estrogen-related receptor γ (*Esrrg*) is emerged as a positive regulator of mitochondrial oxidative metabolism and thermogenesis via both *Pgc1α*-dependent and -independent mechanisms (39, 40). Our ATAC-Seq and RNA-Seq data indicate that chromatin accessibility and RNA expression peaks at the *Esrrg* locus were increased in cold-challenged WT and AH2Tg mice (Supplemental Figure 20A). In addition, *Esrrg* mRNA and protein levels were decreased in iWAT of H2KO mice (Supplemental Figure 20, B and C) but were reciprocally increased in iWAT of AH2Tg mice (Supplemental Figure 20, D and E) after cold exposure. However, the H4K20me3 level at *Esrrg* promoter was not different in iBAT and iWAT between H2KO and WT (Supplemental Figure 20, F and G) or between AH2Tg and WT mice (Supplemental Figure 20, H and I), suggesting that the altered *Esrrg* expression in H2KO and AH2Tg mice was not dependent on *Suv420h2*.

We further investigated whether ESRRG protein level could be regulated by 4E-BP1. As shown in Supplemental Figure 20J, *Suv420h2* knockdown significantly reduced NE-stimulated ESRRG protein levels in BAT1 cells; however, further knockdown of *4e-bp1* blocked this effect and restored ESRRG protein levels to those of the control group. Thus, *Esrrg* may be another potential target besides *Pgc1α* mediating *Suv420h2*’s effect on brown/beige adipocyte thermogenesis. However, similarly to that of *Pgc1α*, *Esrrg* may not be a direct target of *Suv420h2*, since *Esrrg* promoter H4K20me3 levels in iBAT and iWAT were not different in cold-challenged H2KO and AH2Tg mice compared with their respective WT controls. Instead, ESRRG protein levels may be regulated by 4E-BP1–mediated translational regulation, similarly to levels of PGC1α.

We further explored whether other brown/beige adipocyte-related genes could be direct targets for *Suv420h2* by comparing H4K20me3 level at the promoters of several genes in iWAT during postnatal development. However, we did not observe differences in H4K20me3 levels at the promoters of *Ucp1* (Supplemental Figure 21A); RB transcriptional corepressor 1 (*Rb1)* (Supplemental Figure 21B), a negative regulator of brown adipocyte thermogenesis (41); or Krupple-like transcription factor 2 (*Klf2*) (Supplemental Figure 21C), a negative regulator of adipogenesis (42) in iWAT along the postnatal developmental course.

### Suv420h2 is important in the regulation of diet-induced obesity.

To determine the role of *Suv420h2* in diet-induced obesity, we challenged H2KO, AH2Tg, and their respective WT littermates with a high-fat diet (HFD). When housed at ambient room temperature (20°C–22°C), H2KO mice had increased fat mass in iWAT and eWAT despite no change in body weight (Supplemental Figure 22, A and B). This was associated with decreased energy expenditure in H2KO mice evident by reduced oxygen consumption and heat production (Supplemental Figure 22, C and D) without changes in locomotor activity (Supplemental Figure 22E) or food intake (Supplemental Figure 22F).

Similarly, while there was no change in body weight (Supplemental Figure 23A), HFD-challenged AH2Tg mice housed at ambient room temperature had decreased fat mass in eWAT without changes in other fat pads (Supplemental Figure 23B). Ah2Tg mice also exhibited increased energy expenditure, as shown by increased oxygen consumption and heat production (Supplemental Figure 23, C and D) without changes locomotor activity (Supplemental Figure 23E) or food intake (Supplemental Figure 23F).

**Figure 10:**
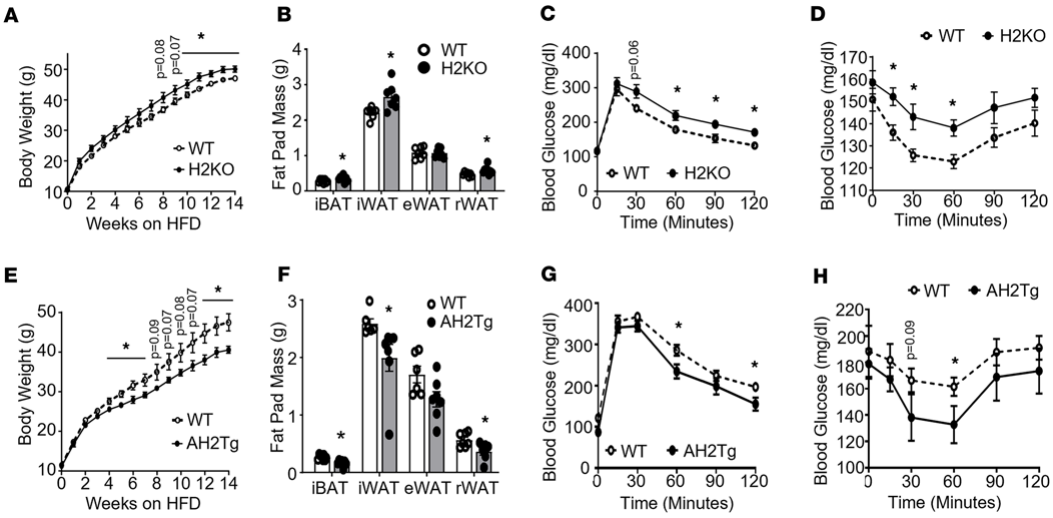
SUV420H2 regulates diet-induced obesity. (**A**–**D**) Body weight (**A**), fat pad mass (**B**), glucose tolerance test (GTT) (**C**), and insulin tolerance test (ITT) (**D**) in H2KO and WT mice fed a HFD when housed at thermoneutralty. In **A** and **B**, *n* = 7/group. *P < 0.05 by 2-way ANOVA with repeated measures followed by Tukey’s multiple-comparison test in **A** and unpaired 2-tailed Student’s *t* test in **B**. In **C** and **D**, *n* = 6–7/group. *P < 0.05 by 2-way ANOVA with repeated measures followed by Tukey’s multiple-comparison test. (**E**–**H**) Body weight (**E**), fat pad mass (**F**), glucose tolerance test (GTT) (**G**), and insulin tolerance test (ITT) (**H**) in AH2Tg and WT mice fed a HFD when housed at thermoneutralty. *n* = 6–7/group. *P < 0.05 by 2-way ANOVA with repeated measures followed by Tukey’s multiple-comparison test in **E**, **G**, and **H**, and unpaired 2-tailed Student’s *t* test in **F**. In **G** and **H**, *n* = 6–7/group. *P < 0.05 by 2-way ANOVA with repeated measures followed by Tukey’s multiple-comparison test. All data are expressed as mean ± SEM.

We previously reported that mild cold stress under ambient room temperature (20°C–22°C) may trigger nonshivering thermogenesis (43). Thus, we also conducted HFD feeding experiments under thermoneutrality (30°C). When housed under thermoneutrality, H2KO mice gained more weight starting after 4 weeks of HFD feeding (Figure 10A) with increased fat mass in iBAT, iWAT, and rWAT depots (Figure 10B), and they exhibited glucose intolerance and insulin resistance assessed by GTT and ITT, respectively (Figure 10, C and D). In contrast, HFD-challenged AH2Tg mice gained less weight under thermoneutrality with lower fat mass in iBAT, iWAT, and rWAT (Figure 10, E and F), and they exhibited improved glucose tolerance and insulin sensitivity as shown by GTT and ITT (Figure 10, G and H). Thus, our data indicate that *Suv420h2* is important in regulating diet-induced obesity.

## Discussion

Xue et al. previously discovered developmentally induced beige adipocytes (14). To identify functional epigenetic marks that regulate brown/beige adipocyte development, we surveyed the expression of epigenetic enzymes responsible for histone modifications during the postnatal development of beige adipocytes and discovered a unique expression pattern of the histone methyltransferase *Suv420h2*, which mirrors that of *Ucp1*. Using genetic models with gain or loss of functions of *Suv420h2*, we demonstrate that *Suv420h2* promotes the development of brown and beige adipocytes postnatally, enhances cold-induced thermogenesis, and prevents diet-induced obesity.

Methylation of H4K20 was one of the first histone modifications to be discovered and is evolutionarily conserved from yeast to humans (17, 18). H4K20 can be mono-, di-, and trimethylated (17, 18). SET8/PR-SET7 is the only known monomethyltransferase, whereas SUV420H1 and SUV420H2 are responsible for the di- and trimethylation of H4K20 (17, 18). The methylation states of H4K20 exert different biological functions. Whereas H4K20me1 and H4K20me2 are involved in DNA replication and DNA damage repair, respectively, H4K20me3 is a hallmark of silenced heterochromatic regions and is also enriched in chromatin regions that contain silenced genes (17, 18, 44). H4K20me3 plays an important role in dynamic biological functions, including development, cellular differentiation, aging, and cancer development (45–49). Here we demonstrate that H4K20me3, catalyzed by SUV420H2, may also be involved in the regulation of brown/beige fat thermogenesis and energy metabolism though the 4E-BP1/PGC1α axis.

The enrichment of genes involved in mitochondrial functions revealed by our RNA-Seq analysis drew our attention to *Pgc1α*, a master regulator of mitochondrial biogenesis and thermogenesis (28). It has been demonstrated that PGC1α protein translation can be regulated by the eukaryotic translation initiation complex (31). The eIF4F complex is composed of eIF4E (mRNA m7GTP 5′ cap-binding protein), eIF4G (a scaffolding protein), and eIF4A (an ATP-dependent RNA helicase) (50). Recognition of the mRNA 5′ cap structure by eIF4E is a rate-limiting step in translational initiation and is, hence, tightly regulated (51). The activity of eIF4E is regulated through interaction with the 3 inhibitory 4E-BPs, 4E-BP1, -2, and -3. The 4E-BPs compete with eIF4G for a shared binding site on eIF4E (52), thereby negatively regulating eIF4F complex formation and translation initiation. Cold exposure downregulates 4E-BP1 expression in BAT, which is mediated through β3-adrenergic agonist–stimulated signaling pathways (53). Importantly, deletion of 4E-BP1 in mice results in greater reduction of adiposity, increased energy expenditure, upregulated *Ucp1* expression, and beige adipocyte induction in WAT; this is primarily due to increased eIF4F complex formation, leading to increased PGC1α protein translation (31). Indeed, we discovered that the *4e-bp1* promoter H4K20me3 level is increased in *Suv420h2*-overexpressing adipocytes, leading to downregulated *4e-bp1* expression and corresponding upregulated PGC1α protein levels. The enhanced PGC1α protein levels may drive the mitochondrial biogenesis in *Suv420h2*-overexpressing adipocytes, resulting in increased brown fat thermogenesis.

SUV420H2 catalyzes the deposition of trimethylation to histone H4k20, which in turn represses gene transcription (17, 18). In the current study, we observed that overexpressing *Suv420h2* increased, whereas H2KO decreased, thermogenic gene expression in brown adipocytes. Thus, we could reasonably predict that SUV420h2 may repress a putative negative regulator of thermogenesis, which in turn promotes thermogenesis. Indeed, we have measured H4K20me3 levels at the promoters of several positive regulators of thermogenesis, including *Pgc1α*, *Pparγ*, *Prdm16*, and *Esrrg*; none of them showed any differences in promoter H4K20me3 level between H2KO and AH2Tg mice, suggesting that they are not direct targets for *Suv420h2*. We have also measured H4K20me3 levels at the promoters of several negative regulators of thermogenesis in adipose tissues, including *4e-bp1*, *Twist1*, *Zfp423*, and *Rb1*. Only *4e-bp1* fit our criteria with a decreased promoter H4K20me3 mark in H2KO mice and reciprocally increased promoter H4K20me3 levels in AH2Tg mice. Future studies with ChIP-Seq using *Suv420h2* or H4K20me3 antibodies are warranted to identify Suv420h2- or H4K20me3-target genes.

In the current study, we also identified PGC1α as one of the targets whose protein synthesis could be regulated by 4E-BP1–dependent regulation of the eukaryotic translation initiation eIF4F complex activity. In addition, whereas *Esrrg* mRNA transcription may not be directly regulated by SUV420H2, our data suggest that ESRRG protein levels may be regulated by 4E-BP1–mediated regulation of the eukaryotic translation initiation eIF4F complex activity, similarly to that of PGC1α. Although 4E-BP1 may regulate the whole translational machinery, the specificity may be regulated in part by specific transcriptional factor complexes on each target gene. Thus, future experiments with ribosome profiling or Ribo-Seq technologies (54, 55) could be performed to identify potential protein candidates that are dependent on SUV420H2/H4K20me3/4E-BP1–regulated cap-dependent protein translation.

Along the course of our study, there were 2 papers published studying the roles of SUV420H1/H2 proteins in brown/beige adipocyte thermogenesis. Pedrotti et al. reported that deletion of both *Suv420h1* and Suv420*h2* in brown adipocytes increased brown fat thermogenesis and ameliorated obesity via activating *Pparγ*-regulated gene networks (21). The results were opposite to what we observed in our genetic models. The exact reason for this discrepancy is not clear. However, different genetic models were used in these 2 studies. For our purpose to distinguish the functions of *Suv420h2* from that of *Suv420h1*, we used animal models with *Suv420h2* deletion without affecting the expression of *Suv420h1*, whereas Pedrotti et al. (21) used animal models with *Suv420h1/Suv420h2* double deletion. Interestingly, we observed that either *Suv420h2* deletion or *Suv420h1* overexpression suppressed brown adipocyte thermogenic gene expression, suggesting that, whereas *Suv420h2* may positively regulate brown adipocyte thermogenesis, *Suv420h1* may serve as a negative regulator. Thus, a possible reason accounting for the differences between our mouse models and those published by Pedrotti et al. (21) is that deletion of *Suv420h1* in the *Suv420h1* and *Suv420h2* double-KO mouse model may dominate the phenotypes, resulting in increased mitochondrial function and thermogenesis in brown adipocytes, whereas — in our animal model of *Suv420h2* deletion — reduced *Suv420h2* function along with normal or possibly enhanced *Suv420h1* function could collectively lead to impaired brown/beige adipocyte thermogenesis. Future studies using genetic models with gain and loss of functions of individual Suv420h proteins are warranted to carefully dissect the effect of *Suv420hs* on adaptive thermogenesis.

Zhao et al. (56) reported that mice with adipocyte-specific *Suv420h2/*lysine methyltransferase 5C (*Kmt5c*) deletion exerted decreased thermogenic gene expression in WAT and BAT and were prone to diet-induced obesity and associated metabolic disorders. These phenotypes were similar to the phenotypes observed in our H2KO models. Mechanistically, the authors showed that enhanced expression of a negative regulator of brown fat thermogenesis, transformation related protein 53 (*Trp53*) in *Suv420h2/Kmt5c*-KO mice, due to decreased H4K20me3 on its proximal promoter was responsible for the metabolic phenotypes (56). In our current study, we have identified a mechanism, in which *Suv420h2* suppresses the expression of a negative regulator of PGC1α protein translation, *4e-bp1*, by increasing repressive mark H4K20me3 on its promoter, thus promoting brown/beige adipocyte mitochondrial oxidative metabolism and thermogenesis. These complementary studies could significantly enhance our understandings of how *Suv420h1/h2* regulates brown/beige adipocyte thermogenesis and whole body metabolic homeostasis.

In our current study, we observed significant differences in the metabolic phenotypes in our animal models during a cold challenge, whereas the differences diminished in animals challenged with an obesogenic HFD at ambient temperature. It is possible that diet-induced thermogenesis and cold-induced thermogenesis may be triggered by different stimulations. In the context of increased energy needs (cold environment), the purpose of BAT activation is to increase heat production and maintain temperature stability. This is in contrast to a positive energy balance in diet-induced obesity, in which increased heat is not necessary but energy expenditure increases owing to diet-induced thermogenesis, a phenomenon in which excess caloric consumption increases metabolic rate and stimulates BAT thermogenesis (2). Thermogenesis might be stimulated via different mechanisms, depending on whether it is triggered through cold or other factors (57). Additionally, cold and diet can lead to differential gene expression patterns in BAT and WAT (58). Our previous data also show that BAT responded differently in response to a HFD or a cold challenge (59). Thus, it is possible that there are differences in metabolic phenotypes in our animal model during a cold challenge versus a HFD challenge.

We also observed that metabolic differences during a HFD challenge were more evident in animals housed under thermoneutrality compared with the ambient temperature. Mice housed at ambient room temperature have a metabolic rate and food intake around 1.5 times higher than mice housed at thermoneutrality (3). While diet-induced thermogenesis might be primarily dependent on UCP1-dependent brown fat thermogenesis, metabolic rate in response to a cold environment could be influenced by factors other than brown fat adaptive thermogenesis — for example, shivering, skin/fur insulation, and most importantly, adipose tissue response to sympathetic activation (3, 60). These factors could mask the true intrinsic energetic demands in response to a HFD if mice are housed at ambient temperature that presents mild cold stress condition. These different metabolic adaptations may be partly responsible for the differences in metabolic phenotypes observed in our animal models housed at different environmental temperatures. The thermogenic adaptation to diet-induce obesity in an animal model may be partially dependent on the difference in environment temperature.

In summary, we discovered a unique expression pattern of the histone methyltransferase *Suv420h2*, which mirrors the appearance of developmental beige adipocytes. Using genetic models with loss or gain of functions of *Suv420h2*, we demonstrate that *Suv420h2* promotes the development of brown and beige adipocytes postnatally, enhances cold-induced thermogenesis, and prevents diet-induced obesity, possibly through the 4E-BP1/PGC1α axis. We conclude that *Suv420h2* is a key regulator of brown/beige fat thermogenesis, energy metabolism, and diet-induced obesity.

## Methods

### Sex as a biological variant.

Our study examined both male and female mice. However, we found there were sex-dimorphic effects and the phenotypes were more profound in males. Thus, results from male mice are reported.

### Mice.

Mice with whole body H2KO were provided by Gunnar Schotta (Ludwig Maximilian University, Munich, Germany) (22). To generate transgenic mice with adipocyte-specific *Suv420h2* overexpression (AH2Tg), a bacterial artificial chromosome (BAC) containing the mouse adiponectin gene was used, and full-length coding sequence of the mouse *Suv420h2* gene was PCR amplified and inserted into the ATG position at exon 2 of the adiponectin gene in the BAC using homologous recombination. The adiponectin BAC carrying *Suv420h2* was linearized and microinjected into pronuclei of fertilized embryos of C57BL/6J mice at Georgia State University transgenic core facility.

### Metabolic analysis.

Mice were housed in a temperature- and humidity-controlled environment with a 12/12-hour light-dark cycle and had ad libitum access to water and food. H2KO, AH2Tg mice, and their respective littermate controls were fed a regular chow diet (LabDiet, 5001, 13.5% calories from fat) or a HFD (Research Diets, D12492, 60% calorie from fat) for up to 24 weeks. Various metabolic measurements were characterized. Body weight was monitored weekly. Body composition including fat and lean mass was analyzed using a Minispec NMR body composition analyzer (Bruker BioSpin Corporation). Food intake was measured in single-housed animals over 7 consecutive days. Energy expenditure and locomotor activity were assessed using PhenoMaster metabolic cage systems (TSE Systems). Insulin sensitivity was assessed by GTT and ITT, respectively (61, 62). Blood glucose was measured by OneTouch Ultra Glucose meter (LifeScan). At the end of experiments, tissues including BAT and WAT were dissected, weighed, and frozen in liquid nitrogen for further analysis.

### Cold exposure.

H2KO, AH2Tg mice, and their respective littermate controls were subjected to a cold challenge (5°C–6°C) for 7 days. To measure body temperature, some animals were surgically implanted with a temperature transponder (BioMedic Data Systems) in the peritoneal cavity (61, 62). At the end of the experiment, WAT and BAT were dissected, weighed, and frozen for further analysis.

### Cell culture and siRNA knockdown.

Immortalized BAT1 brown adipocyte cells (15, 16) were provided by Patrick Seale (University of Pennsylvania, Philadelphia, Pennsylvania, USA). BAT1 brown adipocytes were grown and differentiated as we described (62). *Suv420h2*, *4e-bp1* targeting siRNA, and nontargeting scramble siRNA were purchased from Dharmacon (Supplemental Table 1). Plasmids containing *Suv420h1* and *Suv420h2* cDNAs were purchased from Open Biosystems (Supplemental Table 1). *Suv420h2*, *4e-bp1* siRNAs, *Suv420h1*, or *Suv420h2* overexpression plasmids were electroporated into BAT1 brown adipocytes using Amaxa Nucleofector II Electroporator (Lonza) with an Amaxa Cell Line Nucleofector Kit L (Lonza) (62). In some experiments, after *Suv420h2* siRNA knockdown, BAT1 cells were further treated with the SUV420H1/H2 inhibitor A196 (5 μM) (Sigma-Aldrich, SML1565) (19) for an additional 24 hours. In other experiments, BAT1 brown adipocytes were treated with both *Suv420h2* and *4e-bp1* siRNAs to knock down both *Suv420h2* and *4e-bp1*. Some cells were also treated with either vehicle (PBS) or the adrenergic agonist norepinephrine (1 μm) for 4 hours.

### qPCR analysis of gene expression.

Total RNA was extracted from fat tissues using Tri Reagent kit (Molecular Research Center) (61, 62). The expression of target genes was measured by qPCR analysis with a TaqMan Universal PCR Master Mix kit (Thermo Fisher Scientific) using an Applied Biosystems QuantStudio 3 real-time PCR system (Thermo Fisher Scientific) (61, 62). The TaqMan primers/probe pairs for the gene expression measurements were either purchased from Applied Biosystems (Thermo Fisher Scientific) (Supplemental Table 2) or commercially synthesized (Applied Biosystems; the sequences are listed in Supplemental Table 3).

### Immunoblotting.

Protein levels of target genes were measured by immunoblotting as we described (61, 62). Briefly, fat tissues were disrupted with a homogenizer in a modified radioimmunoprecipitation assay (RIPA) lysis buffer supplemented with protease and phosphatase inhibitor mixtures (Sigma-Aldrich). After centrifugation (17,000*g* for 30 minutes at 4°C), supernatants were resolved by SDS-PAGE and transferred to nitrocellulose membranes (Bio-Rad), followed by incubating with various primary antibodies and Alexa Fluor 680–conjugated secondary antibodies (Invitrogen). The blots were developed with a LI-COR Imager System (LI-COR Biosciences). The antibody information is listed in Supplemental Table 4.

### IHC.

IHC was conducted as we described (61, 62). Briefly, fat tissues were fixed in neutral formalin, embedded in paraffin, and sliced into 5 μm sections. The tissue slides were used for H&E staining or immunochemical staining with primary and secondary antibodies, which were further developed with an alkaline phosphatase substrate using Vector Red Substrate kit (Vector Laboratories, SK-5100). Histological images were captured using an Olympus DP73 photomicroscope and CellSens software (Olympus). The adipocyte size was measured using ImageJ software (NIH) with Adiposoft plug-in (63). The primary and secondary antibodies are listed in Supplemental Table 4.

### ChIP assays.

ChIP assays were performed with a ChIP assay kit (Upstate BioTechnology) as we described (62). Briefly, fat tissues were fixed and dounce-homogenized for nuclei isolation. The nuclei were used for sonication to shear DNA, followed by immunoprecipitation and elution. The immunoprecipitated DNA was quantitated by real-time PCR using SYBR green. The information for primer sequences was shown in Supplemental Table 5.

### OCR measurement.

Brown adipocyte OCR was measured using a XF 96 Extracellular Flux Analyzer (Agilent) as we described (62). OCR measurement began with a basal respiration recording, followed by addition of other reagents including oligomycin for inhibition of the coupled respiration and FCCP for maximal respiration.

### RNA-Seq analysis.

Total RNA was isolated from iWAT of cold-challenged WT, H2KO, and AH2Tg mice. Equal amounts of RNA from 6 animals/group were pooled and sent to Beijing Genomics Institute (BGI) for RNA-Seq analysis. Clean reads were aligned to the mouse reference genome (University of California Santa Cruz Mouse Genome Browser mm9 Assembly) using SOAPaligner/SOAP2 (64). Differential expression analysis was performed using DESeq2 (65). Differentially expressed genes between groups were defined as log_2_ fold change cutoff threshold of 0.5 and FDR < 0.05. Pathway analysis was performed using the clusterProfiler (66). The RNA-Seq data were also used to predict adipose tissue browning capacity with an online bioinformatic software (https://github.com/PerocchiLab/ProFAT) (23).

### ATAC-Seq analysis.

ATAC-Seq was conducted according to the Omni-ATAC-Seq protocol as described (67). Briefly, BAT tissues were dounce-homogenized, filtered, and centrifuged in iodixanol solution at 3,000*g* for 20 minutes at 4°C. The middle layer containing nuclei was collected, washed, and then centrifuged at 500*g* for 1 minute at 4°C to obtain nuclei. The nuclei were treated with Nextera Tn5 transposase (Illumina) for the transposition reaction, followed by DNA purification and PCR amplification with NEBNext 2X MasterMix (New England BioLabs) and Nextera Index primers (Illumina). The ATAC libraries were further size purified and sent to Novogene for sequencing. The ATAC-Seq analysis was performed on the Galaxy server as described (67). Briefly, the adaptor-trimmed sequencing reads were mapped to the mm10 mouse reference genome using Bowtie2 (68). After removing PCR duplicates and reads mapped to ENCODE blacklist regions, the ATAC-Seq peaks were called using MACS2 (69). Finally, differential ATAC-Seq peaks between groups were identified using DiffBind. The integration of RNA-Seq and ATAC-Seq data was carried out in R, and heatmaps were generated using the ComplexHeatmap package (70).

### Statistics.

Data were expressed as mean ± SEM. Statistical tests were performed using SPSS software (version 16.0, SPSS Inc). Differences between groups were analyzed for statistical significance by Student’s *t* test, 1-way or 2-way ANOVA, or 2-way ANOVA with repeated measures as appropriate. Statistical significance was accepted at *P* < 0.05.

### Study approval.

All animal procedures conducted in the study were approved by the IACUC at Georgia State University.

### Data availability.

The RNA-Seq and ATAC-Seq data have been deposited to Gene Expression Omnibus (GEO) database with the accession nos. GSE244457 and GSE245509, respectively. Values for graphs in the figures and supplemental figures are provided in the Supporting Data Values file.

## Author contributions

XC performed most of the experiments and analyzed the data; QC, FL, JJ, ZL, and XY assisted with various experiments; HDS performed bioinformatic analysis of RNA-Seq and ATAC-Seq data; GS and LY contributed to conceptual and technical inputs and review/edits on manuscript; and HS and BZ conceived and designed the study and wrote the manuscript.

## Supplementary Material

Supplemental data

Unedited blot and gel images

Supporting data values

## Version 1 05/07/2024

In-Press Preview

## Version 2 06/10/2024

Electronic publication
