# Supplementary material for: The histone methyltransferase SUV420H2 regulates brown and beige adipocyte thermogenesis
Source: JCI Insight. 2024 May 7;9(11):e164771. doi: 10.1172/jci.insight.164771 (PMC11382888; doi:10.1172/jci.insight.164771)
Supplement: Supplemental data [file jciinsight-9-164771-s142.pdf]

Supplemental Figures, Figure Legends and Tables,

The Histone Methyltransferase SUV420H2 Regulates Brown and Beige Adipocyte Thermogenesis

Xin Cui[1], Qiang Cao[1], Fenfen Li[1], Jia Jing[1], Zhixue Liu[1], Xiaosong Yang[1,2], Gary J. Schwartz[3], Liqing Yu[4], Huidong Shi[5,6], Hang Shi[1*], Bingzhong Xue[1*]

[1]Department of Biology, Georgia State University, Atlanta, GA 30303, USA.

[2]Present address: Hubei Key Laboratory of Diabetes and Angiopathy, Hubei University of Science and Technology, Xianning, 437100, China

[3]Department of Medicine, Albert Einstein College of Medicine, Bronx, NY 10461

[4]Department of Medicine, University of Maryland School of Medicine, Baltimore, MD, USA.

[5]Georgia Cancer Center, and [6]Department of Biochemistry and Molecular Biology, Medical College of Georgia, Augusta University, Augusta, GA 30912, USA

* Correspondence should be addressed to:

Bingzhong Xue, Department of Biology, Georgia State University, Atlanta, GA 30303, USA. Contact: 404-413-5747, bxue@gsu.edu

Hang Shi, Department of Biology, Georgia State University, Atlanta, GA 30303, USA. Contact: 404-413-5799, hshi3@gsu.edu.

Supplemental Figure 1.

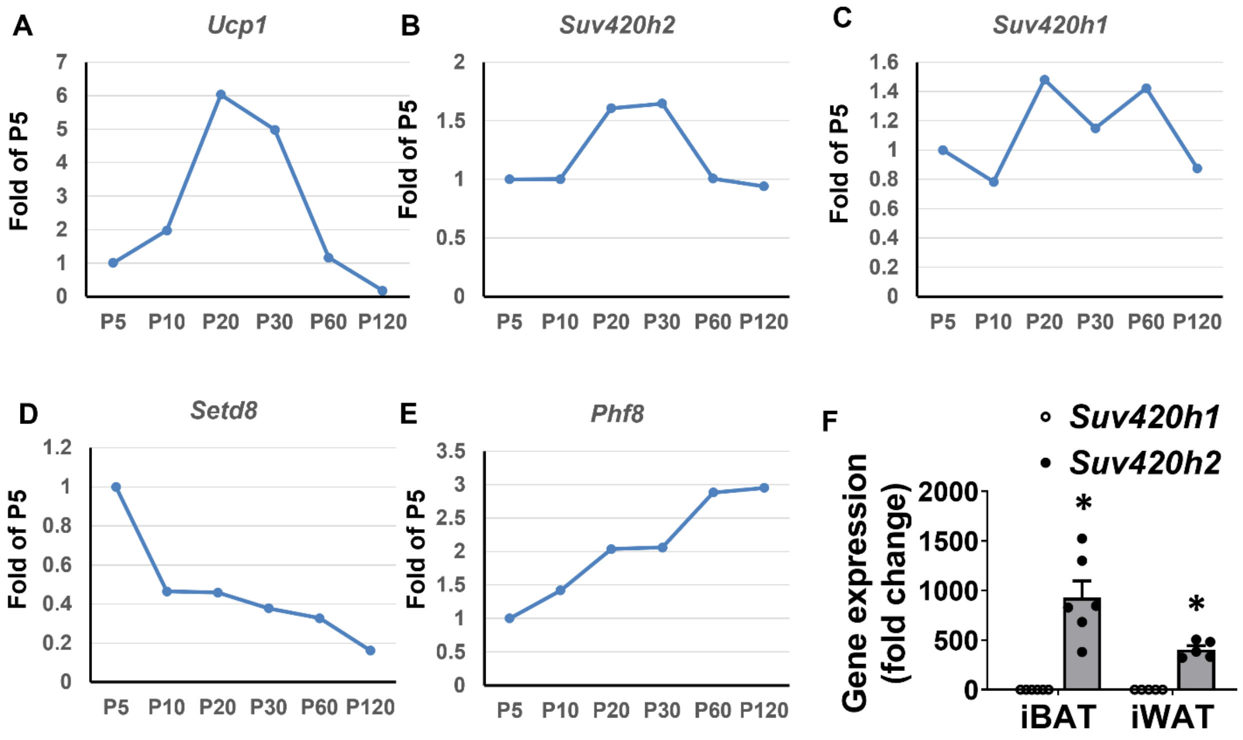

Supplemental Figure 1. Gene expression profiles in iWAT of mice from postnatal day 5 (P5) to postnatal day 120 (P120). (A) Uncoupling protein 1 (*Ucp1*); (B) Suppressor of variegation 4-20 homolog 2 (Drosophila) (*Suv420h2*); (C) *Suv420h1*; (D) SET domain containing protein 8 (*Setd8*); and (E) PHD finger protein 8 (*Phf8*). (F). The expression of *Suv420h1* and *Suv420h2* in iBAT and iWAT. For (A)-(E), samples for each time points were pooled from 4 animals. For (F), data are expressed as mean ± SEM, n=5-6/group. *p<0.05 vs. Suv420h1 by unpaired two-tailed Student's t-test.

Supplemental Figure 2.

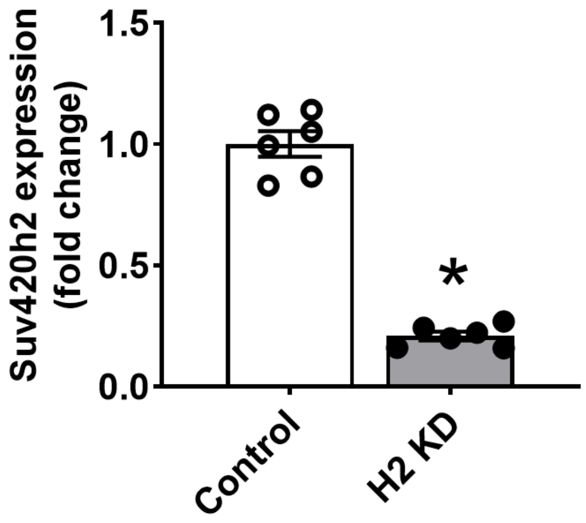

Supplemental Figure 2. The expression of *Suv420h2* in BAT1 brown adipocytes after siRNA knockdown of *Suv420h2*. All data are expressed as mean ± SEM, n=6/group. *p<0.05 vs. Control with scrambled siRNA by unpaired two-tailed Student's t-test.

Supplemental Figure 3.

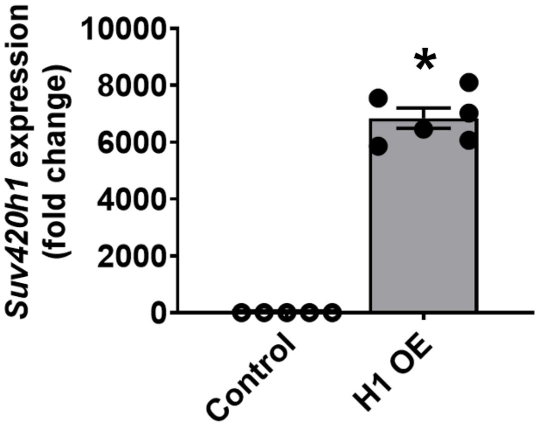

Supplemental Figure 3. The expression of *Suv420h1* in BAT1 brown adipocytes after overexpression of *Suv420h1*. All data are expressed as mean ± SEM, n=5-6/group. *p<0.05 vs. Control with scrambled siRNA by unpaired two-tailed Student's t-test.

Supplemental Figure 4.

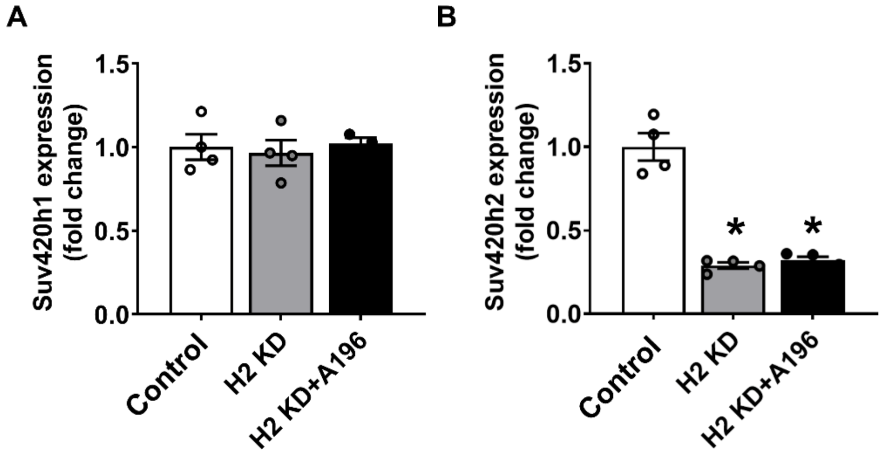

Supplemental Figure 4. The expression of *Suv420h1* (A) and *Suv420h2* (B) in BAT1 brown adipocytes after siRNA knockdown of *Suv420h2* with or without further treatment of A196. Four-day differentiated BAT1 cells were treated with either scramble or *Suv420h2* siRNA via electroporation as we described in the Materials and Methods. On day 6 of differentiation, cells were further treated with vehicle (dimethyl sulfoxide (DMSO)) or the SUV420H1/H2 inhibitor A196 (5µM) for 24 hours. Cells were harvested for RNA analysis. All data are expressed as mean ± SEM, n=3-4/group. *p<0.05 vs. Control by one-way ANOVA followed by Tukey's multiple comparisons test. Control: Scramble siRNA+DMSO; H2KD: Suv420h2 siRNA+DMSO; H2KD+A196: Suv420h2 siRNA+A196.

Supplemental Figure 5.

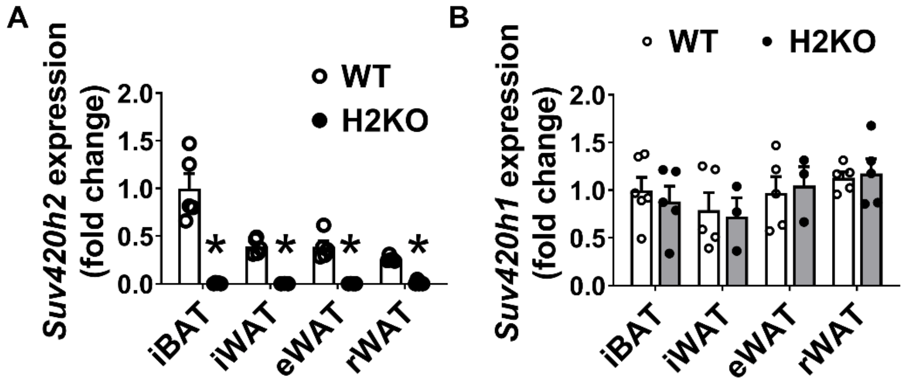

Supplemental Figure 5. The expression of *Suv420h2* (A) and *Suv420h1* (B) in adipose tissues of 2-month-old male WT and H2KO mice. All data are expressed as mean ± SEM, n=3-5/group. *p<0.05 vs. WT by unpaired two-tailed Student's t-test.

Supplemental Figure 6.

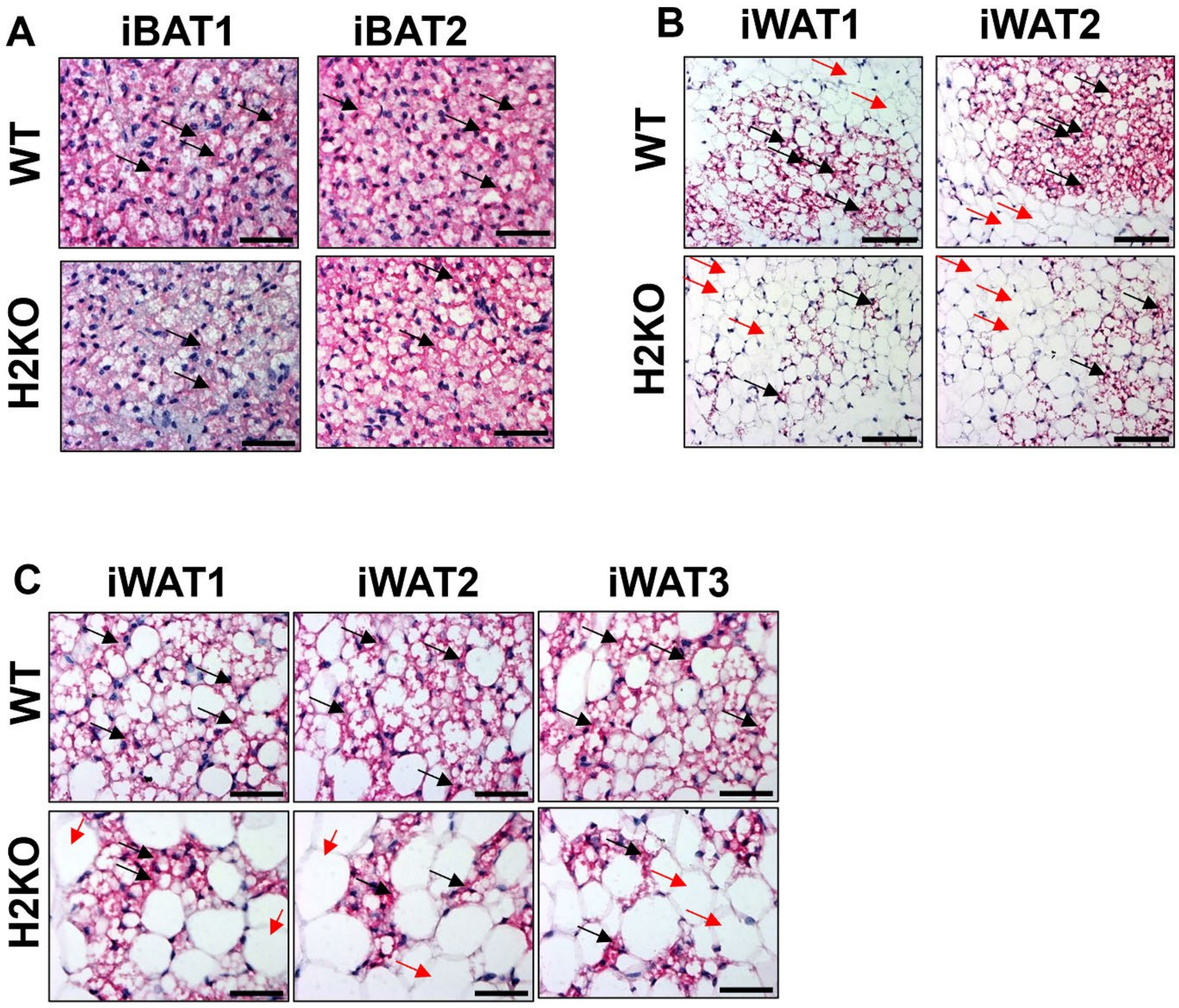

Supplemental Figure 6. UCP1-immunostaining in iBAT (A, Scale bar=70µm) and iWAT (B-C: B, scale bar=140µm; C, higher magnification, scale bar=70µm) of 20-day-old H2KO and WT mice housed at room temperature. UCP1-positive multilocular brown/beige adipocytes were shown in dark purplish red color, and were indicated with black arrows; and UCP1-negative unilocular white adipocytes were shown in light color, and were indicated with red arrows

Supplemental Figure 7.

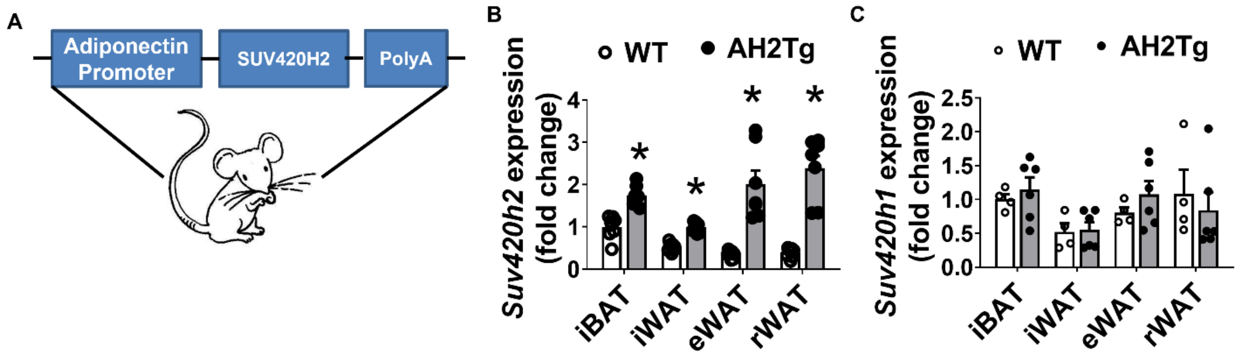

Supplemental Figure 7. Generation of transgenic mouse models with adipocyte-specific overexpression of Suv420h2. (A) Schematic illustration of the transgene construct. (B)-(C) The expression of *Suv420h2* (B) and *Suv420h1* (C) in adipose tissues of 2-month-old WT and AH2Tg mice. All data are expressed as mean ± SEM, n=7-8/group in (B) and n=4-6/group in (C). *p<0.05 vs. WT by unpaired two-tailed Student's t-test.

Supplemental Figure 8.

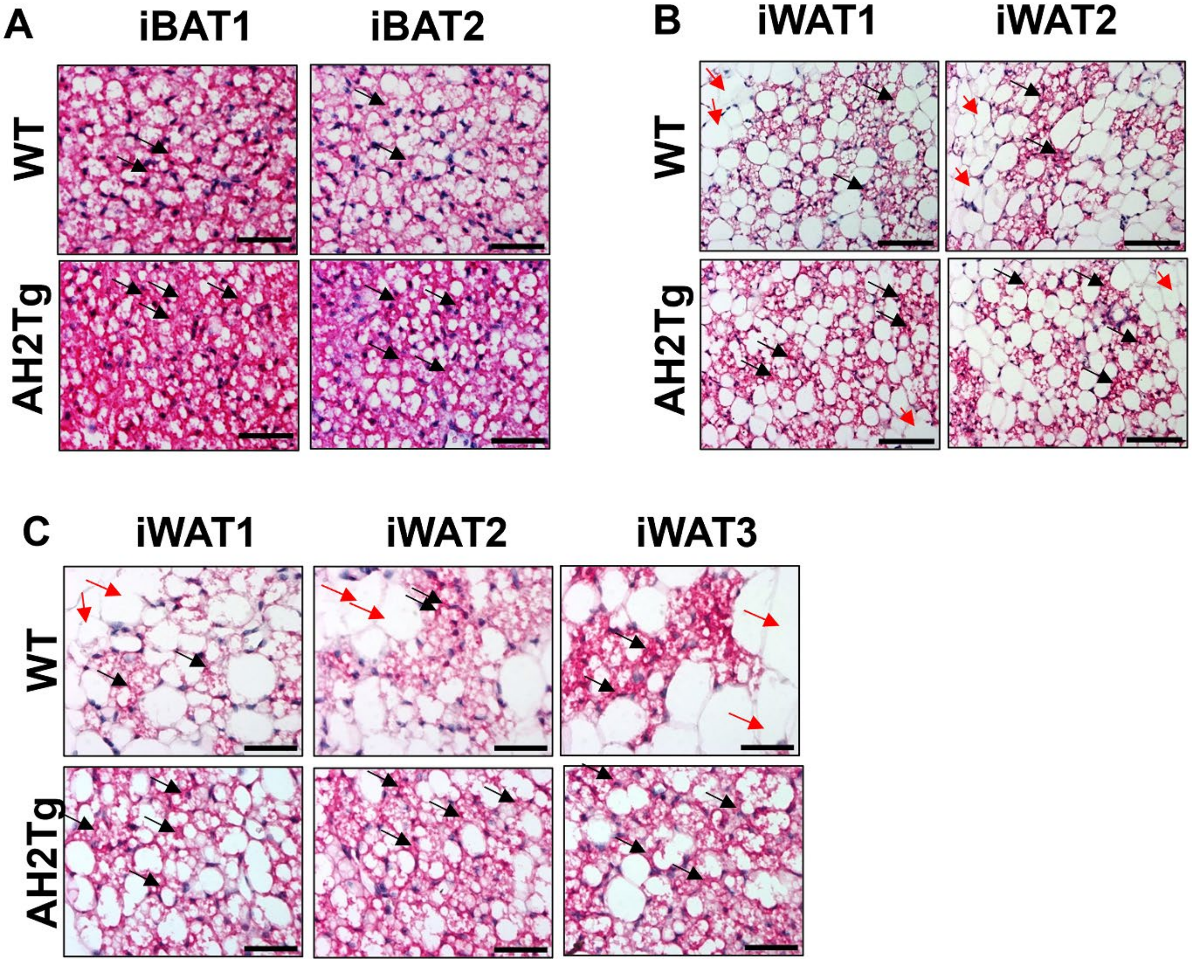

Supplemental Figure 8 UCP1-immunostaining in iBAT (A, Scale bar=70μm) and iWAT (B-C: B, scale bar=140μm; C, higher magnification, scale bar=70μm) of 20-day-old AH2Tg and WT mice housed at room temperature. UCP1-positive multilocular brown/beige adipocytes were shown in dark purplish-red color, and were indicated with black arrows; and UCP1-negative unilocular white adipocytes were shown in light color, and were indicated with red arrows.

Supplemental Figure 9.

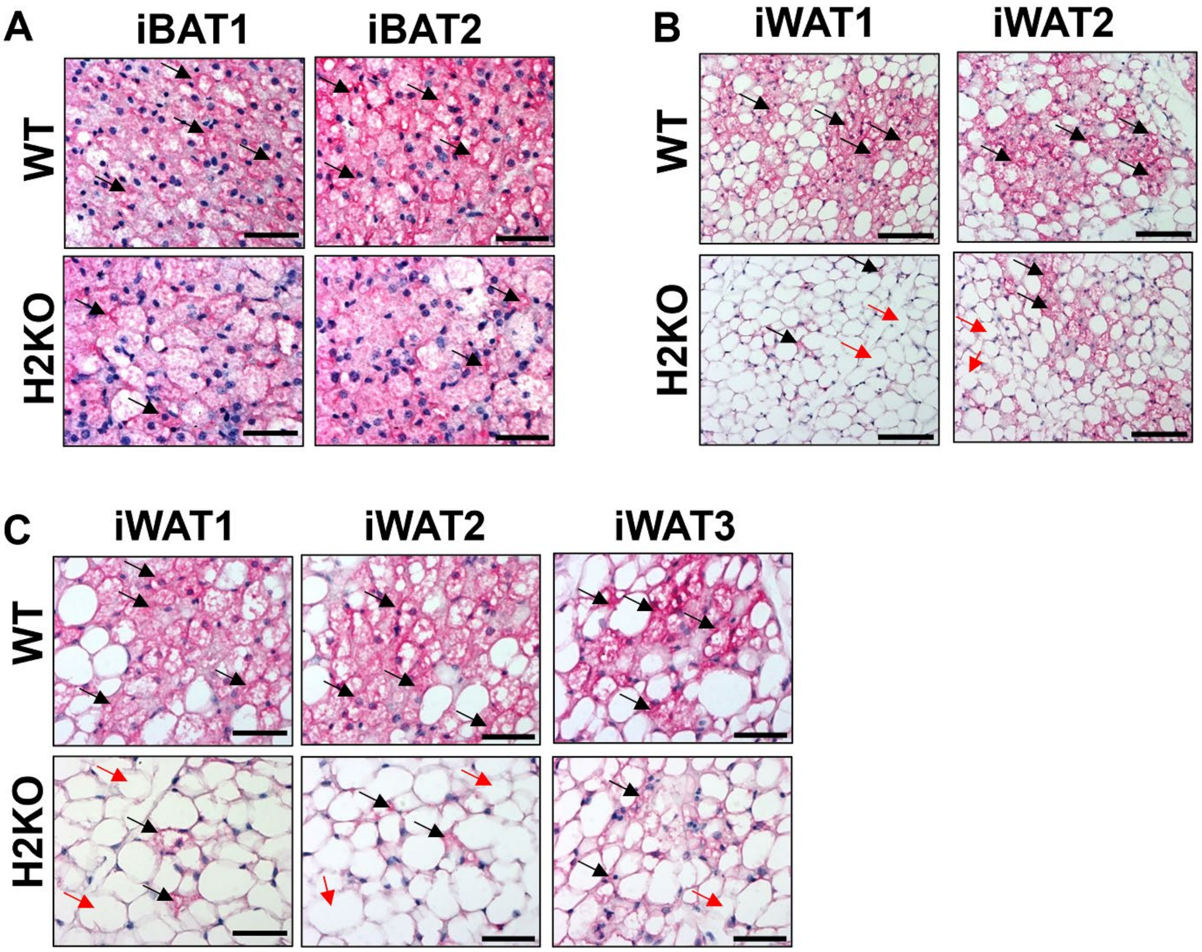

Supplemental Figure 9. UCP1-immunostaining in iBAT (A, Scale bar=70μm) and iWAT (B-C: B, scale bar=140μm; C, higher magnification, scale bar=70μm) of 2-3-month-old H2KO and WT mice after a 7-day 5°C cold challenge. UCP1-positive multilocular brown/beige adipocytes were shown in dark purplish-red color, and were indicated with black arrows; and UCP1-negative unilocular white adipocytes were shown in light color, and were indicated with red arrows.

Supplemental Figure 10.

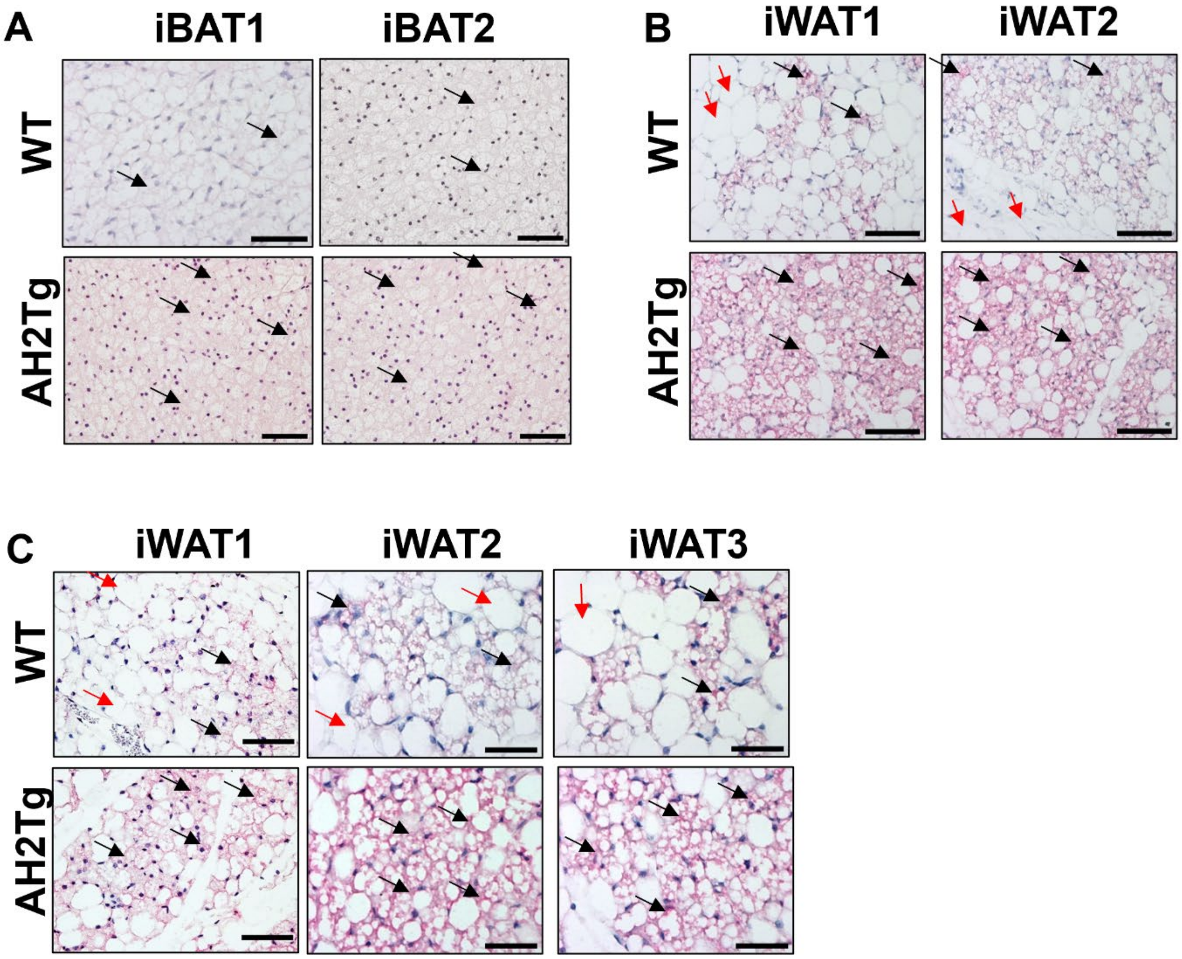

Supplemental Figure 10. UCP1-immunostaining in iBAT (A, Scale bar=70µm) and iWAT (B-C: B, scale bar=140µm; C, higher magnification, scale bar=70µm) of 2-3-month-old AH2Tg and WT mice after a 7-day 5°C cold challenge. UCP1-positive multilocular brown/beige adipocytes were shown in dark purplish-red color, and were indicated with black arrows; and UCP1-negative unilocular white adipocytes were shown in very light color, and were indicated with red arrows.

Supplemental Figure 11.

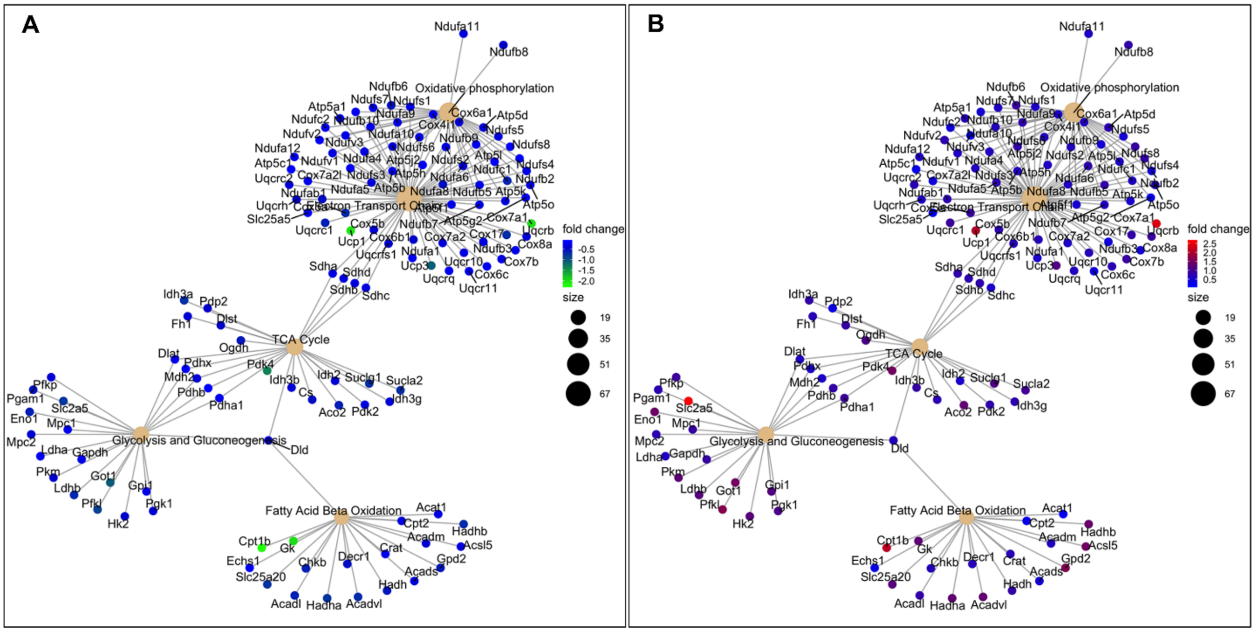

Supplemental Figure 11. Pathway analysis revealed genes and pathways involved in Electron Transport Chain, TCA cycle, Glycolysis and Gluconeogenesis, and fatty acid beta-oxidation were downregulated in iWAT of H2KO mice compared to that of WT mice (A), but reciprocally upregulated in iBAT of AH2Tg mice compared to that of WT mice (B) after a 7-day cold challenge.

Supplemental Figure 12.

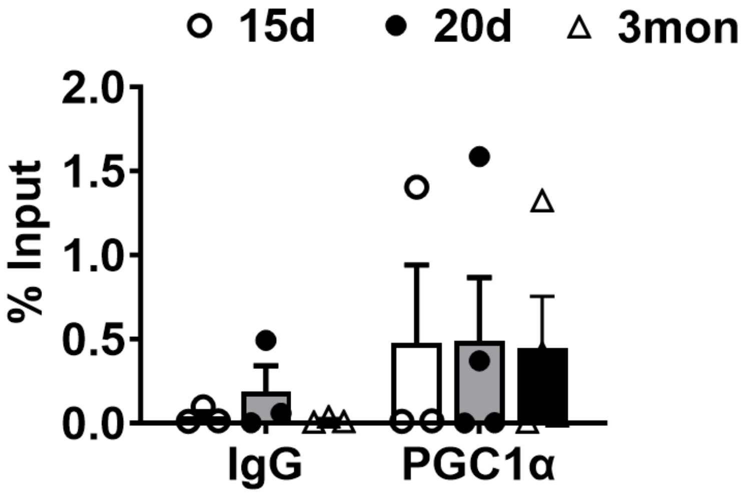

Supplemental Figure 12. H4K20me3 levels at the *Pgc1α* promoter in iWAT of male C57BL/6J mice during postnatal development at ages of 15 days (15d), 20 days (20d) and 3 months (3mon). All data are expressed as mean ± SEM, n=3-4/group.

Supplemental Figure 13.

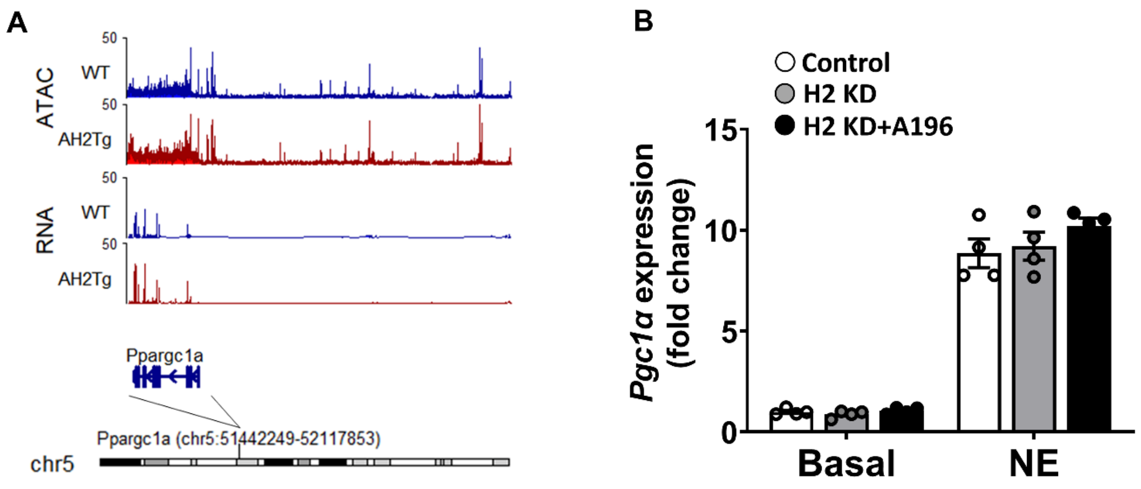

Supplemental Figure 13. (A) ATAC-seq analysis of chromatin accessibility and RNAseq peak data at *Pgc1α* gene locus in AH2Tg and WT mice after a 7-day cold exposure. (B) *Pgc1α* expression in BAT1 brown adipocytes treated with *Suv420h2* knockdown with or without further A196 treatment. Briefly, 4-day differentiated BAT1 cells were treated with either scramble or *Suv420h2* siRNA via electroporation. On day 6 of differentiation, cells were further treated with either DMSO or the SUV420H1/H2 inhibitor A196 (5µM) for 24 hours. Before harvesting, cells were further treated with either PBS or NE (1µm) for 4 hours, n=4/group. Control: Scramble siRNA+DMSO; H2KD: *Suv420h2* siRNA+DMSO; H2KD+A196: *Suv420h2* siRNA+A196. All data are expressed as mean ± SEM.

Supplemental Figure 14.

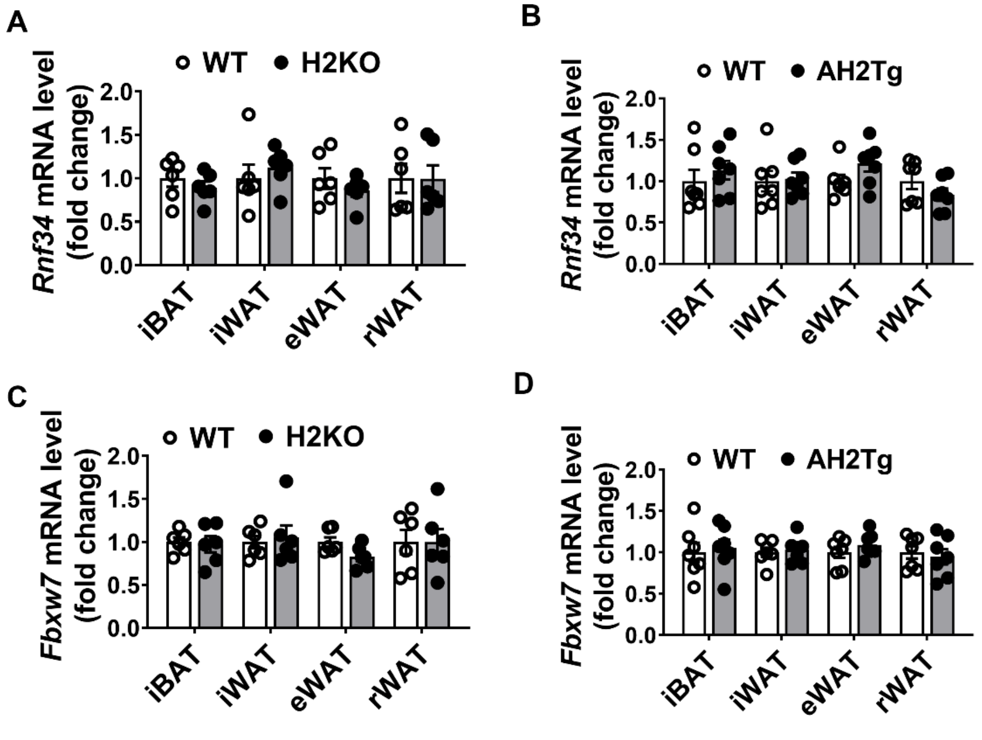

Supplemental Figure 14. (A)-(B) Expression of ring finger protein 34 (*Rnf34*) in adipose tissues of H2KO (A) and AH2Tg (B) mice after a 7-day cold challenge. (C)-(D) Expression of F-box and WD-40 domain protein 7 (*Fbxw7*) in adipose tissues of H2KO (C) and AH2Tg (D) mice after a 7-day cold challenge. All data are expressed as mean ± SEM, n=6-7/group.

Supplemental Figure 15.

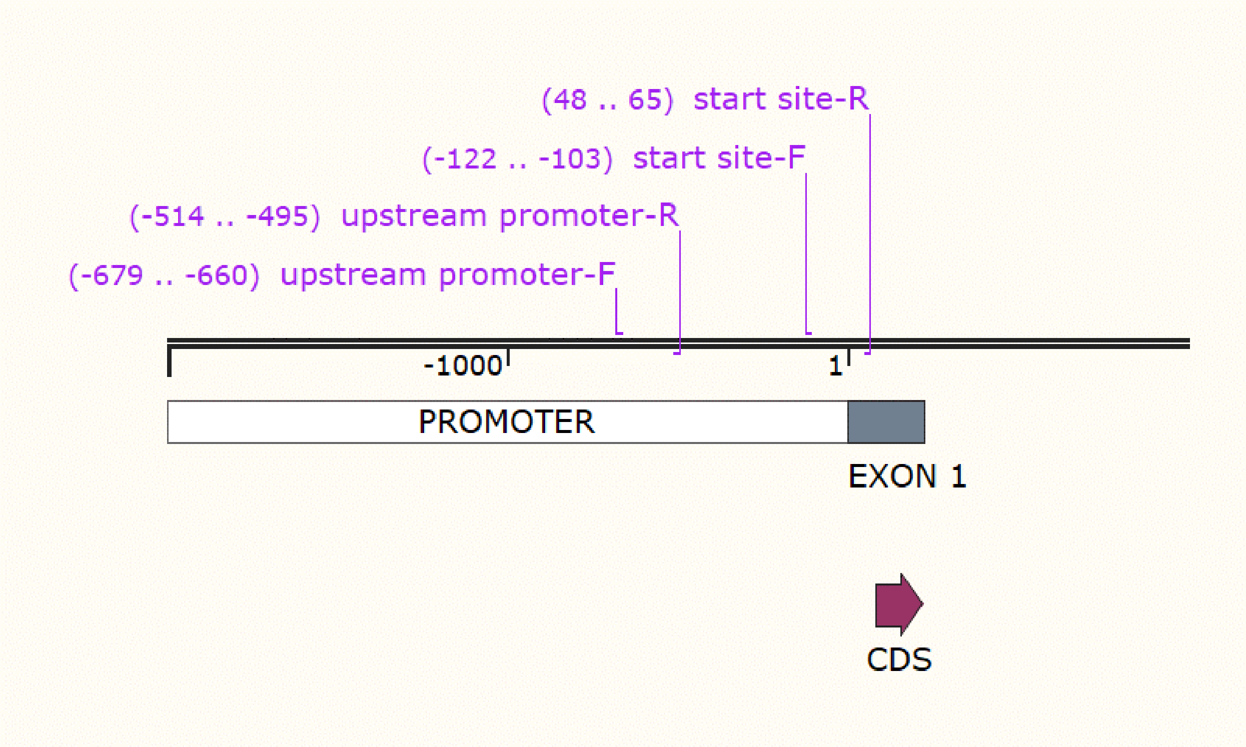

Supplemental Figure 15. Schematic illustration of *4e-bp1* promoter region and positions of primers used in ChIP assay.

Supplemental Figure 16.

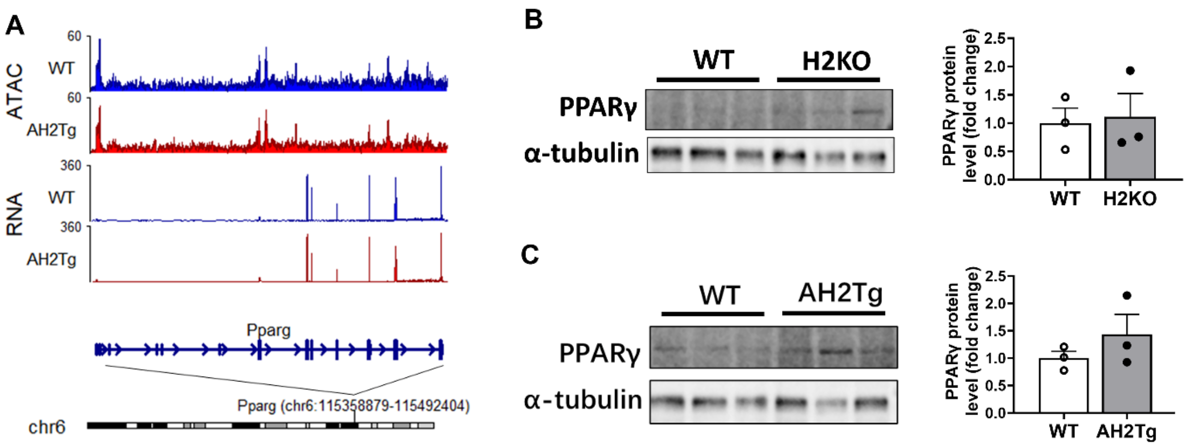

Supplemental Figure 16. (A) ATAC-seq analysis of chromatin accessibility and RNAseq peak data at *Pparg* gene locus in AH2Tg and WT mice after the 7-day cold exposure. (B-C) PPARγ protein levels in iWAT of WT and H2KO mice (B) or WT and AH2Tg mice (C) after 7-day cold exposure. All data are expressed as mean ± SEM, n=3/group.

Supplemental Figure 17.

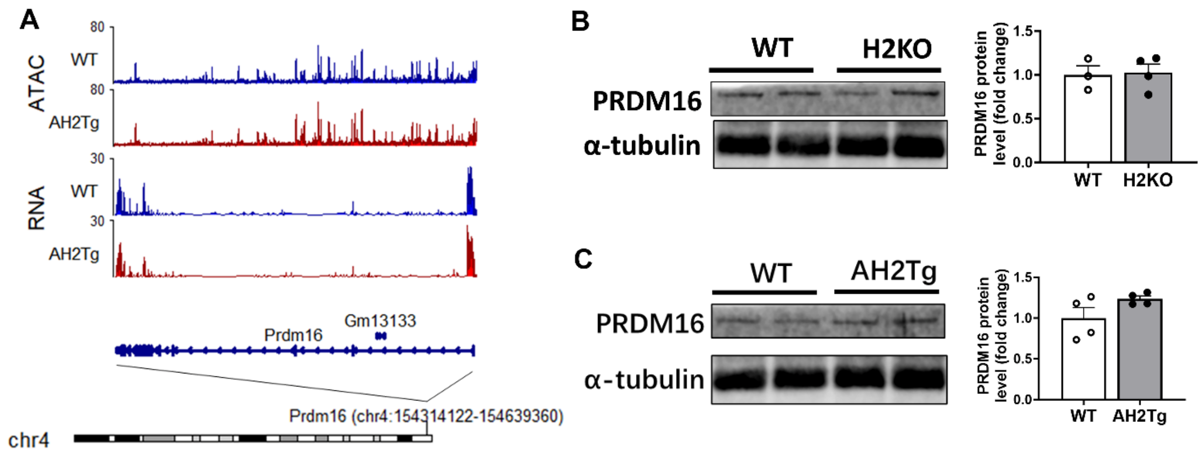

Supplemental Figure 17. (A) ATAC-seq analysis of chromatin accessibility and RNAseq peak data at *Prdm16* gene locus in AH2Tg and WT mice after 7-day cold exposure. (B-C) PRDM16 protein levels in iWAT of WT and H2KO mice (B) or WT and AH2Tg mice (C) after 7-day cold exposure. All data are expressed as mean ± SEM, n=3-4/group.

Supplemental Figure 18.

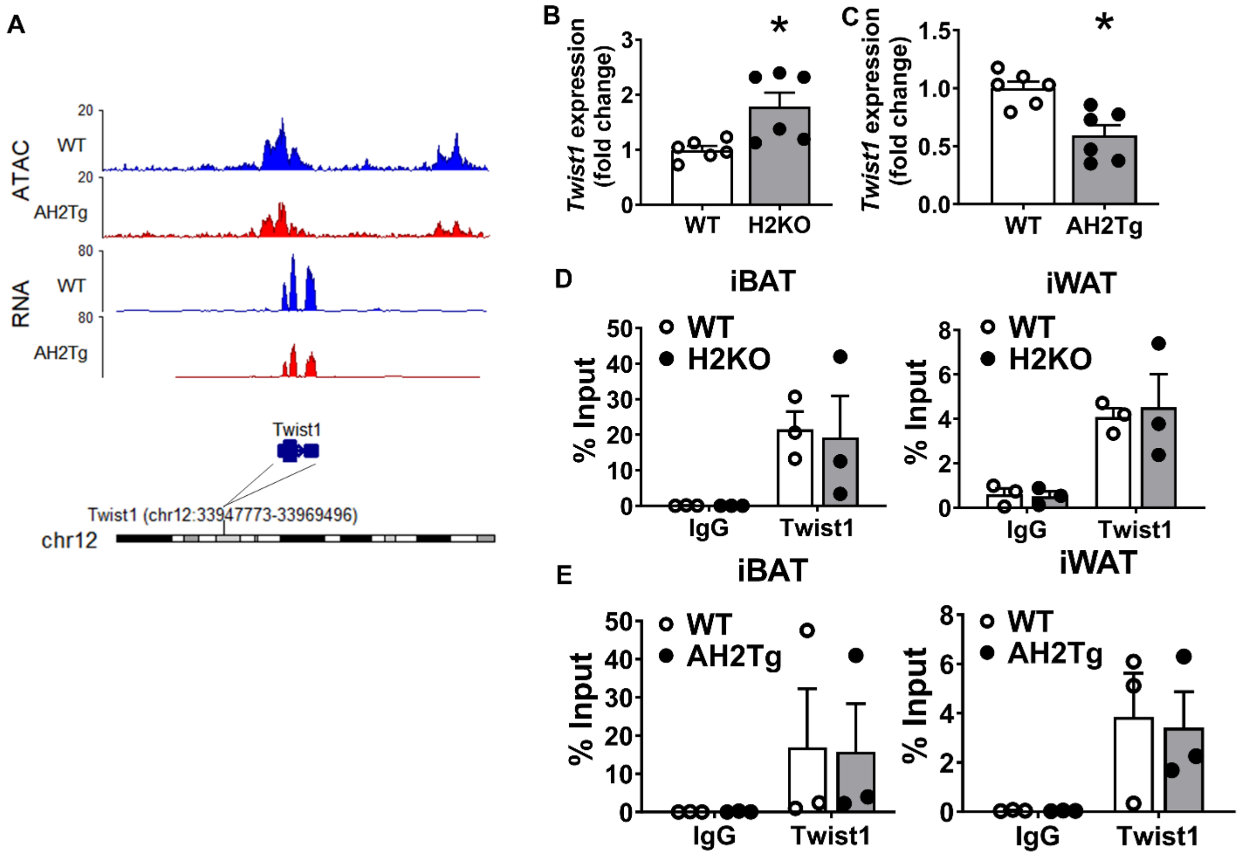

Supplemental Figure 18. (A) ATAC-seq analysis of chromatin accessibility and RNAseq peak data at *Twist1* gene locus in AH2Tg and WT mice after the 7-day cold exposure. (B-C) *Twist1* mRNA expression in iWAT of WT and H2KO mice (B) or WT and AH2Tg mice (C) after 7-day cold exposure. (D-E) H4K20me3 levels at *Twist1* promoter as analyzed by ChIP assay in iBAT and iWAT of WT and H2KO mice (D) or iBAT and iWAT of WT and AH2Tg mice (E) after 7-day cold exposure. All data are expressed as mean ± SEM, n=6/group in (B-C), and n=3/group in (D-E). *p<0.05 by unpaired two-tailed Student's t-test in (B)-(C).

Supplemental Figure 19.

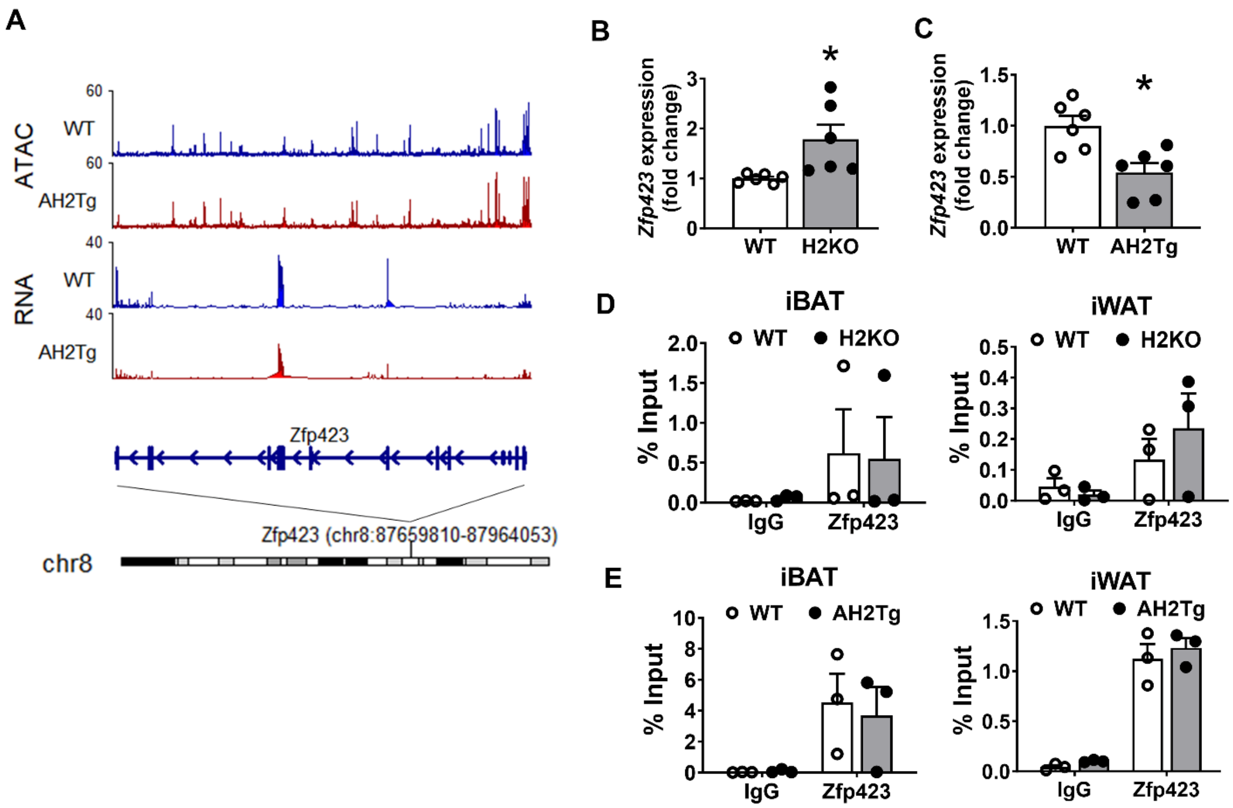

Supplemental Figure 19. (A) ATAC-seq analysis of chromatin accessibility and RNAseq peak data at *Zfp423* gene locus in AH2Tg and WT mice after the 7-day cold exposure. (B-C) *Zfp423* mRNA expression in iWAT of WT and H2KO mice (B) or WT and AH2Tg mice (C) after 7-day cold exposure. (D-E) H4K20me3 levels at *Zfp423* promoter as analyzed by ChIP assay in iBAT and iWAT of WT and H2KO mice (D) or iBAT and iWAT of WT and AH2Tg mice (E) after 7-day cold exposure. All data are expressed as mean ± SEM, n=6/group in (B-C) and n=3/group in (D-E). *p<0.05 by unpaired two-tailed Student's t-test in (B)-(C).

Supplemental Figure 20.

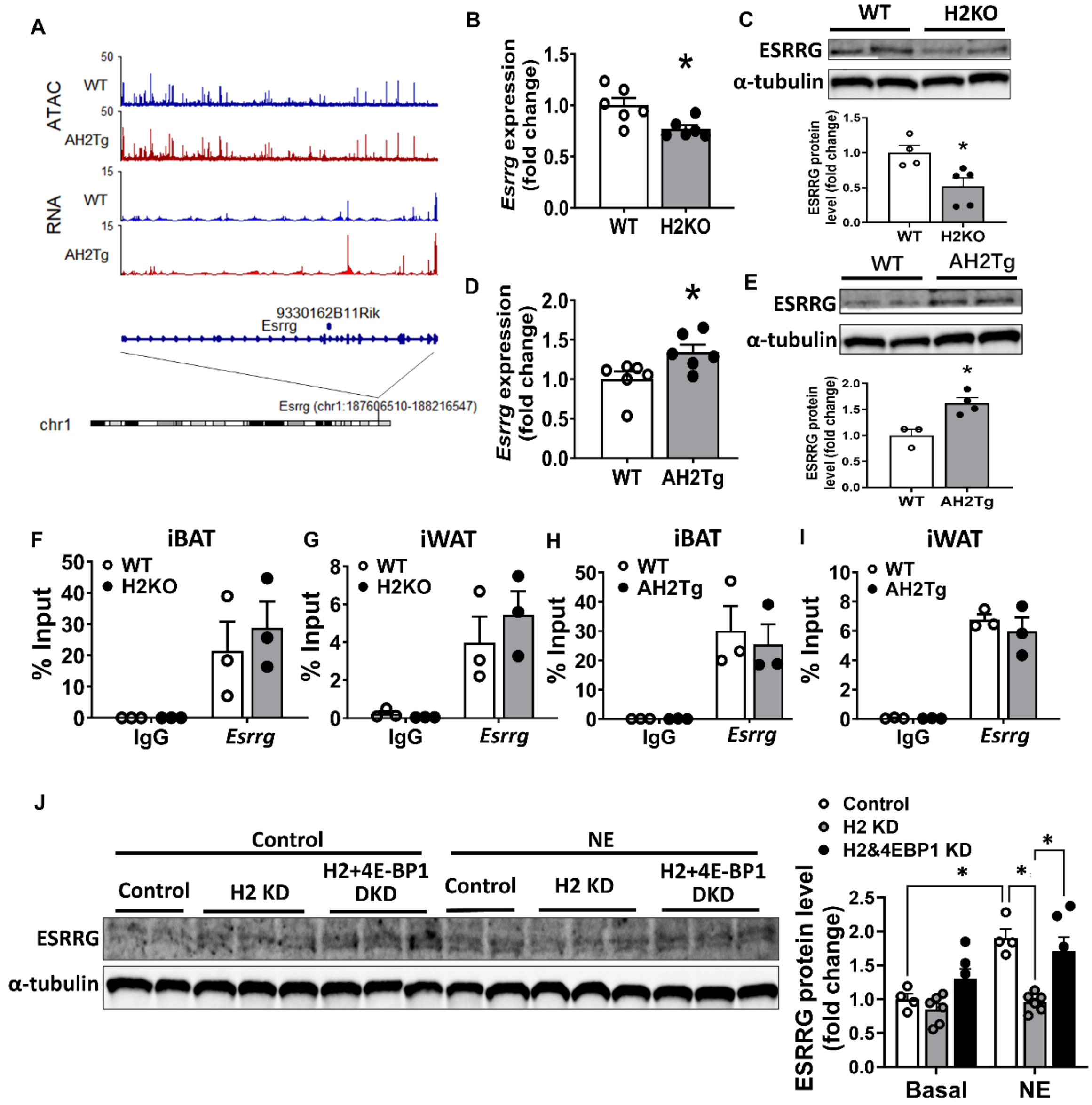

Supplemental Figure 20. (A) ATAC-seq analysis of chromatin accessibility and RNAseq peak data at *Esrrg* gene locus in AH2Tg and WT mice after the 7-day cold exposure. (B-C) *Esrrg* mRNA (B) and protein (C) levels in iWAT of WT and H2KO mice after the 7-day cold exposure. (D-E) *Esrrg* mRNA (D) and protein (E) levels in iWAT of WT and AH2Tg mice after the 7-day cold exposure. (F-G) H4K20me3 levels at *Esrrg* promoter as analyzed by ChIP assay in iBAT and iWAT of WT and H2KO mice (F) or iBAT and iWAT of WT and AH2Tg mice (G) after 7-day cold exposure. (J) Basal and NE-induced ESRRG protein levels in BAT1 brown adipocytes treated with either *Suv420h2* or combined *Suv420h2/4e-bp1* knockdown. All data are expressed as mean ± SEM, n=3-6/group in (B-E), n=3/group in (F-I), and n=4-6/group in (J). *p<0.05 as analyzed by unpaired two-tailed Student's t-test in (B-E) or two-way ANOVA followed by Tukey's multiple comparisons test in (J).

Supplemental Figure 21.

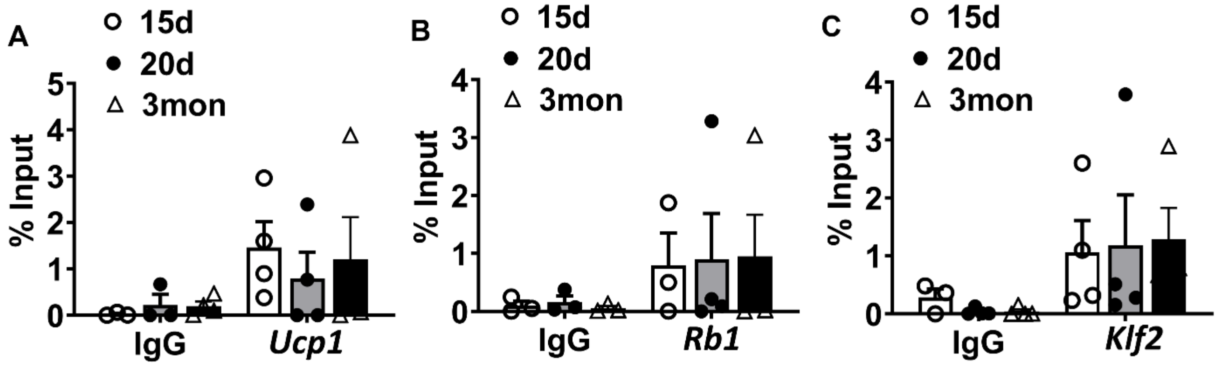

Supplemental Figure 21. H4K20me3 levels at promoters of *Ucp1* (A), *Rb1* (B) and *Klf2* (C) in iWAT of male C57BL/6J mice during postnatal development at ages of 15 days (15d), 20 days (20d) and 3 months (3mon). All data are expressed as mean ± SEM, n=3-4/group.

Supplemental Figure 22.

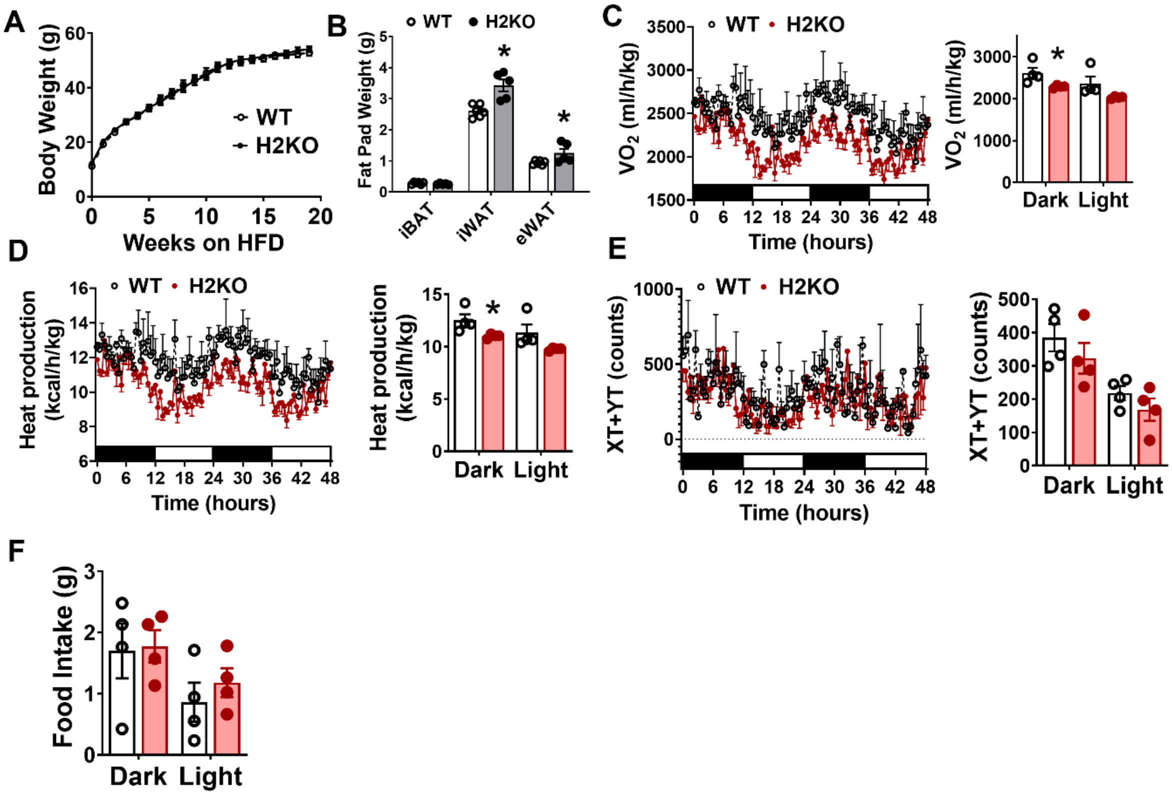

Supplemental Figure 22. Metabolic phenotyping of (A) Body weight (B) Fat pad weight (C) Oxygen consumption (D) Heat production (E) Locomotor activity and (F) Food intake in H2KO and their WT littermate control mice fed a HFD when housed at room temperature. All data are expressed as mean ± SEM, n=4-6/group. *p<0.05 by unpaired two-tailed Student's t-test.

Supplemental Figure 23.

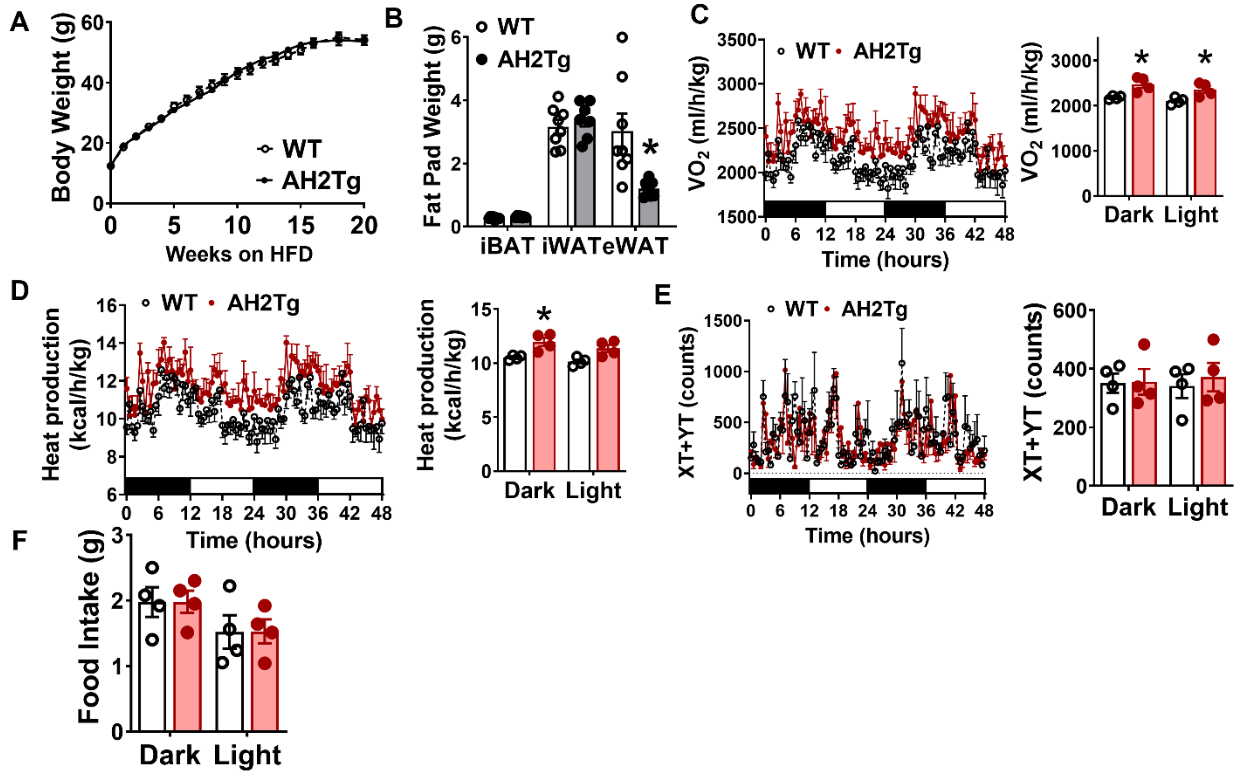

Supplemental Figure 23. Metabolic phenotyping of (A) Body weight (B) Fat pad weight (C) Oxygen consumption (D) Heat production (E) Locomotor activity and (F) Food intake in AH2Tg and their WT littermate control mice fed a HFD when housed at room temperature. All data are expressed as mean ± SEM, n=4-9/group. *p<0.05 by unpaired two-tailed Student's t-test.

Supplemental Table 1. SiRNAs and cDNAs

Products	Company	Catalog #
Suv420h2 siRNA	Dharmacon	L-052050-00-0005
4e-bp1 siRNA	Dharmacon	L-058681-01-0005
Scramble siRNA	Dharmacon	D-001810-01-05
Suv420h2 cDNA	Open Biosystems	MMM1013-202769645
Suv420h1 cDNA	Open Biosystems	MMM4769-202765015

Supplemental Table 2. Primer/probe sets for gene expression analysis

Gene symbol	Company	Catalog #
4e-bp1	ABI	Mm04207378_g1
Acot2	ABI	Mm01622461_s1
Acox1	ABI	Mm01246834_m1
Cidea	ABI	Mm00432554_m1
Cox1	ABI	Mm04225243_g1
Cpt1b	ABI	Mm00487191_g1
Dio2	ABI	Mm0051664_m1
Elovl3	ABI	Mm01194165_g1
Esrrg	ABI	Mm01314576_m1
Eva1	ABI	Mm00468397_m1
Fbxw7	ABI	Mm00504452_m1
Otop1	ABI	Mm00554705_m1
Phf8	ABI	Mm00623340_m1
Pgc1α	ABI	Mm01208835_m1
Pparα	ABI	Mm00440939_m1
Pparγ	ABI	Mm00440945_m1
Prdm16	ABI	Mm00712556_m1
Rnf34	ABI	Mm00504205_m1
Setd8	ABI	Mm01201497_m1
Suv420h1	ABI	Mm00628157_m1
Suv420h2	ABI	Mm00525366_m1
Twist1	ABI	Mm00442036_m1
Zfp423	ABI	Mm00473699_m1

Table 3. Primer/probe sequences for gene expression analysis

Gene	Primer (Forward: 5'-3')	Primer (Reverse: 5'-3')	Probe (5'-3')
**Cyclophilin (*Ppib*)**	GGTGGAGAGCACCAAGACAGA	GCCGGAGTCGACAATGATG	ATCCTTCAGTGGCTTGTCCCGGCT
Ucp1	CACCTTCCCGCTGGACACT	CCCTAGGACACCTTTATACCTAATGG	AGCCTGGCCTTCACCTTGGATCTGA

Supplemental Table 4. Antibodies used in Immunoblotting (WB), immunohistochemistry (IHC) and ChIP-qPCR

Antibody	Company	Catalog #	Application
**4E-BP1**	CST	9452S	WB
**ESRRG**	Invitrogen	PA5-27755	WB
**H4K20me1**	Abcam	ab9051	WB
**H4K20me2**	Millipore	39173	WB
**H4K20me3**	Abcam	ab9053	WB/ChIP
**Mitochondrial total OXPHOS protein antibody set**	Abcam	110413	WB
**PGC1α**	Millipore	ST1202	WB
**PPARγ**	Santa Cruz Biotechnology	sc-7273	WB
**PRDM16**	Sigma	SAB2900806	WB
**α-Tubulin**	Santa Cruz Biotechnology	sc-53646	WB
**UCP1**	Abcam	ab23841	WB
**UCP1**	Abcam	Ab10983	IHC
**Biotin-SP (long spacer) AffiniPure Donkey Anti-Rabbit IgG (H+L)**	Jackson ImmunoResearch	711-065-152	IHC
**Goat anti-Mouse IgG (H+L) Highly Cross-Adsorbed Secondary Antibody, Alexa Fluor 680**	Invitrogen	A21058	WB
**Goat anti-Rabbit IgG (H+L) Highly Cross-Adsorbed Secondary Antibody, Alexa Fluor 680**	Invitrogen	A21109	WB

Table 5. Primer/probe sequences for ChIP assay.

Gene	Primer (Forward: 5'-3')	Primer (Reverse: 5'-3')
***4e-bp1* upstream promoter**	TCACGGTCGGGATTATAGGC	TACTGTGGGCTGGGAGGATT
***4e-bp1* start site**	TTCCTGCACTGGAGAGGACA	CACGACCCTCCAGCAACC
Esrrg	AAGAGGTCGCTCTCACTGGA	GACTCGGGGTTCACTTGAGG
Klf2	AAATTTAGGCTGAGCCCGGAG	CAAAGGACGGCAAGATAGGC
Pgc1α	CAAAGCTGGCTTCAGTCACA	AAAAGTAGGCTGGGCTGTCA
Rb1	TACTTGGGTTCGAGTCCTCTGCCAG	AGTTGGCCGTGTTCATGCG
Ucp1	CCCACTAGCAGCTCTTTGGA	CTGTGGAGCAGCTCAAAGGT
Twist1	GAAAAGTCCCTCCTCCCCAC	GTGGACTTGGCGGCTCTTAT
Zfp423	AAAGTTTCCGAGAGGCAGGA	TGCTTCCGCCTGGACAT
