# Supplementary material for: The histone methyltransferase SUV420H2 regulates brown and beige adipocyte thermogenesis
Source: JCI Insight. 2024 May 7;9(11):e164771. doi: 10.1172/jci.insight.164771 (PMC11382888; doi:10.1172/jci.insight.164771)

Fig1H

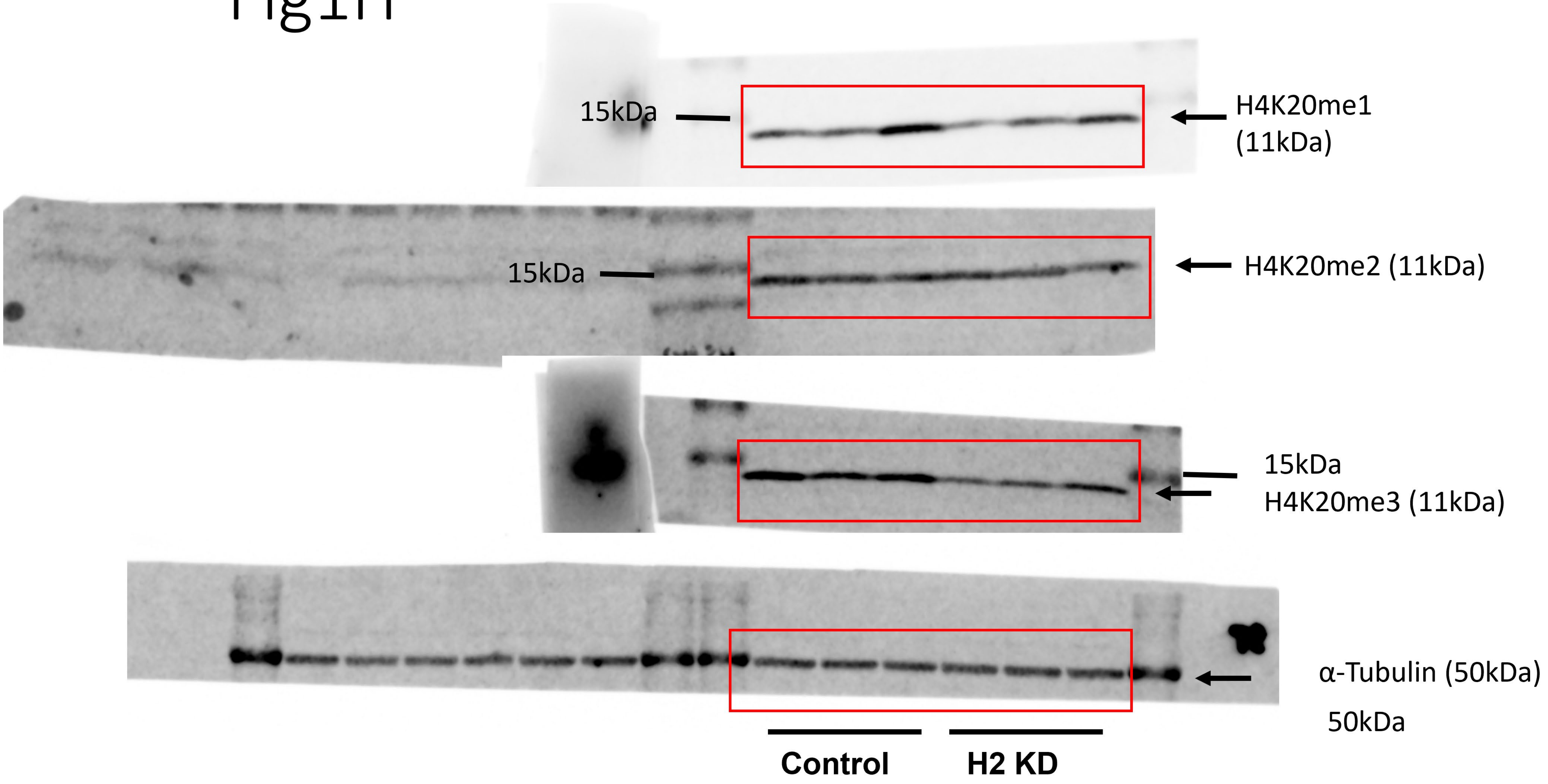
15kDa
H4K20me1
(11kDa)
15kDa
H4K20me2 (11kDa)
15kDa
H4K20me3 (11kDa)
α-Tubulin (50kDa)
50kDa
Control
H2 KD

Fig1I

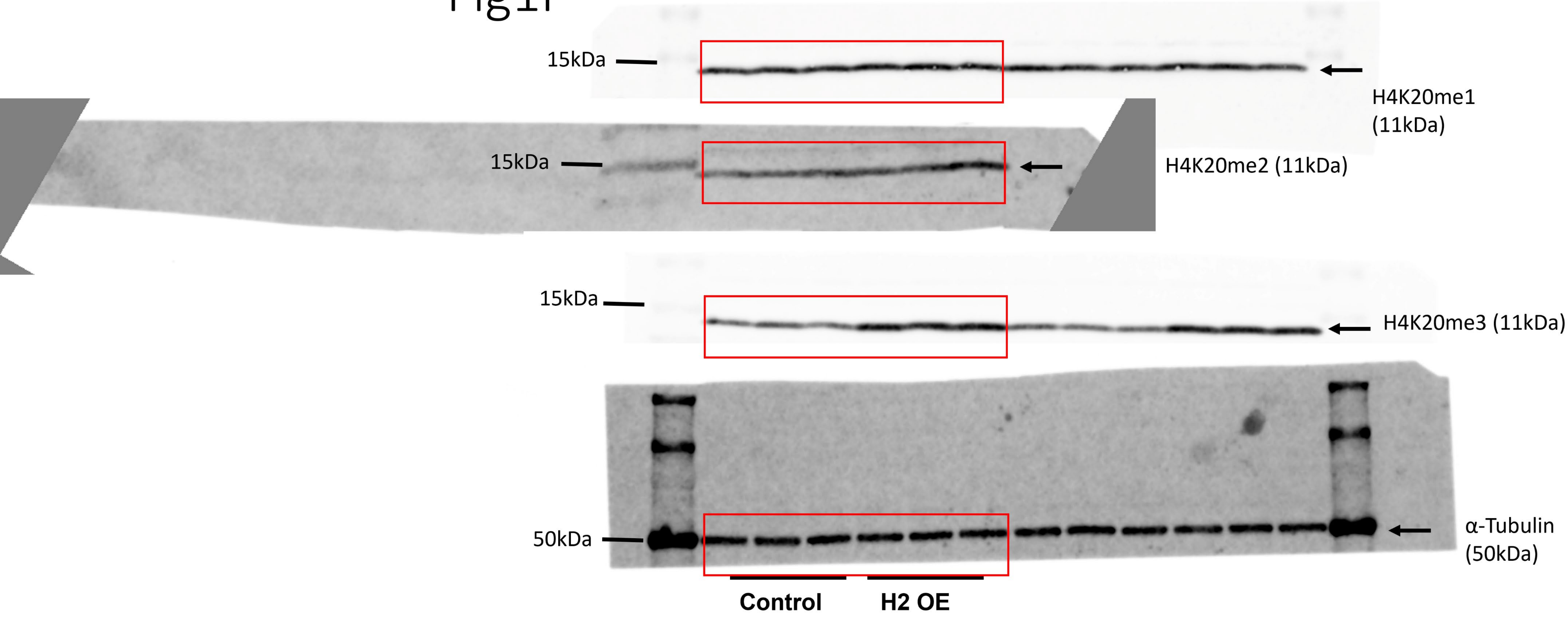
15kDa
H4K20me1 (11kDa)
15kDa
H4K20me2 (11kDa)
15kDa
H4K20me3 (11kDa)
50kDa
α-Tubulin (50kDa)
Control
H2 OE

Fig2A iBAT

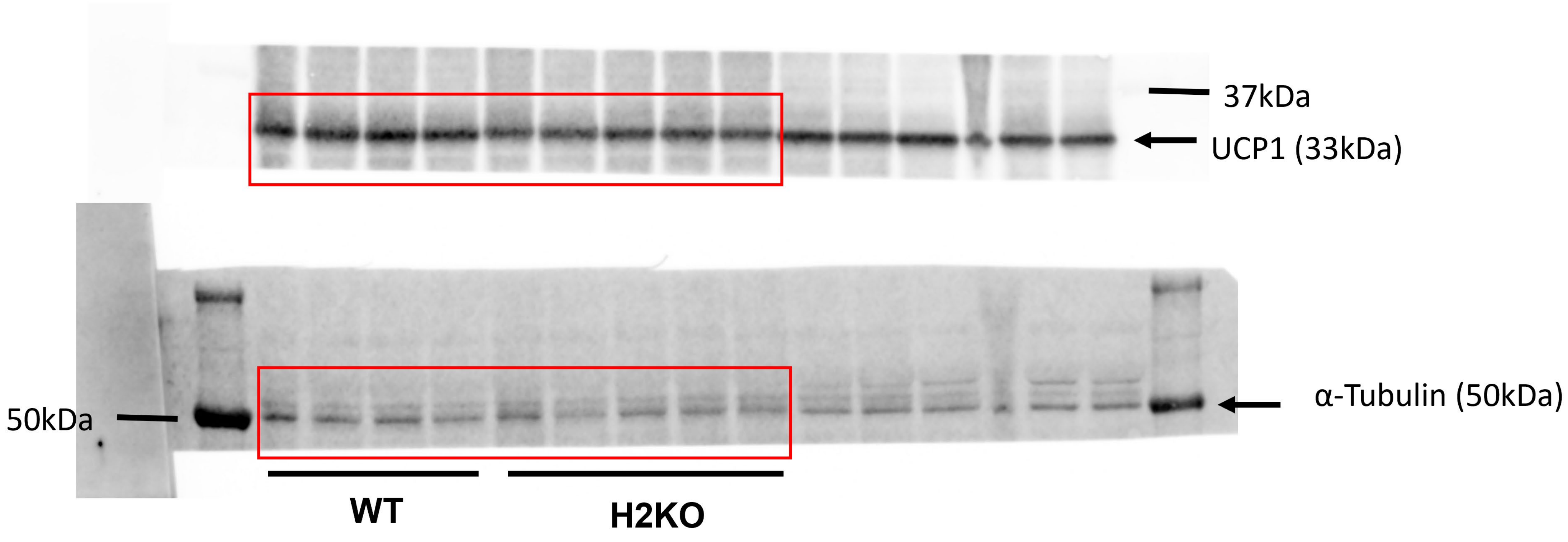

Fig2E iWAT

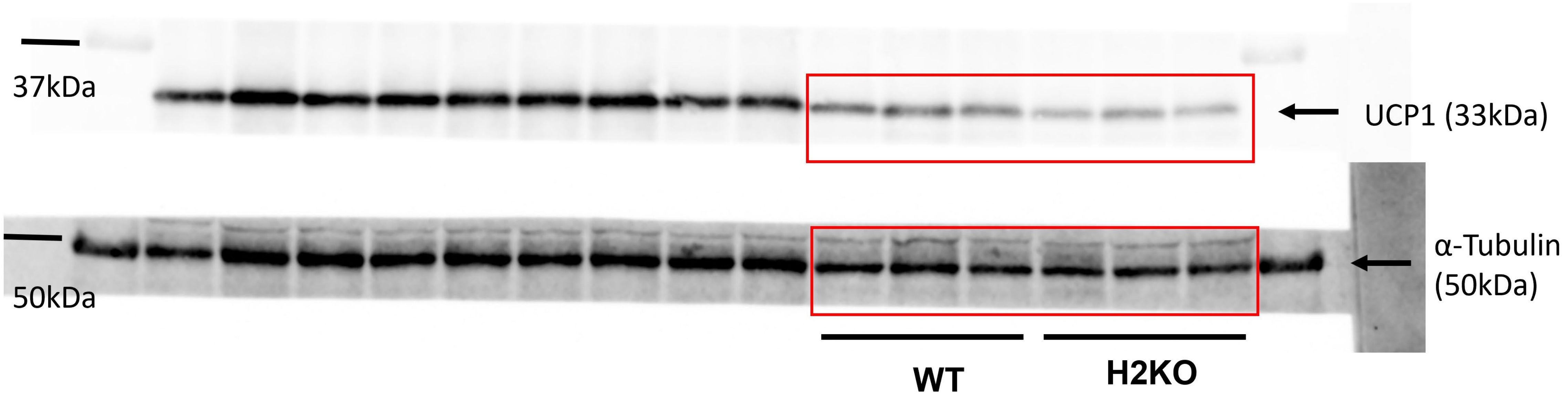

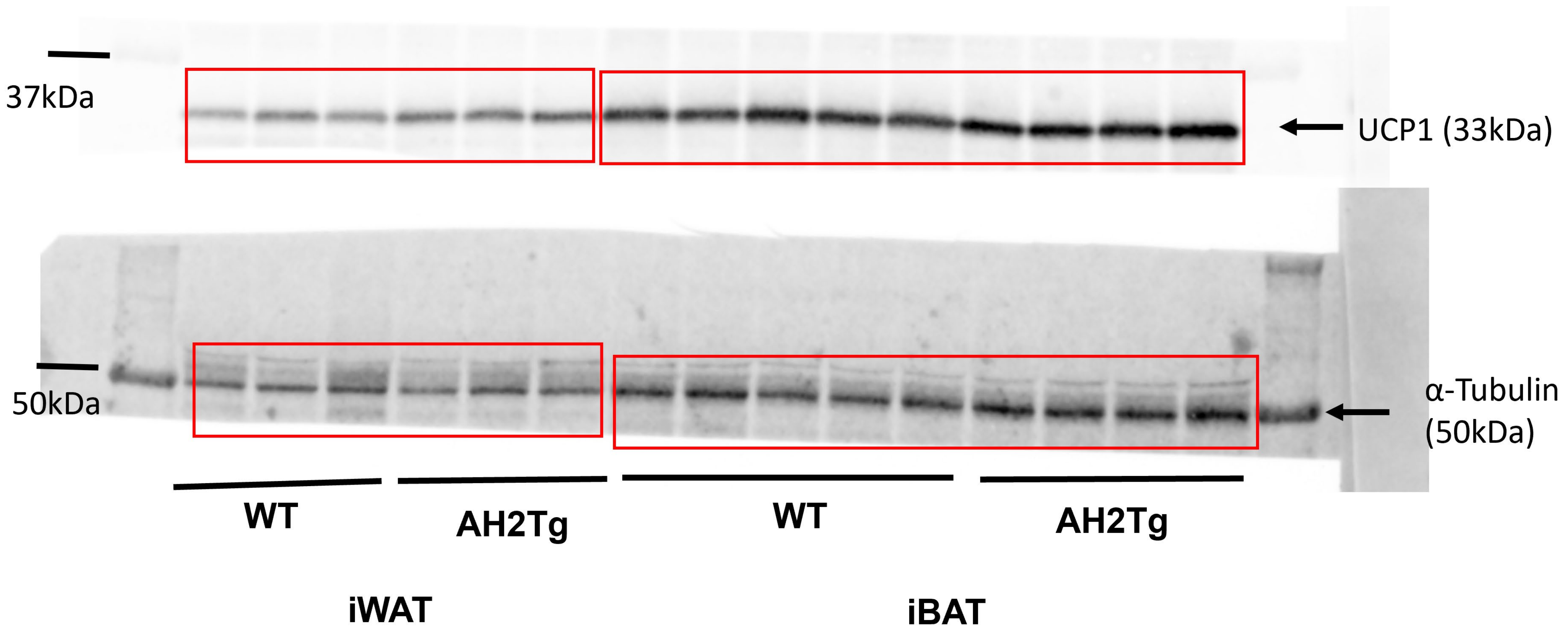
37kDa
UCP1 (33kDa)
50kDa
α-Tubulin
(50kDa)
WT
AH2Tg
WT
AH2Tg
iWAT
iBAT

Fig3G

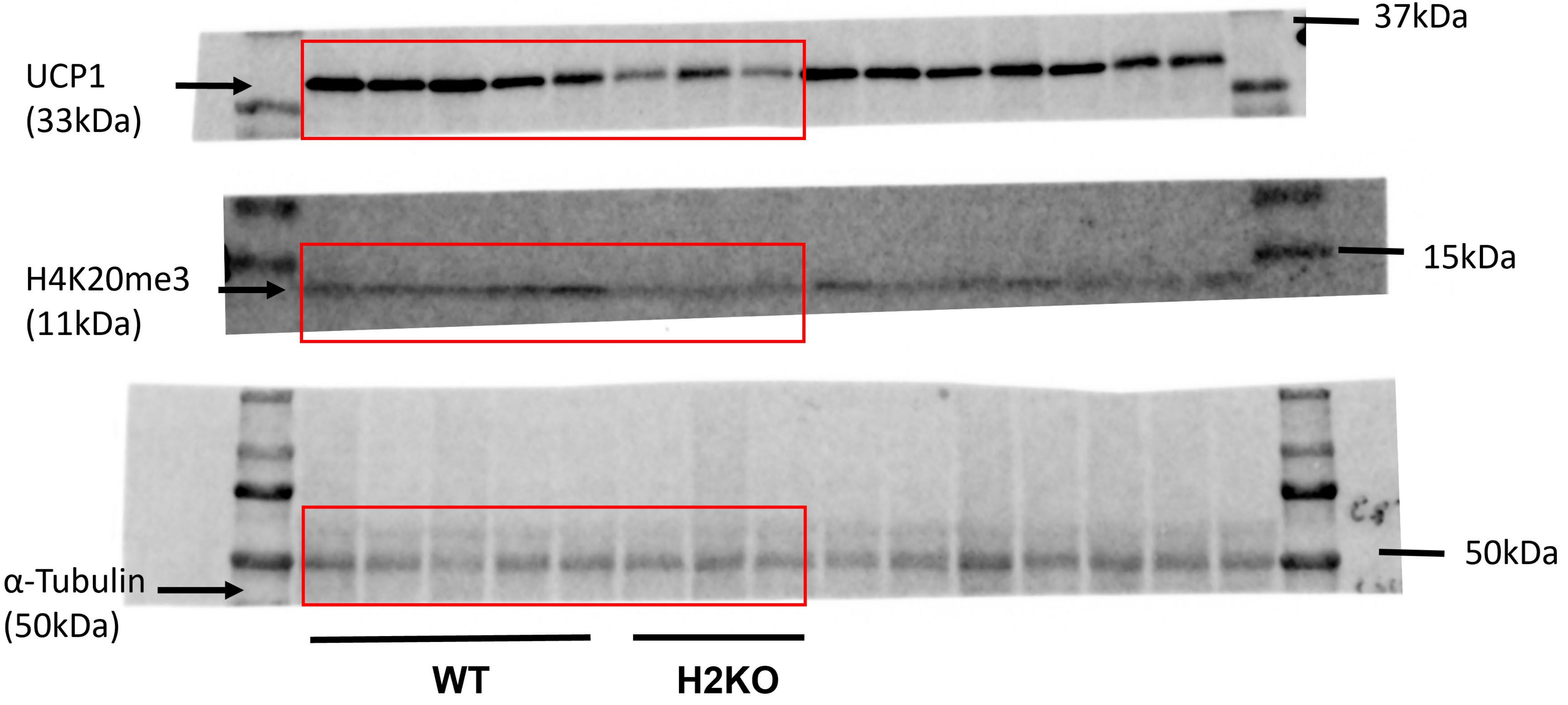

UCP1
(33kDa)
37kDa
H4K20me3
(11kDa)
15kDa
α-Tubulin
(50kDa)
50kDa
WT
H2KO

Fig3H

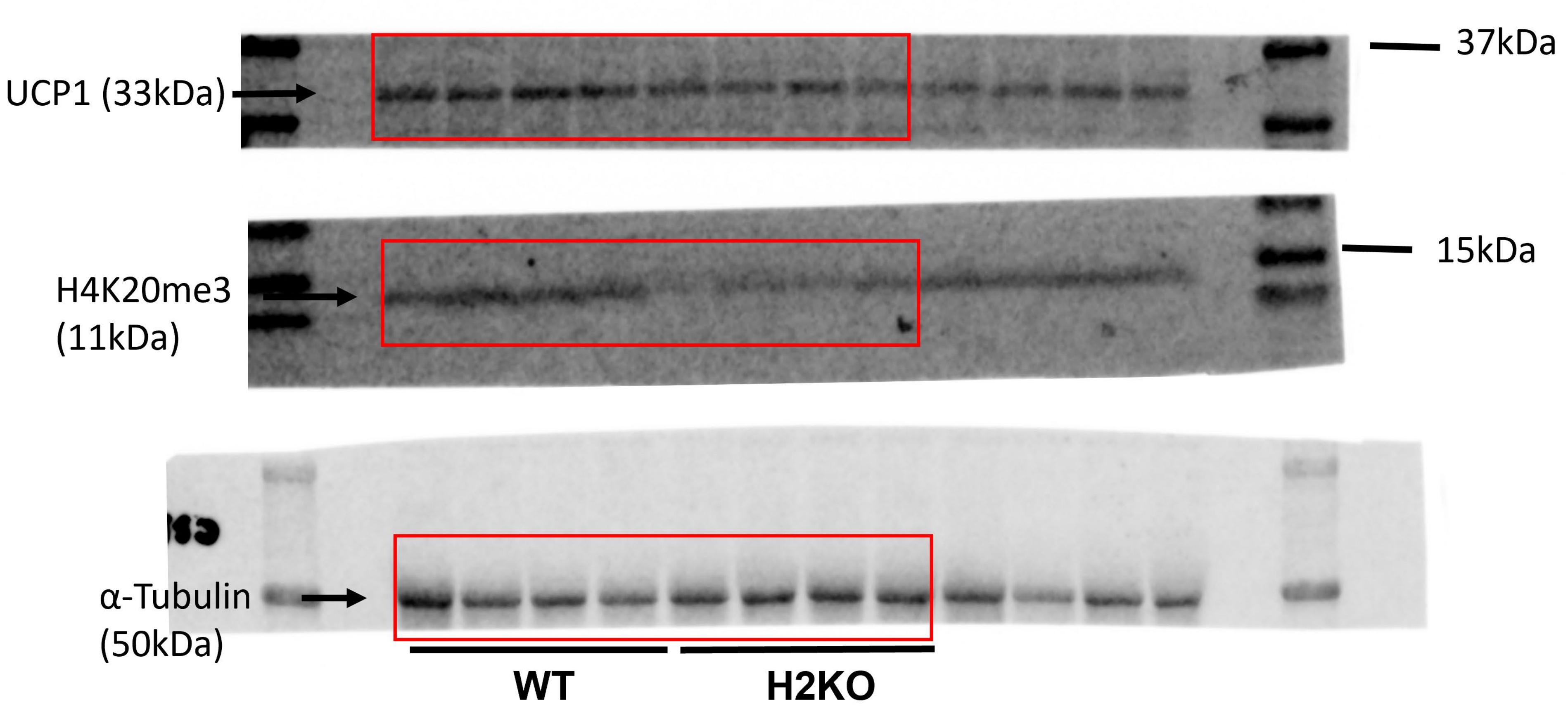

UCP1 (33kDa)
37kDa
H4K20me3
(11kDa)
15kDa
α-Tubulin
(50kDa)
WT
H2KO

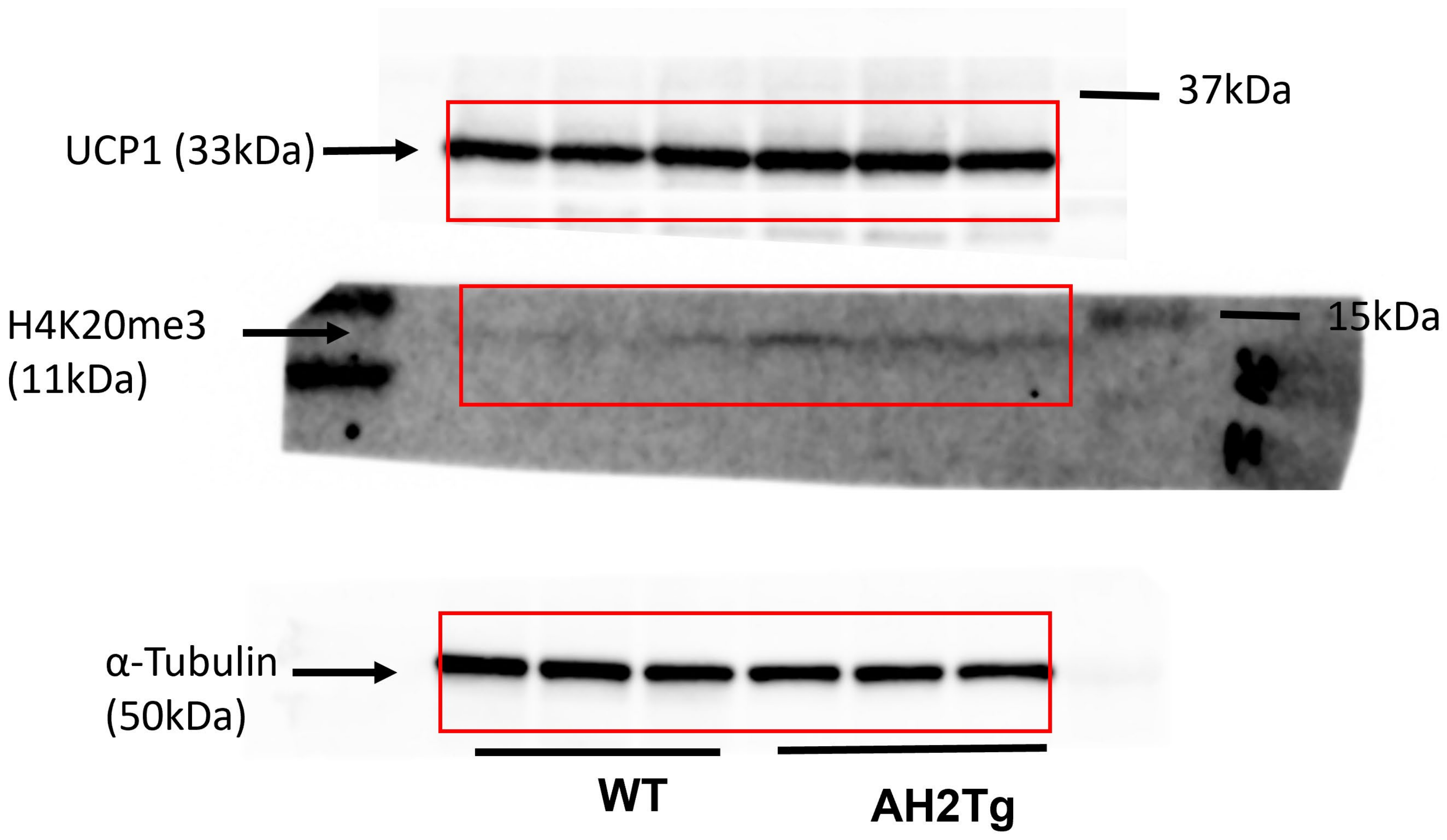

UCP1 (33kDa)
37kDa
H4K20me3
(11kDa)
15kDa
α-Tubulin
(50kDa)
WT
AH2Tg

Fig4H

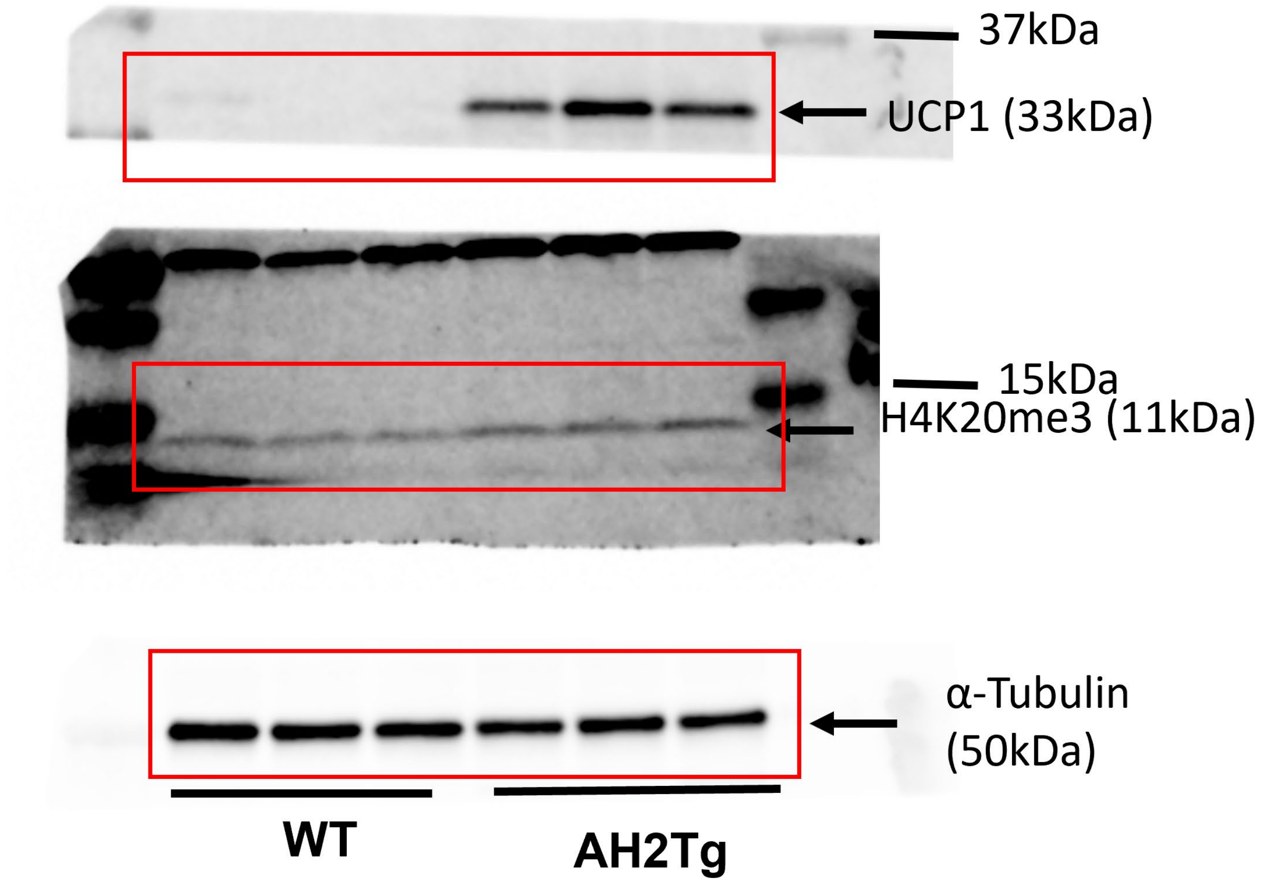

37kDa
UCP1 (33kDa)
15kDa
H4K20me3 (11kDa)
α-Tubulin
(50kDa)
WT
AH2Tg

Fig 6A

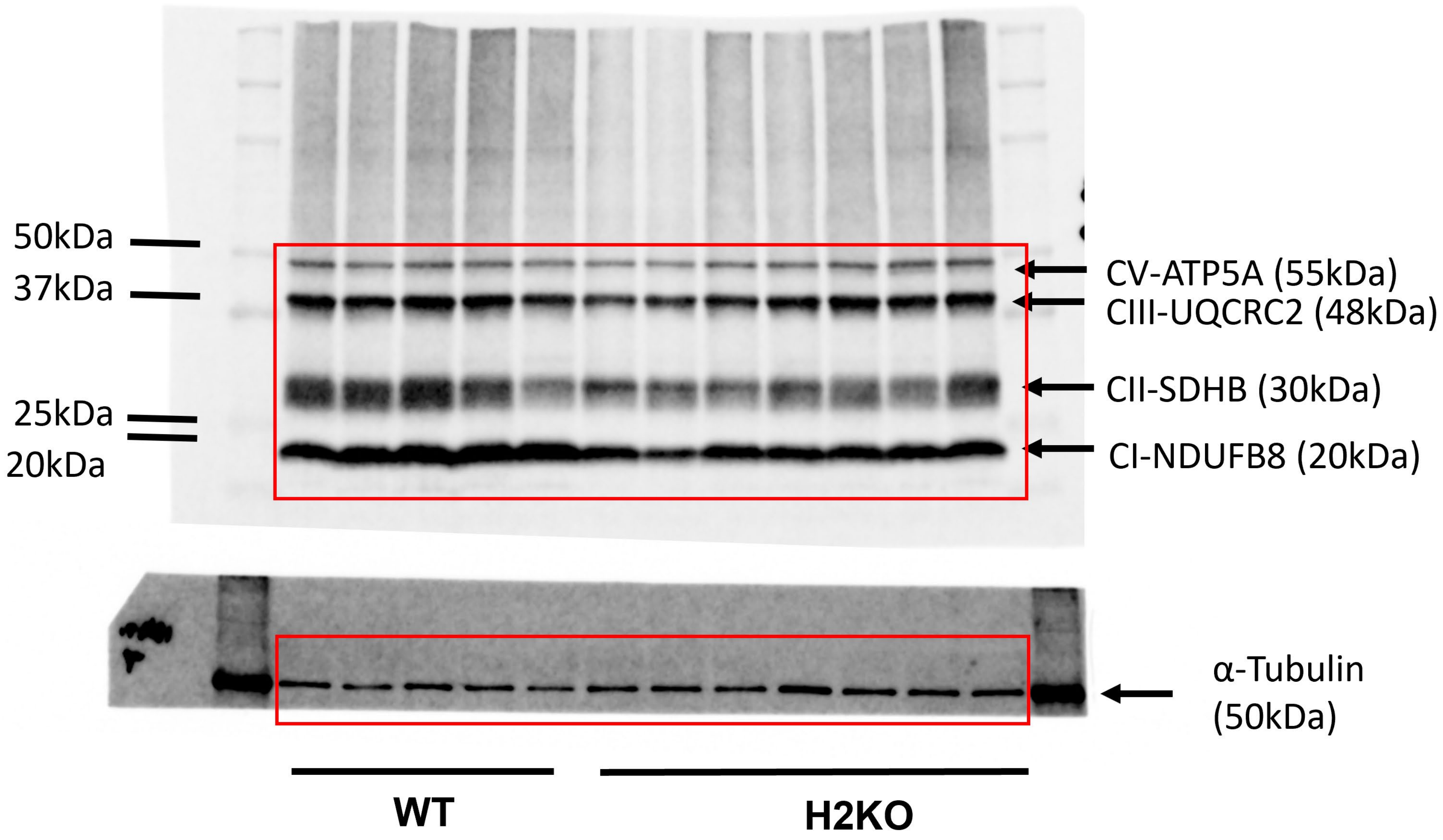

50kDa
37kDa
25kDa
20kDa
CV-ATP5A (55kDa)
CIII-UQCRC2 (48kDa)
CII-SDHB (30kDa)
CI-NDUFB8 (20kDa)
α-Tubulin
(50kDa)
WT
H2KO

Fig6B

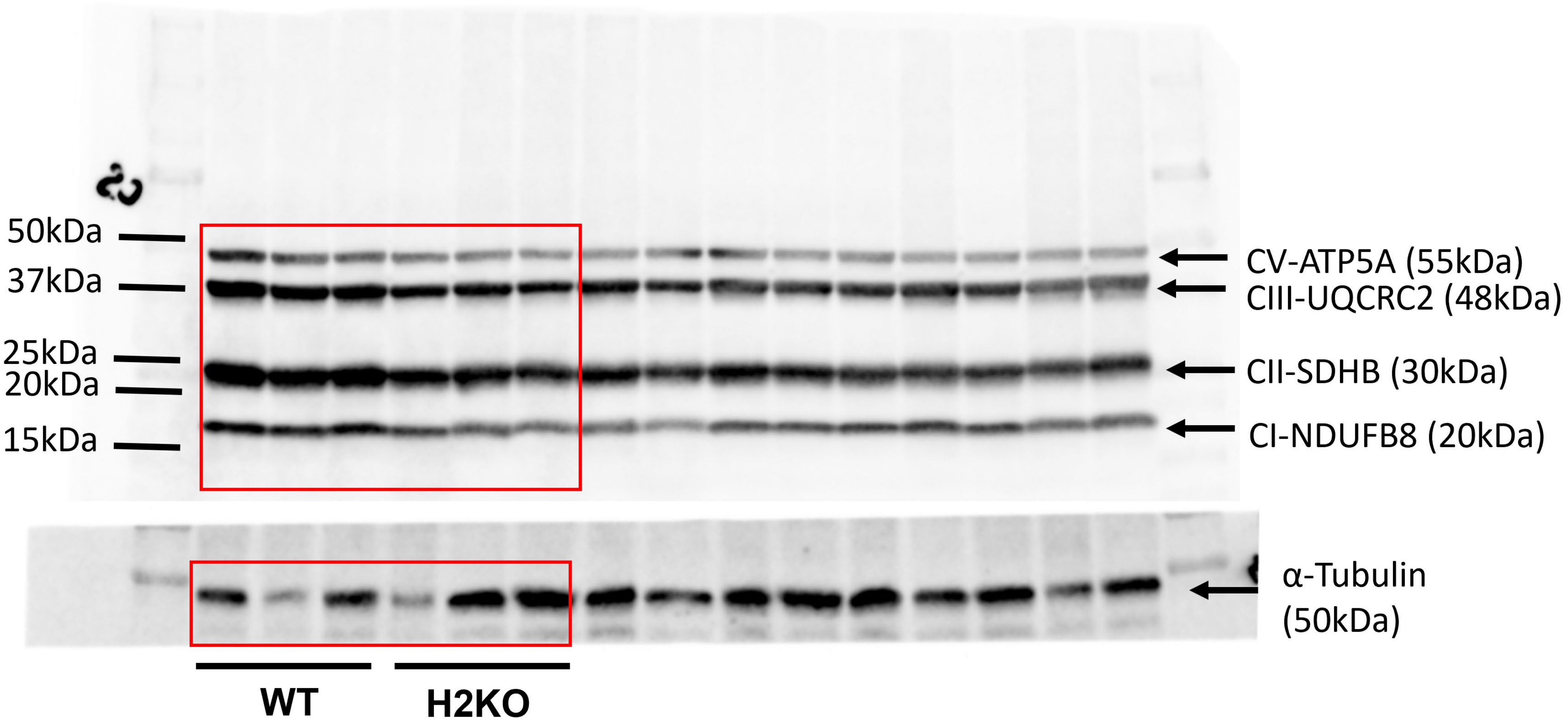
50kDa
37kDa
25kDa
20kDa
15kDa
CV-ATP5A (55kDa)
CIII-UQCRC2 (48kDa)
CII-SDHB (30kDa)
CI-NDUFB8 (20kDa)
α-Tubulin
(50kDa)
WT
H2KO

Fig 6C

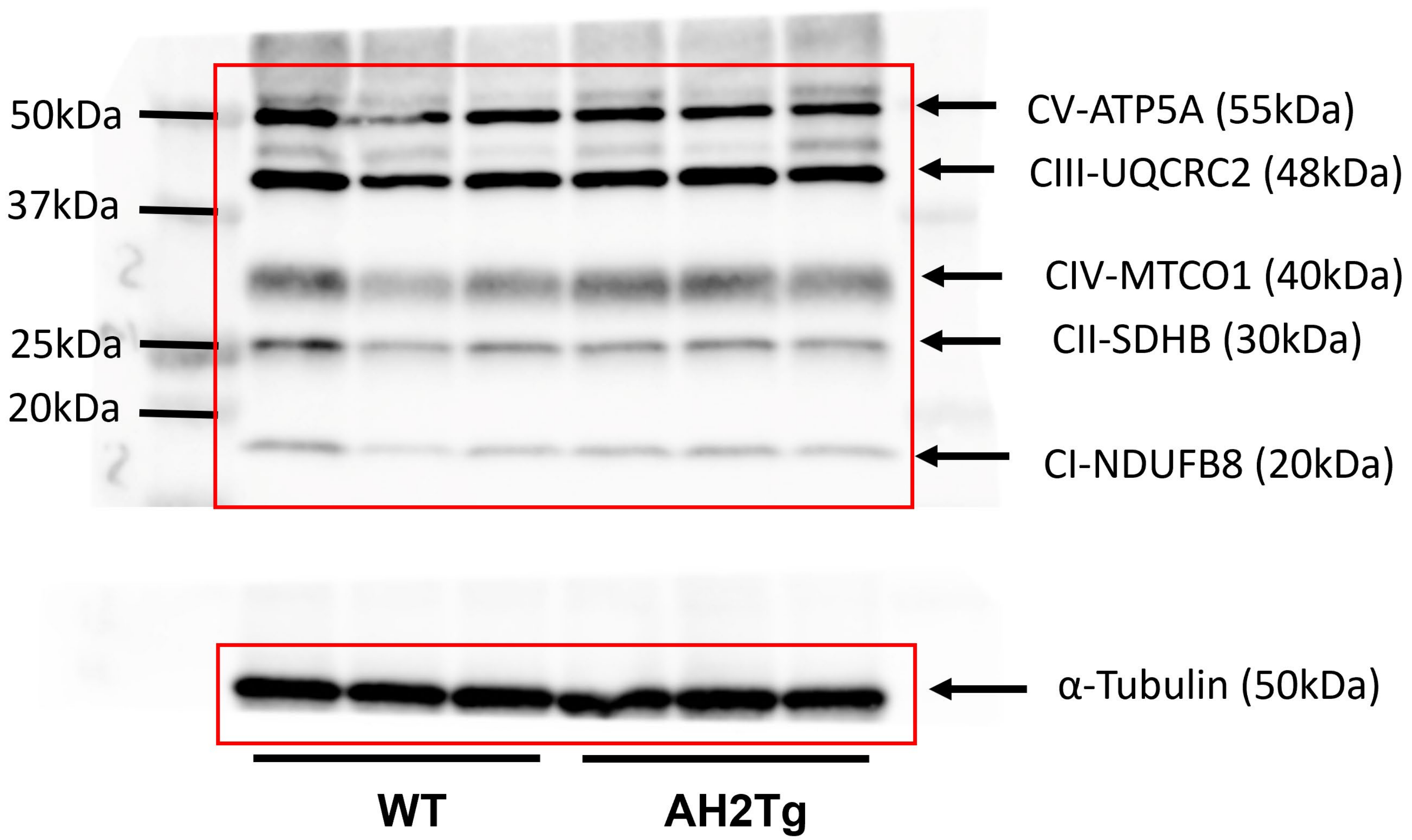

50kDa
37kDa
25kDa
20kDa
CV-ATP5A (55kDa)
CIII-UQCRC2 (48kDa)
CIV-MTCO1 (40kDa)
CII-SDHB (30kDa)
CI-NDUFB8 (20kDa)
α-Tubulin (50kDa)
WT
AH2Tg

Fig6D

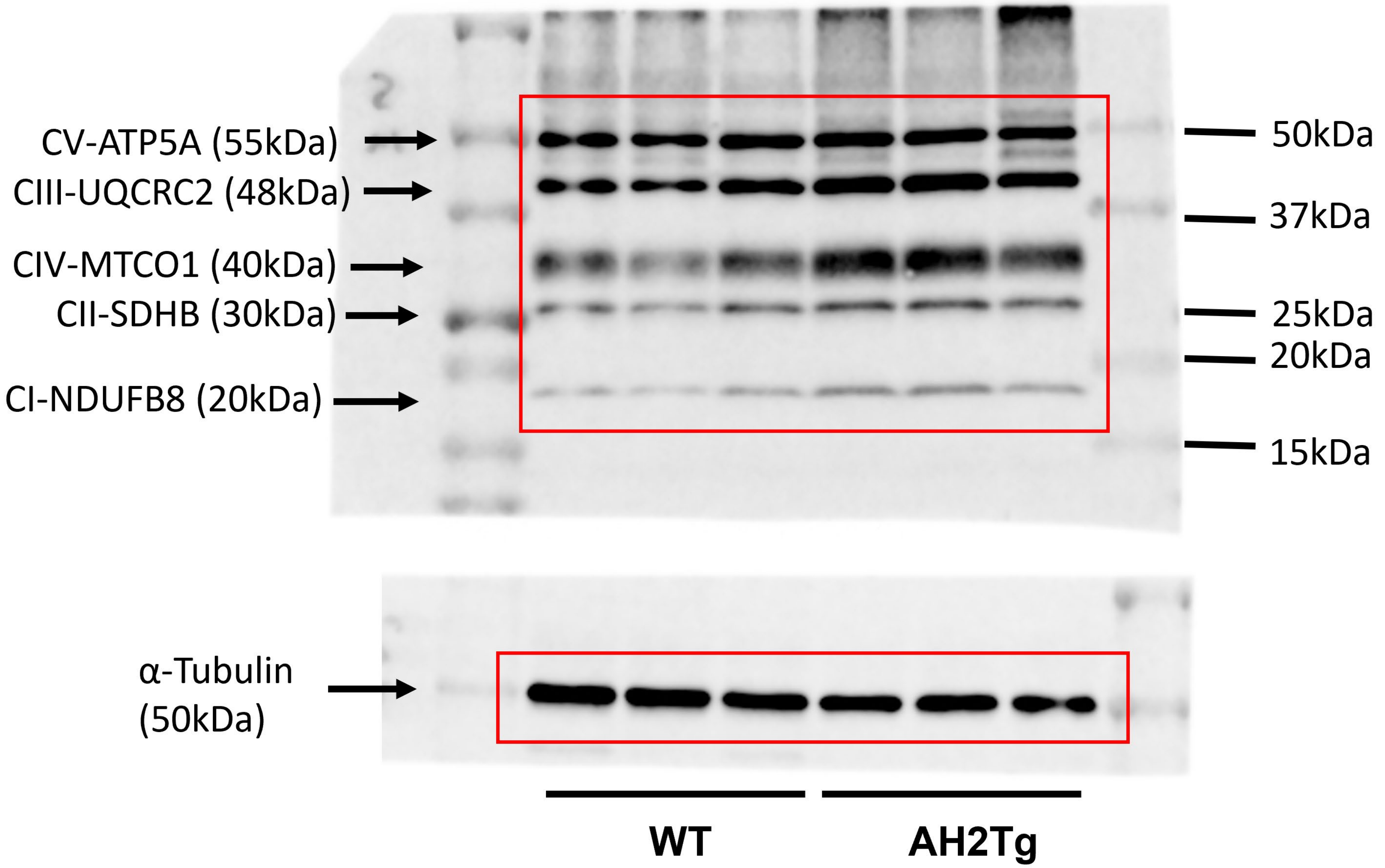

CV-ATP5A (55kDa)
CIII-UQCRC2 (48kDa)
CIV-MTCO1 (40kDa)
CII-SDHB (30kDa)
CI-NDUFB8 (20kDa)
50kDa
37kDa
25kDa
20kDa
15kDa
α-Tubulin (50kDa)
WT
AH2Tg

Fig 7B and 7D

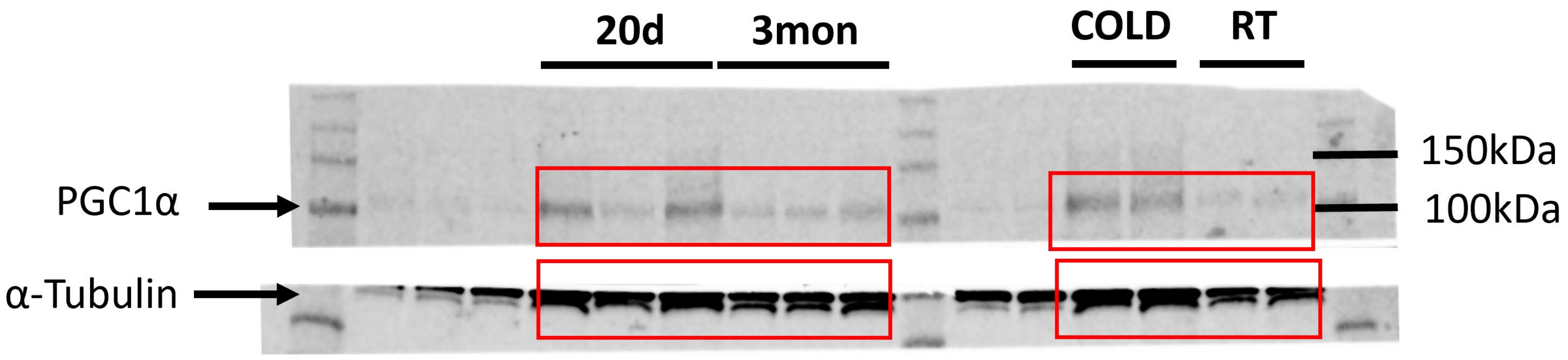
20d
3mon
COLD
RT
PGC1α
α-Tubulin
150kDa
100kDa

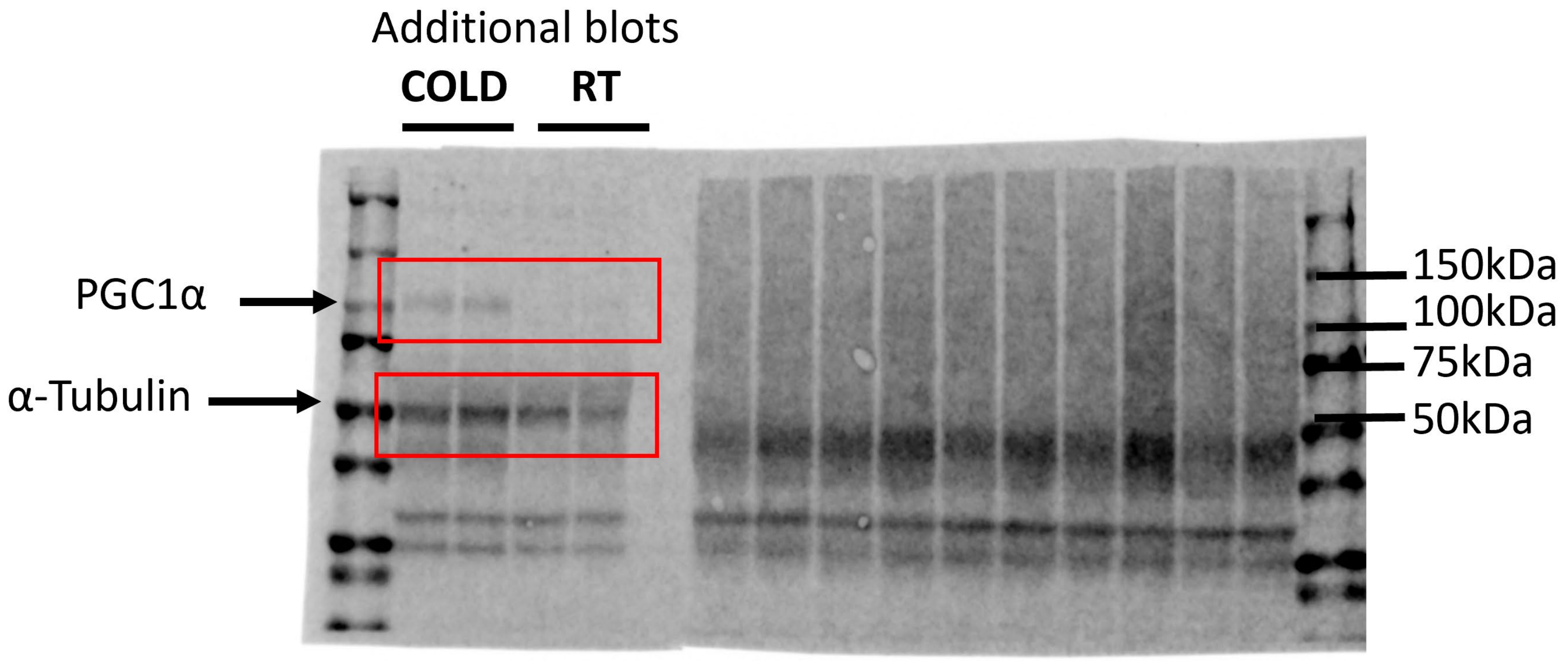
Additional blots
COLD
RT
PGC1α
α-Tubulin
150kDa
100kDa
75kDa
50kDa

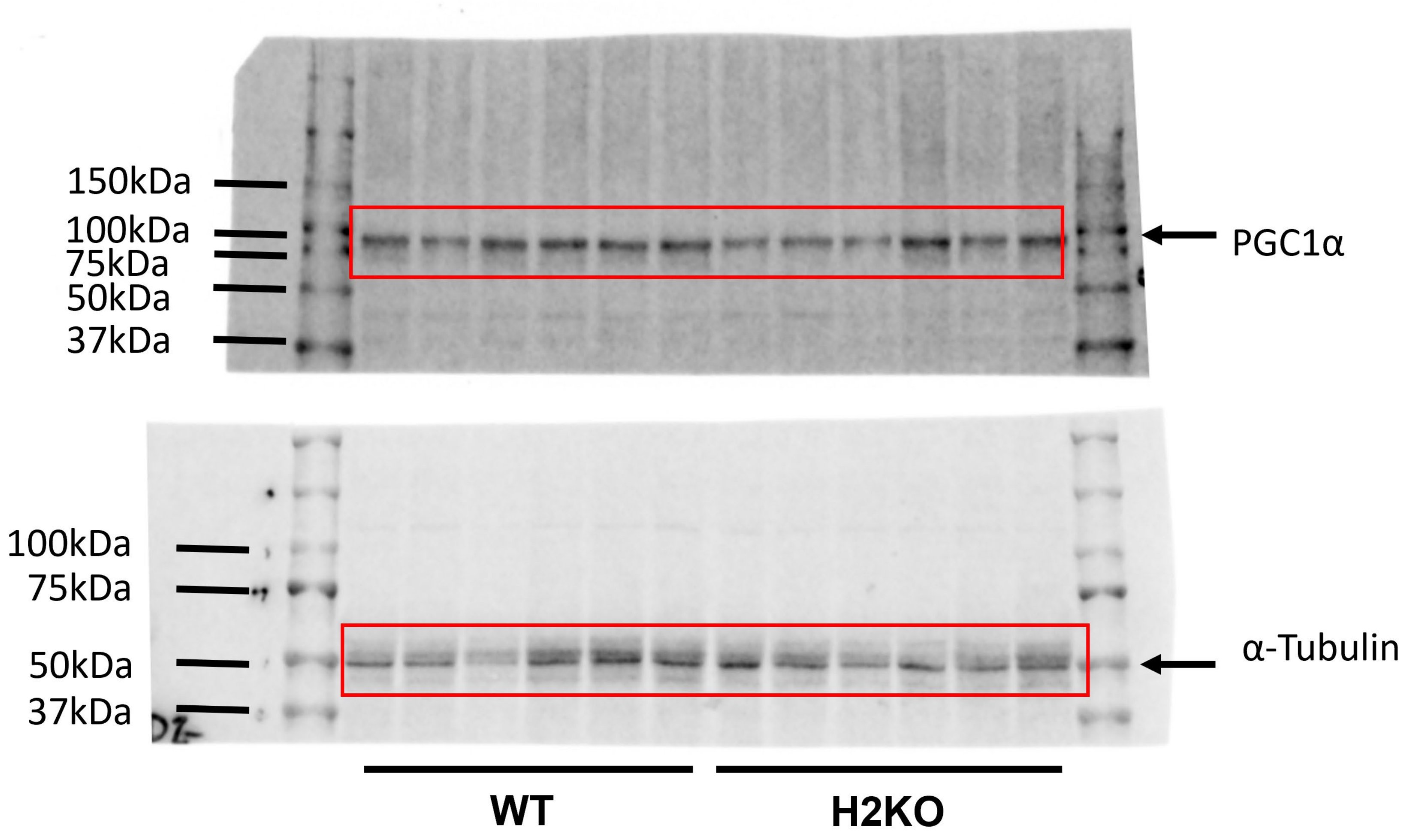
150kDa
100kDa
75kDa
50kDa
37kDa
PGC1α
100kDa
75kDa
50kDa
37kDa
α-Tubulin
WT
H2KO

Fig 7F

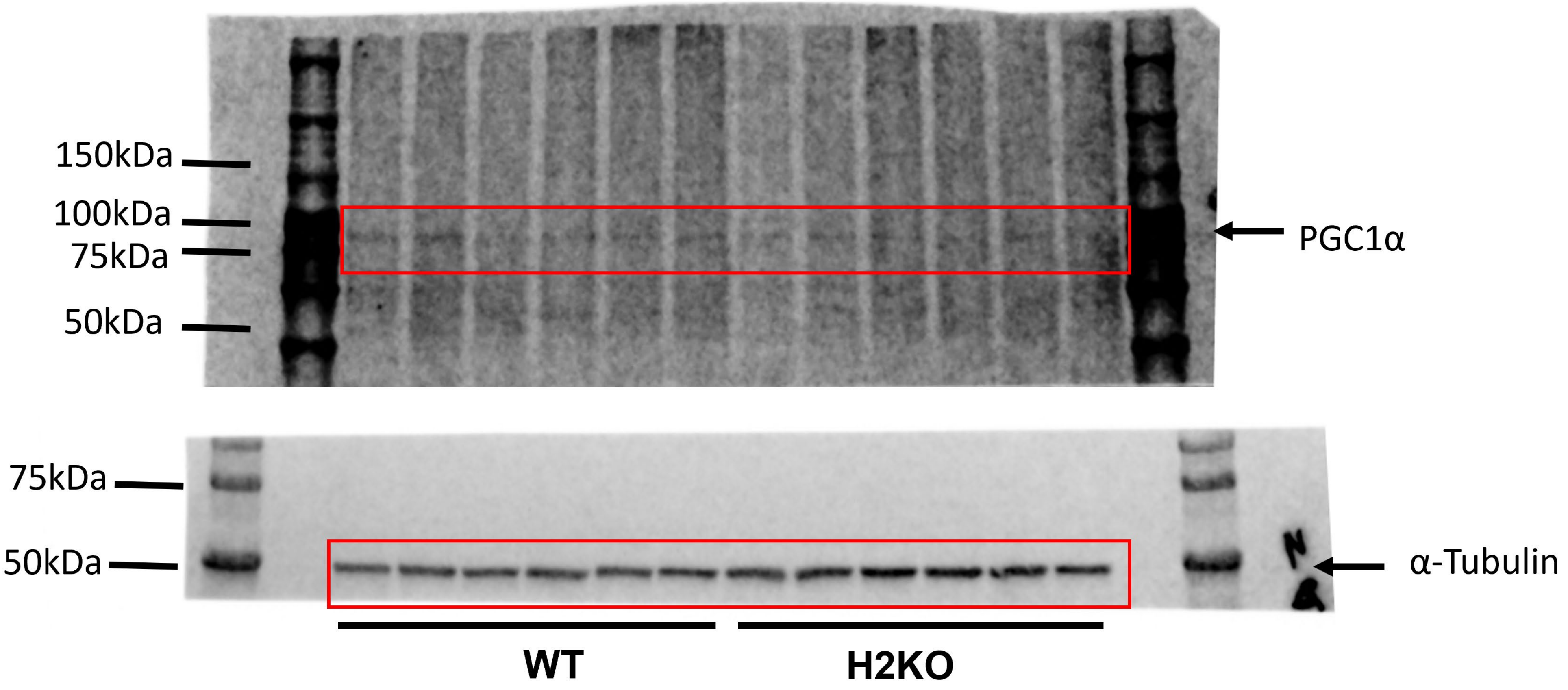
150kDa
100kDa
75kDa
50kDa
PGC1α
75kDa
50kDa
α-Tubulin
WT
H2KO

Fig 7G

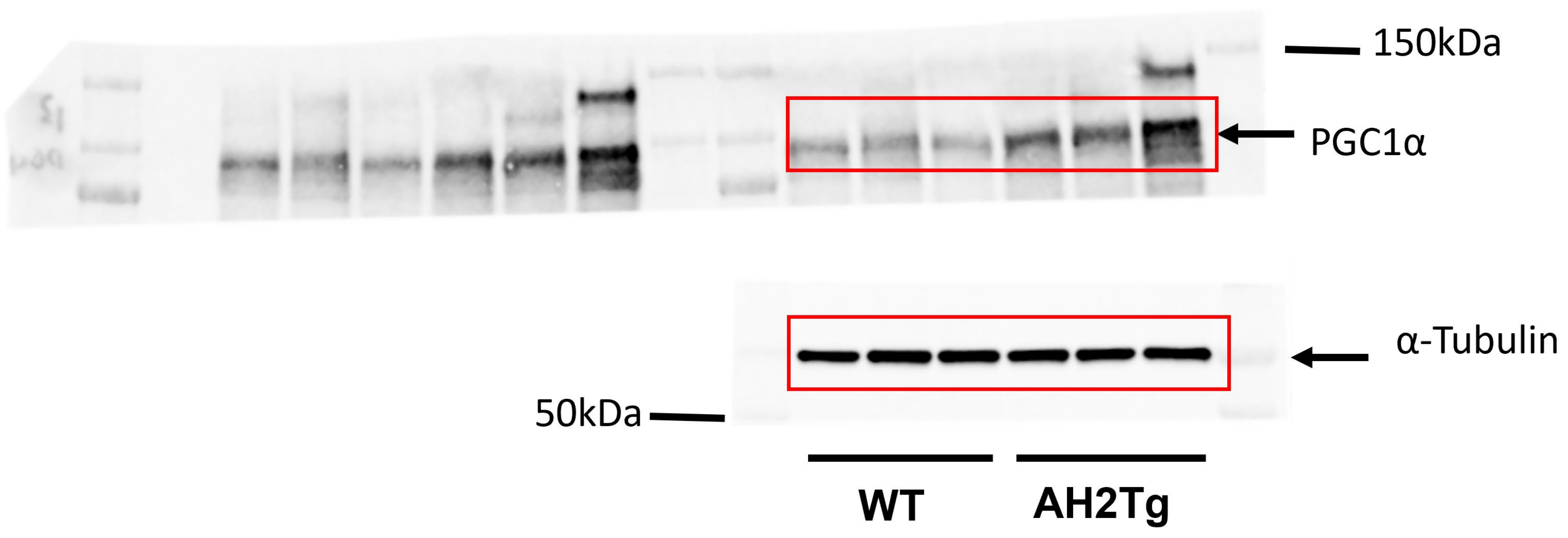

Fig 7H

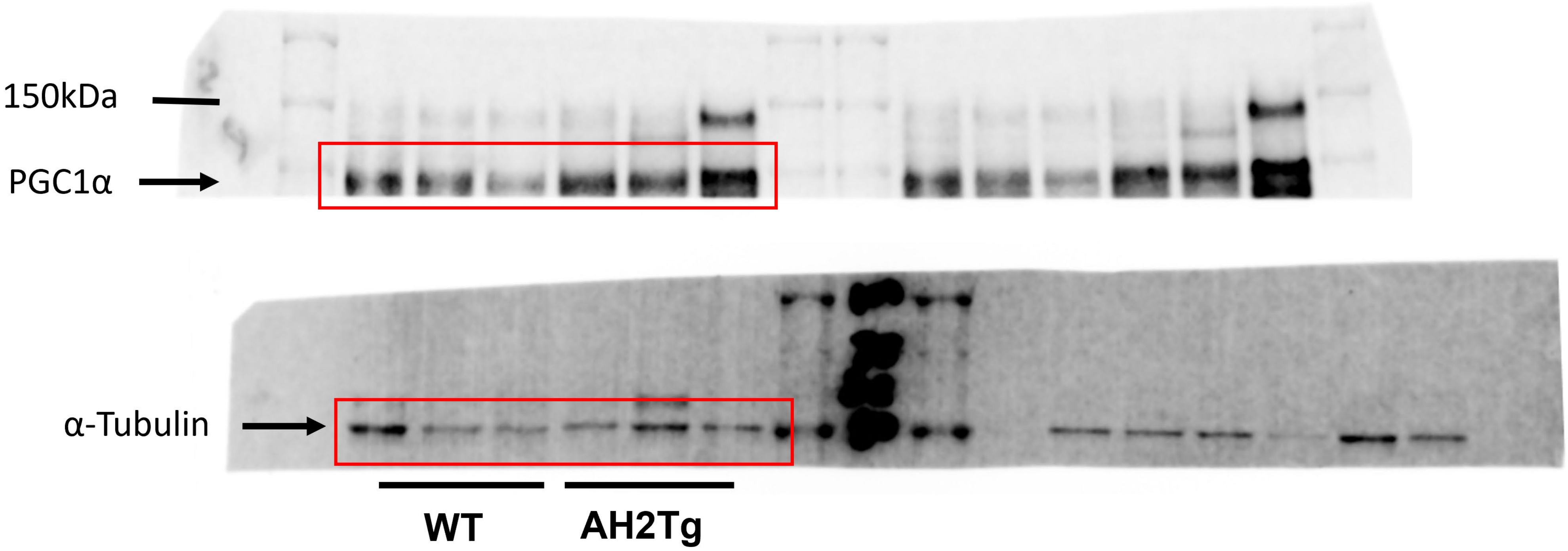

Fig 8D

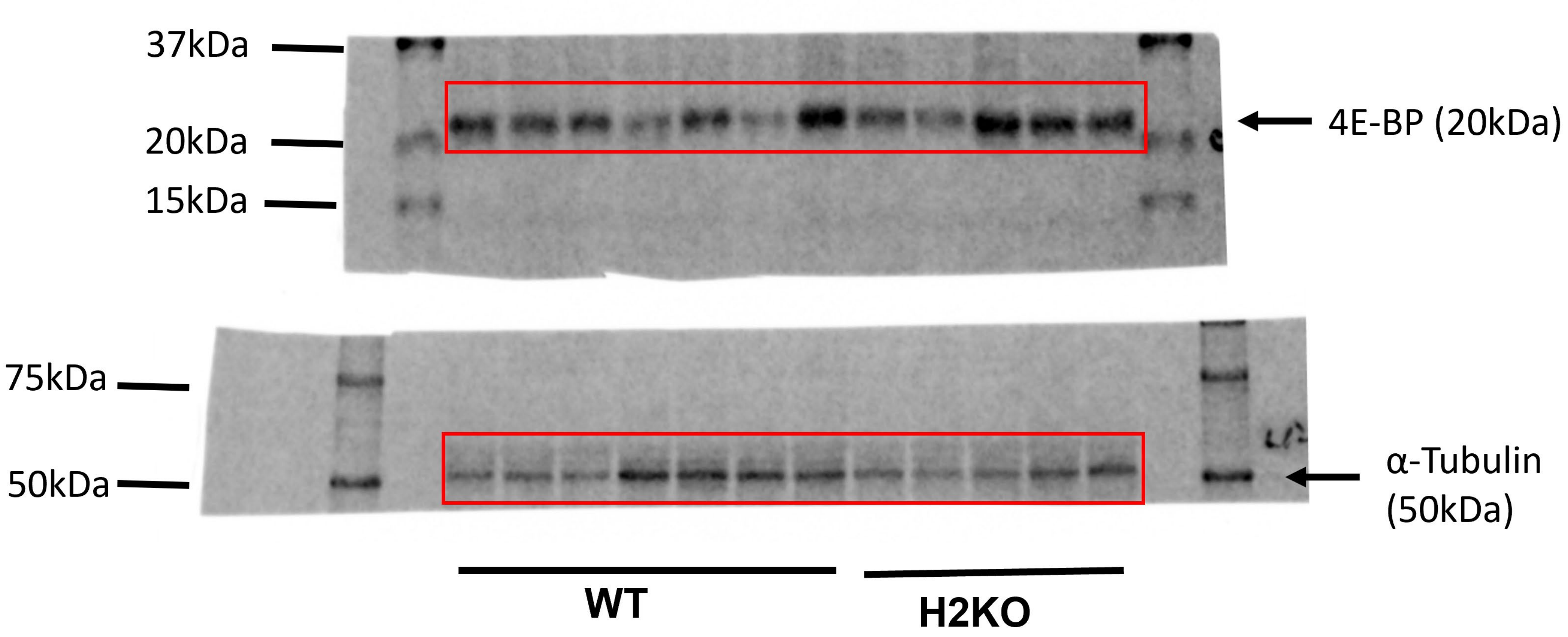
37kDa
20kDa
15kDa
4E-BP (20kDa)
75kDa
50kDa
α-Tubulin (50kDa)
WT
H2KO

Fig 8E

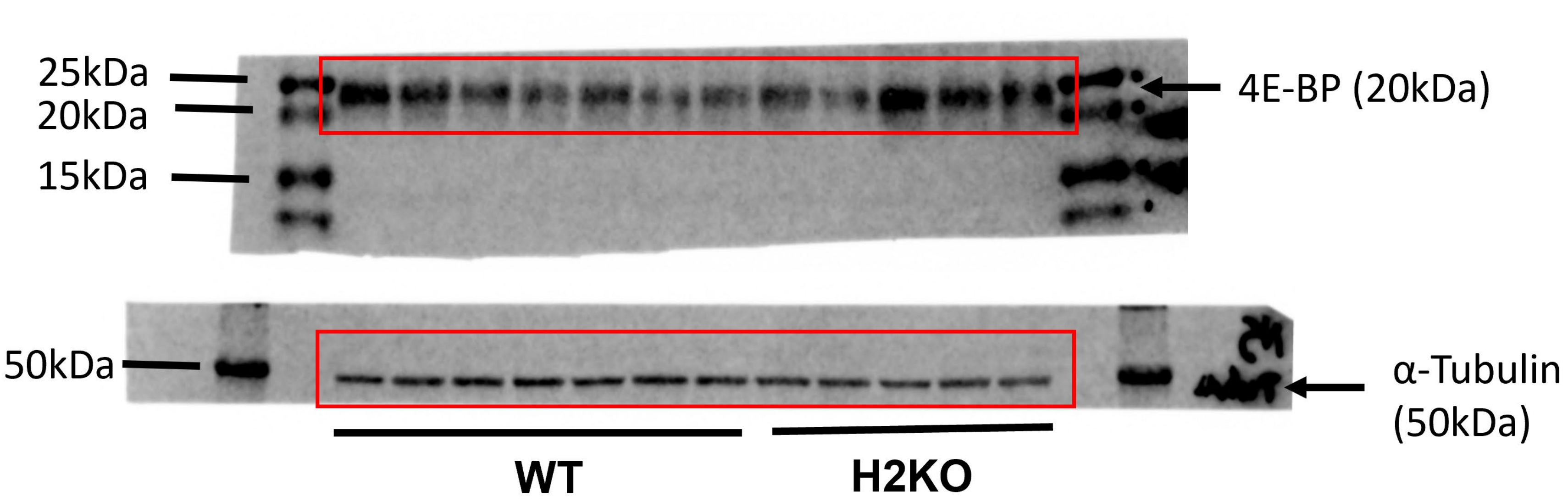

Fig 8F

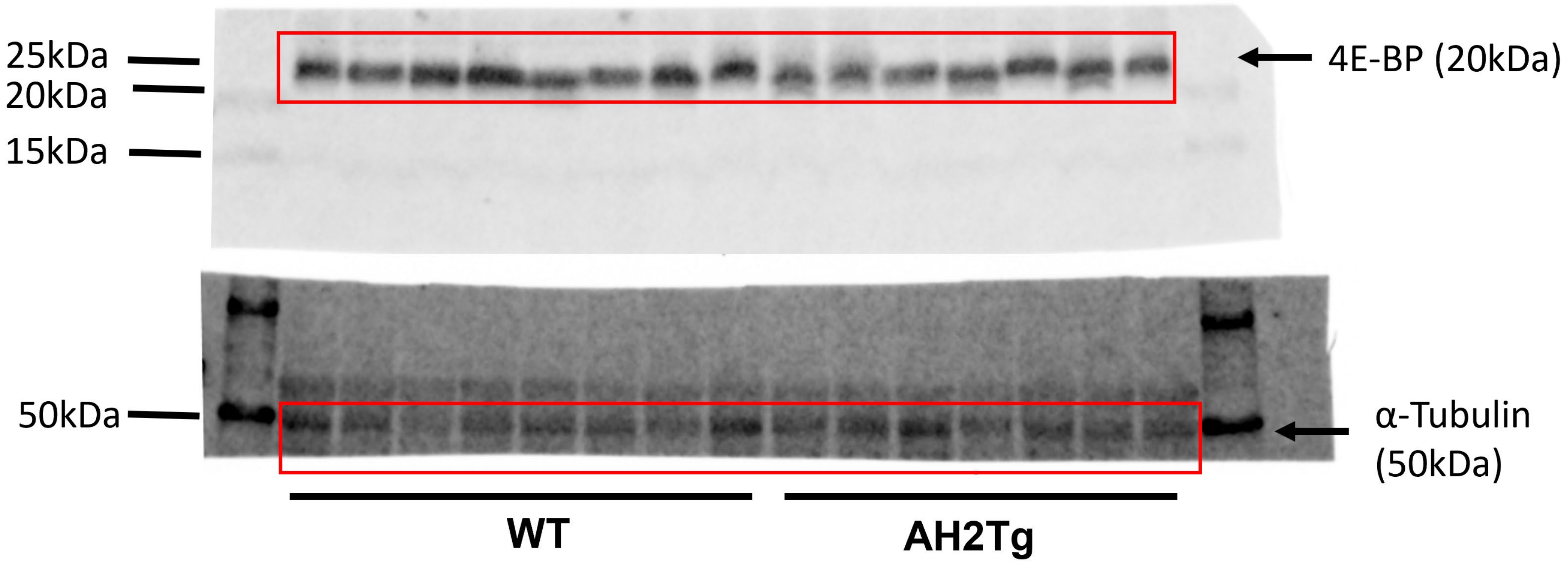

Fig 8G

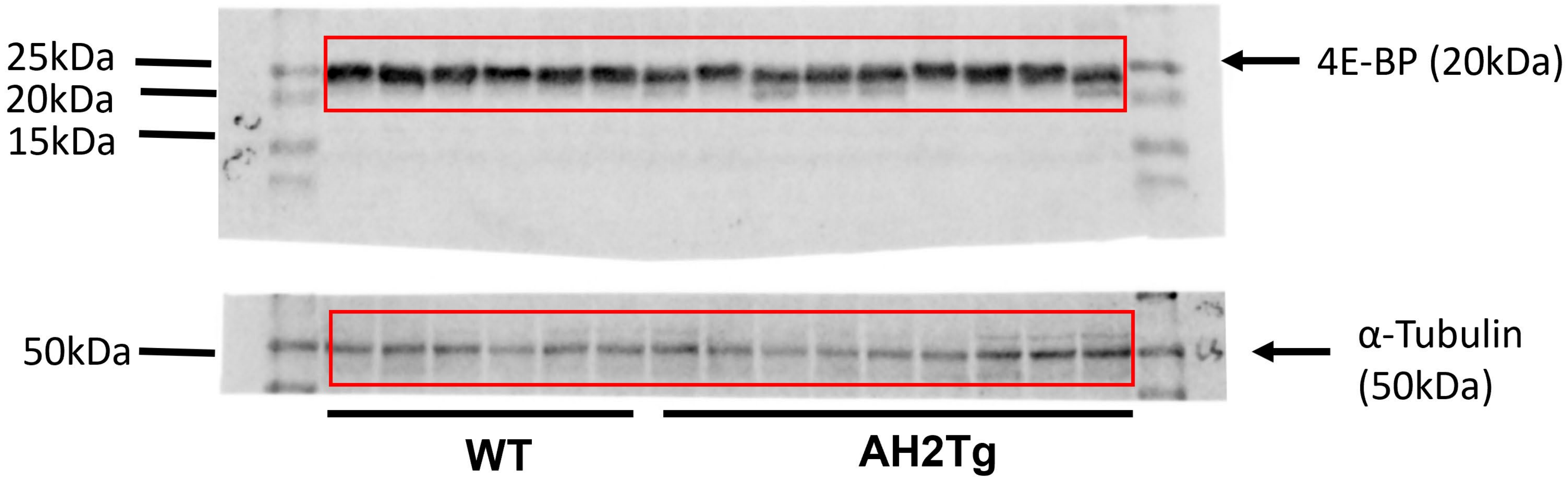

Fig 8H

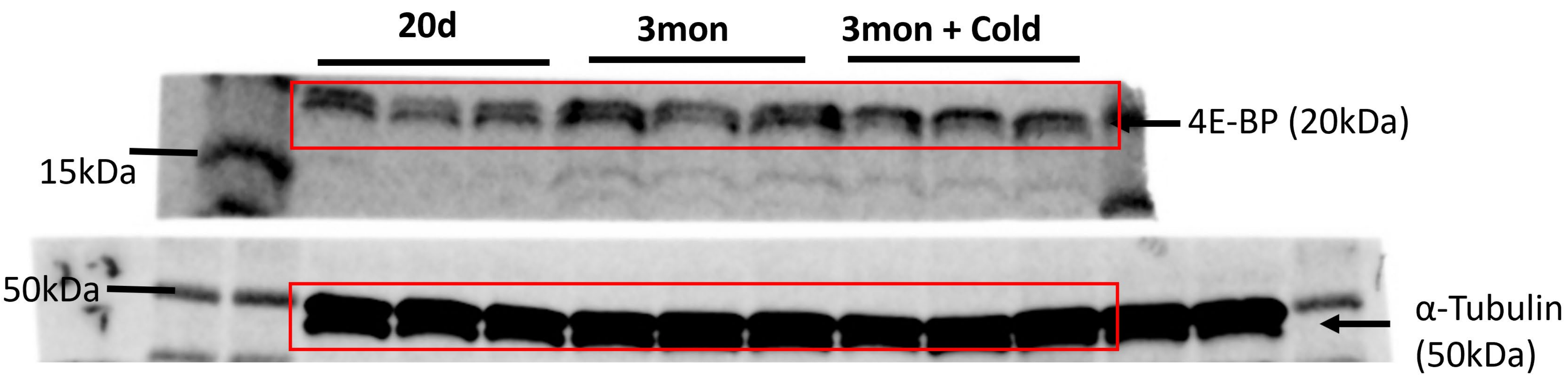

Fig 9E

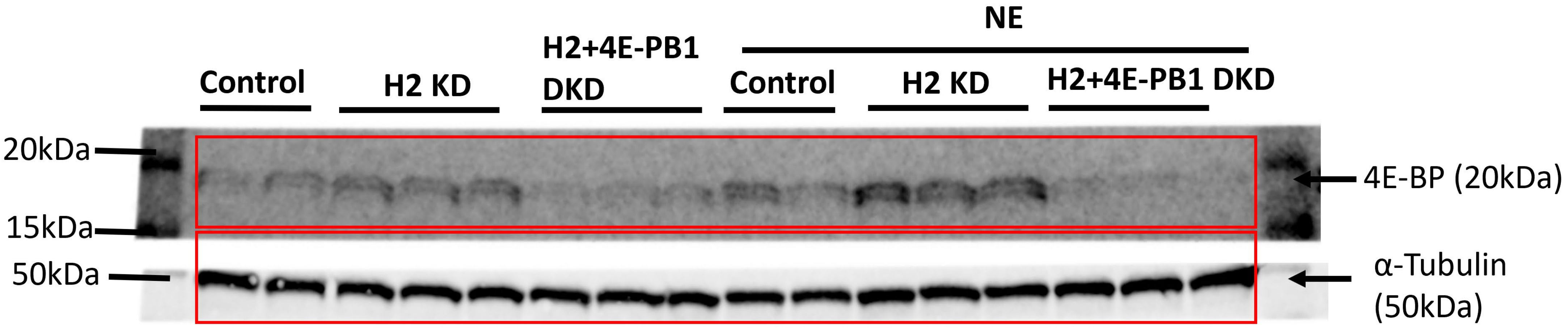
NE
Control
H2 KD
H2+4E-PB1 DKD
Control
H2 KD
H2+4E-PB1 DKD
20kDa
15kDa
50kDa
4E-BP (20kDa)
α-Tubulin (50kDa)

Fig 9F

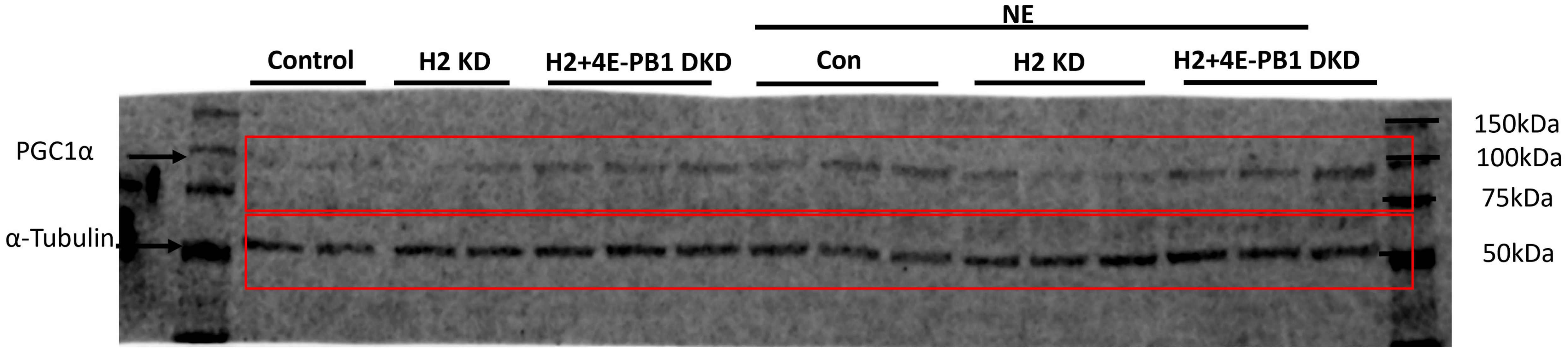
NE
Control
H2 KD
H2+4E-PB1 DKD
Con
H2 KD
H2+4E-PB1 DKD
PGC1α
α-Tubulin
150kDa
100kDa
75kDa
50kDa

Suppl. Fig 16B-C

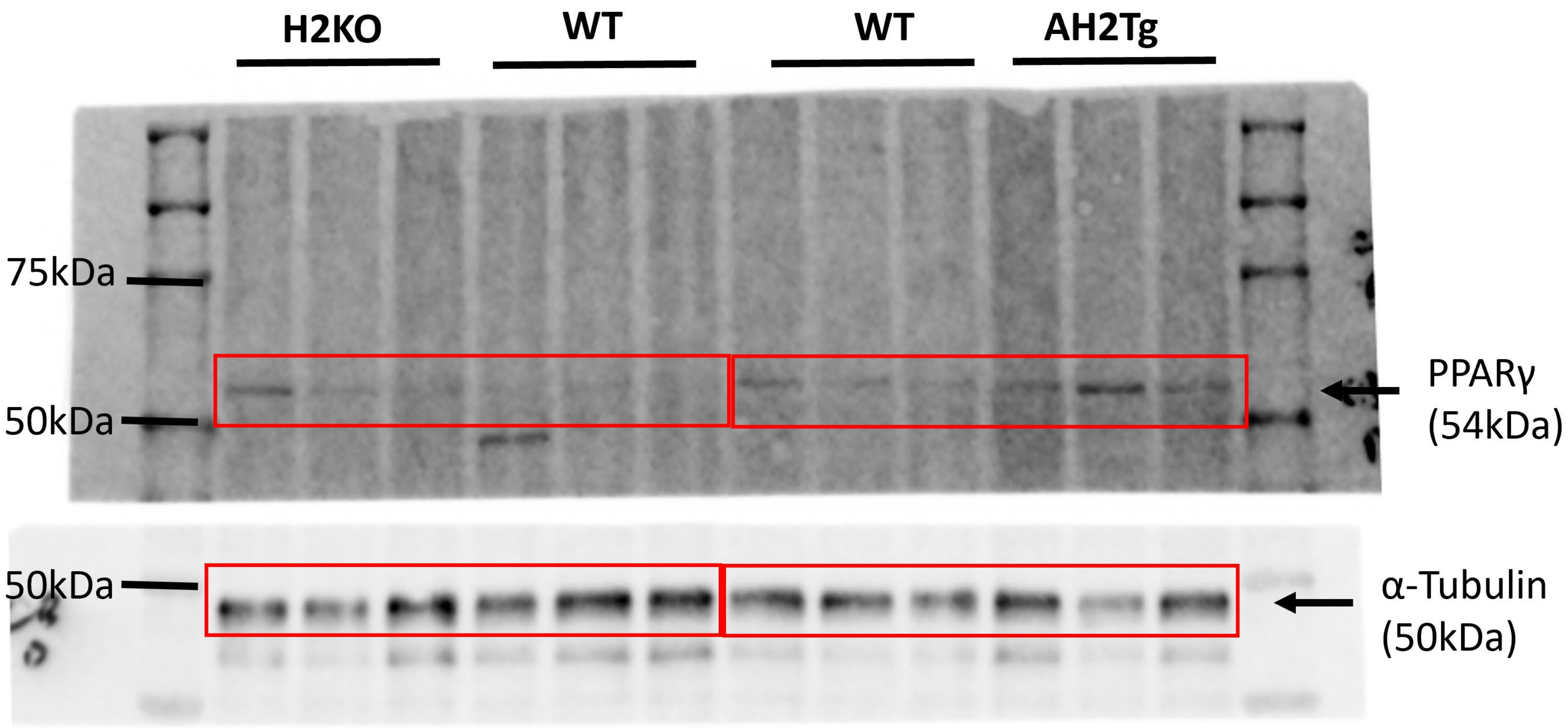

Suppl. Fig 17B-C

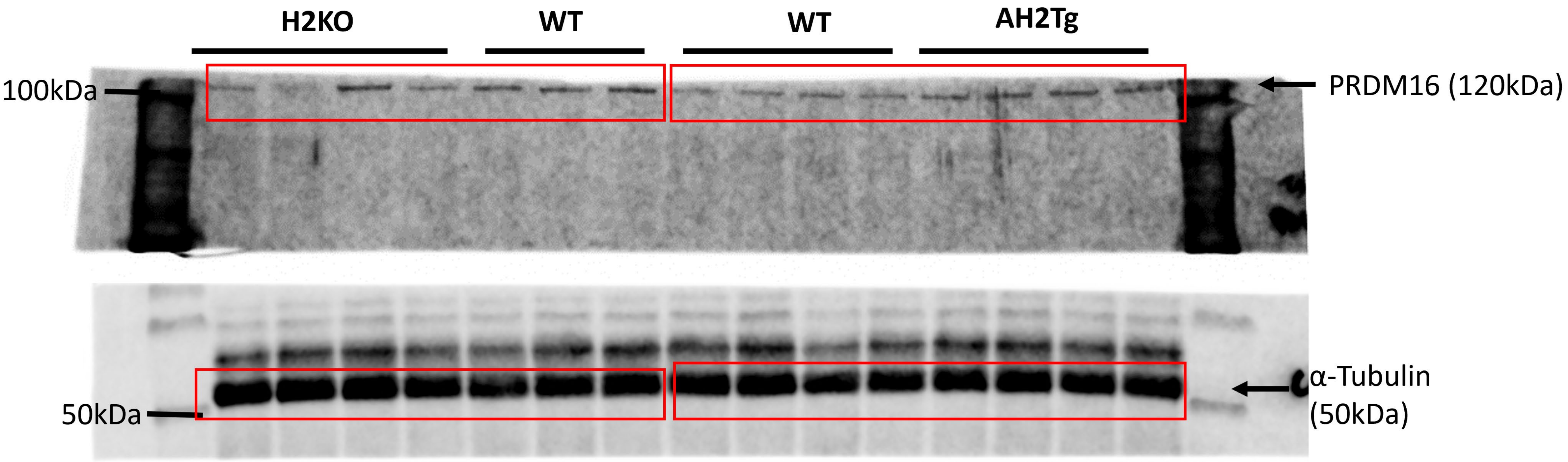

Suppl. Fig 20 C-E

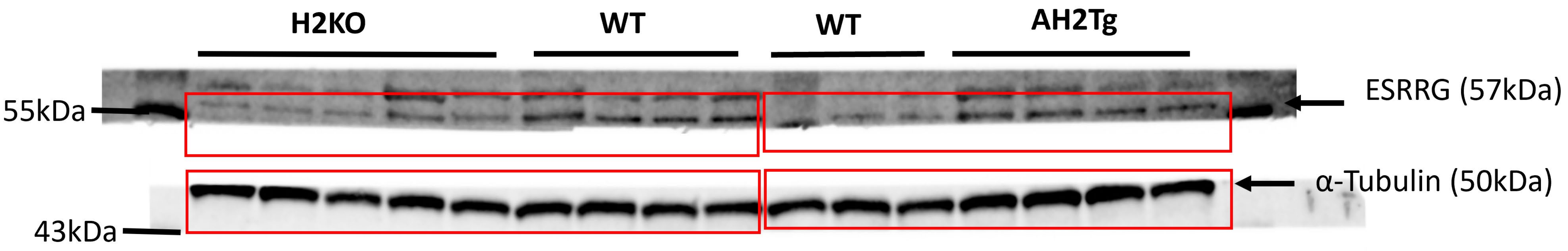

Suppl. Fig 20J

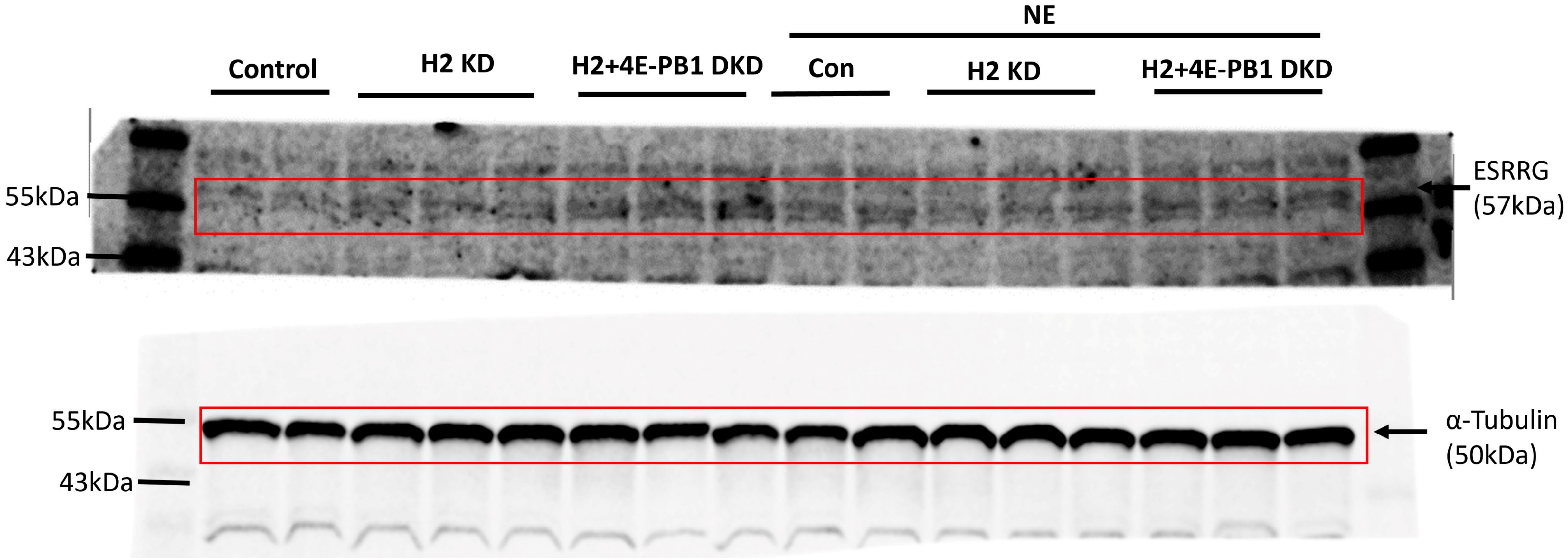

Supplement: Unedited blot and gel images [file jciinsight-9-164771-s143.pdf]
